# Supplementary material for: Pathophysiology of COVID-19: A Post Hoc Analysis of the ICAT-COVID Clinical Trial of the Bradykinin Antagonist Icatibant
Source: Pathogens. 2025 May 27;14(6):533. doi: 10.3390/pathogens14060533 (PMC12196139; doi:10.3390/pathogens14060533)
Supplement: Supplementary file 1 [file pathogens-14-00533-s001.zip › pathogens-3653727-supplementary.pdf]

Supplementary Materials to:

Pathophysiology of COVID-19: A post-hoc analysis of the ICAT-COVID clinical trial of the bradykinin antagonist icatibant

Pierre Malchair, Jordi Giol, Javier Jacob, Jesús Villoria, Thiago Carnaval, and Sebastián Videla

ICAT·COVID Group

A post hoc analysis of COVID-19 pathophysiology based on the ICAT·COVID clinical trial of the bradykinin antagonist Icatibant

Pierre Malchair,[1] Jordi Giol,[1] Javier Jacob,[1] Jesús Villoria,[2,3] Thiago Carnaval,[2,3] and Sebastián Videla,[4,5,6]

[1] Emergency Department, Bellvitge University Hospital, carrer de la Feixa Llarga s/n, 08907 L'Hospitalet de Llobregat, Barcelona, Spain

[2] Design and Biometrics Department, Medicxact, plaza Ermita 4, 28430 Alpedrete, Madrid, Spain

[3] Neuropharmacology & Pain Group, Neuroscience Program, Bellvitge Institute for Biomedical Research (IDIBELL), 08907 L'Hospitalet de Llobregat, Barcelona, Spain

[4] Clinical Research Support Area, Clinical Pharmacology Department, Germans Trias i Pujol University Hospital, Carretera de Canyet, s/n, 08916 Badalona, Barcelona, Spain

[5] Pharmacology Unit, Department of Pathology and Experimental Therapeutics, School of Medicine and Health Sciences, Bellvitge Biomedical Research Institute (IDIBELL), University of Barcelona, carrer de la Feixa Llarga s/n, 08907 L'Hospitalet de Llobregat, Barcelona, Spain

[6] Fight Infectious Diseases Foundation, Badalona, Spain

Table of Contents

Supplementary Methods 12

Supplementary Results 13

Supplementary Tables 15

Table S1: Objective selection of markers that best discriminate between the clinical statuses by means of partial least squares projections to latent structures regression 15

Supplementary Figures 19

Figure S1: Individual raw and adjusted trajectories of serum interleukin 6 levels of five patients taken at random 19

Figure S2: Individual raw and adjusted trajectories of serum ferritin levels of five patients taken at random 20

Figure S3: Individual raw and adjusted trajectories of serum lactate dehydrogenase levels of five patients taken at random 21

Figure S4: Individual raw and adjusted trajectories of serum C reactive protein levels of five patients taken at random 22

Figure S5: Individual raw and adjusted trajectories of serum D dimer levels of five patients taken at random 23

Figure S6: Individual raw and adjusted trajectories of serum C1 esterase inhibitor levels (antigenic protein test) of five patients taken at random 24

Figure S7: Individual raw and adjusted trajectories of serum C1 esterase inhibitor activity (functional test) of five patients taken at random 25

Figure S8: Individual raw and adjusted trajectories of serum C4 factor levels (functional test) of five patients taken at random 26

Figure S9: Individual raw and adjusted trajectories of blood lymphocyte count of five patients taken at random 27

Figure S10: Adjusted means and inferences from the generalized linear mixed models of serum interleukin 6 levels over the subgroups defined by dichotomic clinical milestones 28

Figure S11: Adjusted means and inferences from the generalized linear mixed models of serum ferritin levels over the subgroups defined by dichotomic clinical milestones........ 29

Figure S12: Adjusted means and inferences from the generalized linear mixed models of serum lactate dehydrogenase levels over the subgroups defined by dichotomic clinical milestones........ 30

Figure S13: Adjusted means and inferences from the generalized linear mixed models of serum C reactive protein levels over the subgroups defined by dichotomic clinical milestones 31

Figure S14: Adjusted means and inferences from the generalized linear mixed models of serum D dimer levels over the subgroups defined by dichotomic clinical milestones........ 32

Figure S15: Adjusted means and inferences from the generalized linear mixed models of serum C1 esterase inhibitor levels (antigenic protein test) over the subgroups defined by dichotomic clinical milestones 33

Figure S16: Adjusted means and inferences from the generalized linear mixed models of serum C1 esterase inhibitor activity (functional test) over the subgroups defined by dichotomic clinical milestones 34

Figure S17: Adjusted means and inferences from the generalized linear mixed models of serum C4 factor levels over the subgroups defined by dichotomic clinical milestones........ 35

Figure S18: Adjusted means and inferences from the generalized linear mixed models of blood lymphocyte counts over the subgroups defined by dichotomic clinical milestones 36

Figure S19: Concurrent criterion-type validity of the discriminative ability of interleukin 6 to discern between patients who did and did not achieve a WHO status score scale <4 at Day 10

(Visit 5), both unadjusted (empirical ROC curve) and adjusted by either Icatibant treatment or the time elapsed from symptom onset to hospitalization (binormal induced smooth ROC curves) 37

Figure S20: Concurrent criterion-type validity of the discriminative ability of interleukin 6 to discern between patients who had and had not been discharged at Day 10 (Visit 5), both unadjusted (empirical ROC curve) and adjusted by either Icatibant treatment or the time elapsed from symptom onset to hospitalization (binormal induced smooth ROC curves) 38

Figure S21: Concurrent criterion-type validity of the discriminative ability of interleukin 6 to discern between patients who did and did not attain a median $\mathbf{SpO2/FiO2}$ ratio >380 at Day 4 (Visit 4), both unadjusted (empirical ROC curve) and adjusted by either Icatibant treatment or the time elapsed from symptom onset to hospitalization (binormal induced smooth ROC curves) 39

Figure S22: Concurrent criterion-type validity of the discriminative ability of interleukin 6 to discern between patients who did and did not attain a median $\mathbf{SpO2/FiO2}$ ratio >380 at Day 10 (Visit 5), both unadjusted (empirical ROC curve) and adjusted by either Icatibant treatment or the time elapsed from symptom onset to hospitalization (binormal induced smooth ROC curves) 40

Figure S23: Concurrent criterion-type validity of the discriminative ability of interleukin 6 to discern between patients who did and did not attain a median Rox index >25 at Day 4 (Visit 4), both unadjusted (empirical ROC curve) and adjusted by either Icatibant treatment or the time elapsed from symptom onset to hospitalization (binormal induced smooth ROC curves) . 41

Figure S24: Concurrent criterion-type validity of the discriminative ability of interleukin 6 to discern between patients who did and did not attain a median Rox index >25 at Day 10 (Visit 5), both unadjusted (empirical ROC curve) and adjusted by either Icatibant treatment or the time elapsed from symptom onset to hospitalization (binormal induced smooth ROC curves) . 42

Figure S25: Concurrent criterion-type validity of the discriminative ability of interleukin 6 to discern between patients who did and did not attain a clinical response at Day 10 (Visit 5), both unadjusted (empirical ROC curve) and adjusted by either Icatibant treatment or the time elapsed from symptom onset to hospitalization (binormal induced smooth ROC curves) 43

Figure S26: Concurrent criterion-type validity of the discriminative ability of interleukin 6 to discern between patients who did and did not attain a clinical response 28 days after initial discharge, both unadjusted (empirical ROC curve) and adjusted by either Icatibant treatment or the time elapsed from symptom onset to hospitalization (binormal induced smooth ROC curves) 44

Figure S27: Concurrent criterion-type validity of the discriminative ability of interleukin 6 to discern patients who died from COVID-19, both unadjusted (empirical ROC curve) and adjusted by either Icatibant treatment or the time elapsed from symptom onset to hospitalization (binormal induced smooth ROC curves) 45

Figure S28: Concurrent criterion-type validity of the discriminative ability of interleukin 6 to discern patients who died from any reason, both unadjusted (empirical ROC curve) and adjusted by either Icatibant treatment or the time elapsed from symptom onset to hospitalization (binormal induced smooth ROC curves) 46

Figure S29: Concurrent criterion-type validity of the discriminative ability of ferritin to discern between patients who did and did not attain a median $\mathbf{SpO2/FiO2}$ ratio >380 at Day 4 (Visit 4), both unadjusted (empirical ROC curve) and adjusted by either Icatibant treatment or the time elapsed from symptom onset to hospitalization (binormal induced smooth ROC curves) 47

Figure S30: Concurrent criterion-type validity of the discriminative ability of ferritin to discern between patients who did and did not attain a median Rox index >25 at Day 4 (Visit 4), both unadjusted (empirical ROC curve) and adjusted by either Icatibant treatment or the time elapsed from symptom onset to hospitalization (binormal induced smooth ROC curves) . 48

Figure S31: Concurrent criterion-type validity of the discriminative ability of C reactive protein to discern between patients who did and did not attain a median $SpO2/FiO2$ ratio >380 at Day 4 (Visit 4), both unadjusted (empirical ROC curve) and adjusted by either Icatibant treatment or the time elapsed from symptom onset to hospitalization (binormal induced smooth ROC curves) 49

Figure S32: Concurrent criterion-type validity of the discriminative ability of C reactive protein to discern between patients who did and did not attain a median Rox index >25 at Day 4 (Visit 4), both unadjusted (empirical ROC curve) and adjusted by either Icatibant treatment or the time elapsed from symptom onset to hospitalization (binormal induced smooth ROC curves) 50

Figure S33: Concurrent criterion-type validity of the discriminative ability of C reactive protein to discern between patients who did and did not attain a median Rox index >25 at Day 10 (Visit 5), both unadjusted (empirical ROC curve) and adjusted by either Icatibant treatment or the time elapsed from symptom onset to hospitalization (binormal induced smooth ROC curves) 51

Figure S34: Concurrent criterion-type validity of the discriminative ability of D dimer to discern between patients who did and did not attain a median $SpO2/FiO2$ ratio >380 at Day 4 (Visit 4), both unadjusted (empirical ROC curve) and adjusted by either Icatibant treatment or the time elapsed from symptom onset to hospitalization (binormal induced smooth ROC curves) 52

Figure S35: Concurrent criterion-type validity of the discriminative ability of C1 esterase inhibitor (antigenic protein test) between patients who did and did not attain a median $SpO2/FiO2$ ratio >380 at Day 4 (Visit 4), both unadjusted (empirical ROC curve) and adjusted by either Icatibant treatment or the time elapsed from symptom onset to hospitalization (binormal induced smooth ROC curves) 53

Figure S36: Concurrent criterion-type validity of the discriminative ability of C4 complement factor between patients who did and did not attain a median $SpO2/FiO2$ ratio >380 at Day 4 (Visit 4), both unadjusted (empirical ROC curve) and adjusted by either Icatibant treatment or the time elapsed from symptom onset to hospitalization (binormal induced smooth ROC curves) 54

Figure S37: Concurrent criterion-type validity of the discriminative ability of C4 complement factor between patients who did and did not attain a median Rox index >25 at Day 4 (Visit 4), both unadjusted (empirical ROC curve) and adjusted by either Icatibant treatment or the time elapsed from symptom onset to hospitalization (binormal induced smooth ROC curves) 55

Figure S38: Concurrent criterion-type validity of the discriminative ability of LDH to discern between patients who did and did not achieve a WHO status score scale <4 at Day 10 (Visit 5), both unadjusted (empirical ROC curve) and adjusted by either Icatibant treatment or the time elapsed from symptom onset to hospitalization (binormal induced smooth ROC curves) . 56

Figure S39: Concurrent criterion-type validity of the discriminative ability of LDH to discern between patients who had and had not been discharged at Day 10 (Visit 5), both unadjusted (empirical ROC curve) and adjusted by either Icatibant treatment or the time elapsed from symptom onset to hospitalization (binormal induced smooth ROC curves) 57

Figure S40: Concurrent criterion-type validity of the discriminative ability of LDH to discern between patients who did and did not attain a median $SpO2/FiO2$ ratio >380 at Day 4 (Visit 4), both unadjusted (empirical ROC curve) and adjusted by either Icatibant treatment or the time elapsed from symptom onset to hospitalization (binormal induced smooth ROC curves) . 58

Figure S41: Concurrent criterion-type validity of the discriminative ability of LDH to discern between patients who did and did not attain a median $SpO2/FiO2$ ratio >380 at Day 10 (Visit 5), both unadjusted (empirical ROC curve) and adjusted by either Icatibant treatment or the time elapsed from symptom onset to hospitalization (binormal induced smooth ROC curves) . 59

Figure S42: Concurrent criterion-type validity of the discriminative ability of LDH to discern between patients who did and did not attain a Rox index >25 at Day 10 (Visit 5), both unadjusted (empirical ROC curve) and adjusted by either Icatibant treatment or the time elapsed from symptom onset to hospitalization (binormal induced smooth ROC curves) 60

Figure S43: Concurrent criterion-type validity of the discriminative ability of LDH to discern between patients who did and did not attain a clinical response at Day 10 (Visit 5), both unadjusted (empirical ROC curve) and adjusted by either Icatibant treatment or the time elapsed from symptom onset to hospitalization (binormal induced smooth ROC curves) 61

Figure S44: Concurrent criterion-type validity of the discriminative ability of LDH to discern between patients who did and did not attain a clinical response 28 days after initial discharge, both unadjusted (empirical ROC curve) and adjusted by either Icatibant treatment or the time elapsed from symptom onset to hospitalization (binormal induced smooth ROC curves) 62

Figure S45: Concurrent criterion-type validity of the discriminative ability of LDH to discern patients who died from COVID-19, both unadjusted (empirical ROC curve) and adjusted by either Icatibant treatment or the time elapsed from symptom onset to hospitalization (binormal induced smooth ROC curves) 63

Figure S46: Concurrent criterion-type validity of the discriminative ability of LDH to discern patients who died from any reason, both unadjusted (empirical ROC curve) and adjusted by either Icatibant treatment or the time elapsed from symptom onset to hospitalization (binormal induced smooth ROC curves) 64

Figure S47: Concurrent criterion-type validity of the discriminative ability of lymphocytes to discern between patients who did and did not achieve a WHO status score scale <4 at Day 10 (Visit 5), both unadjusted (empirical ROC curve) and adjusted by either Icatibant treatment or the time elapsed from symptom onset to hospitalization (binormal induced smooth ROC curves) 65

Figure S48: Concurrent criterion-type validity of the discriminative ability of lymphocytes to discern between patients who had and had not been discharged at Day 10 (Visit 5), both unadjusted (empirical ROC curve) and adjusted by either Icatibant treatment or the time elapsed from symptom onset to hospitalization (binormal induced smooth ROC curves) 66

Figure S49: Concurrent criterion-type validity of the discriminative ability of lymphocytes to discern between patients who did and did not attain a median **SpO2/FiO2** ratio >380 at Day 4 (Visit 4), both unadjusted (empirical ROC curve) and adjusted by either Icatibant treatment or the time elapsed from symptom onset to hospitalization (binormal induced smooth ROC curves) 67

Figure S50: Concurrent criterion-type validity of the discriminative ability of lymphocytes to discern between patients who did and did not attain a median **SpO2/FiO2** ratio >380 at Day 10 (Visit 5), both unadjusted (empirical ROC curve) and adjusted by either Icatibant treatment or the time elapsed from symptom onset to hospitalization (binormal induced smooth ROC curves) 68

Figure S51: Concurrent criterion-type validity of the discriminative ability of lymphocytes to discern between patients who did and did not attain a median Rox index >25 at Day 4 (Visit 4), both unadjusted (empirical ROC curve) and adjusted by either Icatibant treatment or the time elapsed from symptom onset to hospitalization (binormal induced smooth ROC curves) . 69

Figure S52: Concurrent criterion-type validity of the discriminative ability of lymphocytes to discern between patients who did and did not attain a median Rox index >25 at Day 10 (Visit 5), both unadjusted (empirical ROC curve) and adjusted by either Icatibant treatment or the time elapsed from symptom onset to hospitalization (binormal induced smooth ROC curves) . 70

Figure S53: Concurrent criterion-type validity of the discriminative ability of lymphocytes to discern between patients who did and did not attain a clinical response at Day 10 (Visit 5), both unadjusted (empirical ROC curve) and adjusted by either Icatibant treatment or the time elapsed from symptom onset to hospitalization (binormal induced smooth ROC curves) 71

Figure S54: Concurrent criterion-type validity of the discriminative ability of lymphocytes to discern between patients who did and did not attain a clinical response 28 days after initial discharge, both unadjusted (empirical ROC curve) and adjusted by either Icatibant treatment or the time elapsed from symptom onset to hospitalization (binormal induced smooth ROC curves) 72

Figure S55: Concurrent criterion-type validity of the discriminative ability of lymphocytes to discern between patients who died from COVID-19, both unadjusted (empirical ROC curve) and adjusted by either Icatibant treatment or the time elapsed from symptom onset to hospitalization (binormal induced smooth ROC curves) 73

Figure S56: Concurrent criterion-type validity of the discriminative ability of lymphocytes to discern between patients who died from any reason, both unadjusted (empirical ROC curve) and adjusted by either Icatibant treatment or the time elapsed from symptom onset to hospitalization (binormal induced smooth ROC curves) 74

Supplementary Methods

Study Outcomes

Outcomes included clinical response at visit 5 (V5) (defined as being discharged for at least 48 consecutive hours in the absence of severe (grade ≥3) adverse reactions according to the Common Toxicity Criteria for Adverse Events by study day 10) and clinical efficacy (staying out of hospital) 28 days after the initial discharge, time to supplemental oxygen cessation and hospital discharge, COVID-19-related and all-cause mortality, and safety.

Statistical Analyses

Binormal ROC curves were in general estimated by probit regression of the ROC representation in terms of the enumerator functions: $\mathrm{ROC}(t) = S_D\left(S_{\bar{D}}^{-1}(t)\right)$ over the quartiles of $S_{\bar{D}}(Y_{\bar{D}})$, which correspond to the space of possible values of the false positive fraction in the sample: $\Phi^{-1}\left[S_{\bar{D}}\left(S_{\bar{D}}^{-1}(t)\right)\right] = \gamma_0 + \gamma_1\Phi^{-1}(t)$. Inferences were made using the asymptotic expression V_1/n_D for the variance $var\left[\widehat{\mathrm{ROC}}(t)\right]$, where $V_1 = \mathrm{ROC}(t)\left(1 - \mathrm{ROC}(t)\right) + \kappa\tau_1^2 T(1 - T)$ [1], being n_D and $n_{\bar{D}}$ the numbers of patients who met and did not met, respectively, the clinical milestone of interest, κ the proportion between these $(n_D/n_{\bar{D}})$, and τ_1 the slope of the ROC curve under the alternative, in this case estimated by γ_1.

Since the analysis of the factors influencing concurrent validity for a continuous marker constitutes a bidimensional problem, to identify the influence of Icatibant treatment and the time since symptom onset, an evaluation of: a) the influence over the distribution of false positives (among patients who did not attain the clinical milestone of interest), and b) the influence over the ROC curve itself, were done separately. In all instances, the binormal ROC parametric model ($\mathrm{ROC}(t) = \Phi\left(\gamma_0 + \gamma_1\Phi^{-1}(t)\right)$) was used for simplicity. This binormal model provides a robust parametrization of empirical ROC curves in the sense that it does not require that marker results tightly follow a Normal distribution, because (empirical ROC curves) pertain to the relationships between the (empirical) survival functions of observed marker values between the negative (healthy, “non-diseased”) and positive (“diseased”) populations rather than to the distributions of marker results themselves [2].

For the first of these subtasks, compound models were employed to model the results of the marker in the form:

$$Y = \alpha_0 + \alpha_1 D + \boldsymbol{\alpha_2 X} + \boldsymbol{\alpha_3 X} D + \sigma(D)\epsilon, \qquad [1]$$

where $\varepsilon \sim N(0,1)$, $\sigma(D) = \sigma_D I[D = 1] + \sigma_{\bar{D}} I[D = 0]$ and D indicates the attainment of the clinical milestone. Note that this model allows for different variances of the distributions of the marker between patients who attained and did not attain the clinical milestones (although not for the different levels of covariate values). In addition, since we used the Normal distribution for errors (ε) and used identity link functions, we assumed that marker results admit Normal parametrization, which we deem that is not very unreasonable in view of the results of the adjusted analyses (see the Figures S10 to S18 below).

For the second subtask, using the estimates of $\boldsymbol{\alpha}$, σ_D and $\sigma_{\bar{D}}$ obtained above, the induced ROC curve was calculated as [1]:

$$\mathrm{ROC}_Z(t) = \Phi\left(\frac{\alpha_1}{\sigma_D} + \frac{\boldsymbol{\alpha_3}}{\sigma_D} + \frac{\sigma_{\bar{D}}}{\sigma_D}\Phi^{-1}(t)\right), \qquad [2]$$

where *t* denotes the fraction of false positives. Note that the model in [1] allows checking whether the covariates provoke any effects over the classification brought by the marker through the parameter α_2, whilst the parameter α_3 enables testing whether the covariates have an influence over the ROC curve (that is, the separation between S_D and $S_{\bar{D}}$).

Statistical inferences were two-sided by default. All analyses were performed using SAS version 9.4 (SAS Institute Inc., Cary, NC, USA).

Supplementary Results

Main Clinical Results

Clinical response at V5 was more frequent in the active group than in the control group; yet, this difference did not reach significance (risk difference [RD], 17.4; 95% confidence interval [CI], −4.2 to

39.1; p=0.115). On the other hand, the difference in clinical efficacy 28 days after initial discharge between the active and control groups (37 out of 37, 100%, and 30 out of 36, 83.3%, respectively) was significant (RD, 16.7; 95% CI 4.5 to 28.8; p=0.011). COVID-19-related and all-cause mortality also differed significantly as none patient died in the active group compared to five (13.9%; RD, 13.9; 95% CI 2.6 to 25.2; p=0.025) and six (16.7%; RD, 16.7; 95% CI 4.5 to 28.8; p=0.011) patients, respectively, in the control group. See the main publication of the ICAT·COVID trial [3] for more details.

Supplementary Tables

Table S1: Objective selection of markers that best discriminate between the clinical statuses by means of partial least squares projections to latent structures regression

Marker (independent variables)	Milestone (dependent variables)	VIP	Selected
IL-6	WHO score V5 <4 vs. ≥ 4	1.77257	Yes
IL-6	Discharge ≤V5 vs. >V5	1.70887	Yes
IL-6	Pa/Fi V4 >380 vs. ≤380	1.43128	Yes
IL-6	Pa/Fi V5 >380 vs. ≤380	1.49219	Yes
IL-6	ROX index V4 >25 vs. ≤25	1.32913	Yes
IL-6	ROX index V5 >25 vs. ≤25	1.02161	Yes
IL-6	Clinical response at V5 vs. no response	1.70887	Yes
IL-6	Clinical efficacy at D28 vs. no efficacy	1.87787	Yes
IL-6	COVID-related death vs. no death	1.87787	Yes
IL-6	All-cause death vs. no death	1.87787	Yes
Ferritin	WHO score V5 <4 vs. ≥4	0.32121	No
Ferritin	Discharge ≤V5 vs. >V5	0.17617	No
Ferritin	Pa/Fi V4 >380 vs. ≤380	1.04715	Yes
Ferritin	Pa/Fi V5 >380 vs. ≤380	0.37734	No
Ferritin	ROX index V4 >25 vs. ≤25	1.12206	Yes
Ferritin	ROX index V5 >25 vs. ≤25	0.35776	No
Ferritin	Clinical response at V5 vs. no response	0.17617	No
Ferritin	Clinical efficacy at D28 vs. no efficacy	0.29766	No
Ferritin	COVID-related death vs. no death	0.29766	No
Ferritin	All-cause death vs. no death	0.29766	No
LDH	WHO score V5 <4 vs. ≥4	1.76520	Yes
LDH	Discharge ≤V5 vs. >V5	1.46195	Yes
LDH	Pa/Fi V4 >380 vs. ≤380	0.98578	Yes

LDH	Pa/Fi V5 >380 vs. ≤380	2.07325	Yes
LDH	ROX index V4 >25 vs. ≤25	0.75683	No
LDH	ROX index V5 >25 vs. ≤25	2.15054	Yes
LDH	Clinical response at V5 vs. no response	1.46195	Yes
LDH	Clinical efficacy at D28 vs. no efficacy	1.88927	Yes
LDH	COVID-related death vs. no death	1.88927	Yes
LDH	All-cause death vs. no death	1.88927	Yes
CRP	WHO score V5 <4 vs. ≥4	0.56554	No
CRP	Discharge ≤V5 vs. >V5	0.16983	No
CRP	Pa/Fi V4 >380 vs. ≤380	1.04832	Yes
CRP	Pa/Fi V5 >380 vs. ≤380	0.77538	No
CRP	ROX index V4 >25 vs. ≤25	0.91263	Yes
CRP	ROX index V5 >25 vs. ≤25	0.94606	Yes
CRP	Clinical response at V5 vs. no response	0.16983	No
CRP	Clinical efficacy at D28 vs. no efficacy	0.71581	No
CRP	COVID-related death vs. no death	0.71581	No
CRP	All-cause death vs. no death	0.71581	No
D dimer	WHO score V5 <4 vs. ≥4	0.51941	No
D dimer	Discharge ≤V5 vs. >V5	0.32273	No
D dimer	Pa/Fi V4 >380 vs. ≤380	0.94131	Yes
D dimer	Pa/Fi V5 >380 vs. ≤380	0.30261	No
D dimer	ROX index V4 >25 vs. ≤25	0.22439	No
D dimer	ROX index V5 >25 vs. ≤25	0.10387	No
D dimer	Clinical response at V5 vs. no response	0.32273	No
D dimer	Clinical efficacy at D28 vs. no efficacy	0.51760	No
D dimer	COVID-related death vs. no death	0.51760	No
D dimer	All-cause death vs. no death	0.51760	No

C1q inh. (antigenic)	WHO score V5 <4 vs. ≥4	0.43821	No
C1q inh. (antigenic)	Discharge ≤V5 vs. >V5	0.47350	No
C1q inh. (antigenic)	Pa/Fi V4 >380 vs. ≤380	0.76565	No
C1q inh. (antigenic)	Pa/Fi V5 >380 vs. ≤380	0.47179	No
C1q inh. (antigenic)	ROX index V4 >25 vs. ≤25	1.25649	Yes
C1q inh. (antigenic)	ROX index V5 >25 vs. ≤25	0.54831	No
C1q inh. (antigenic)	Clinical response at V5 vs. no response	0.47350	No
C1q inh. (antigenic)	Clinical efficacy at D28 vs. no efficacy	0.27735	No
C1q inh. (antigenic)	COVID-related death vs. no death	0.27735	No
C1q inh. (antigenic)	All-cause death vs. no death	0.27735	No
C1q inh. (functional)	WHO score V5 <4 vs. ≥4	0.32013	No
C1q inh. (functional)	Discharge ≤V5 vs. >V5	0.18598	No
C1q inh. (functional)	Pa/Fi V4 >380 vs. ≤380	0.27550	No
C1q inh. (functional)	Pa/Fi V5 >380 vs. ≤380	0.13587	No
C1q inh. (functional)	ROX index V4 >25 vs. ≤25	0.35756	No
C1q inh. (functional)	ROX index V5 >25 vs. ≤25	0.51761	No
C1q inh. (functional)	Clinical response at V5 vs. no response	0.18598	No
C1q inh. (functional)	Clinical efficacy at D28 vs. no efficacy	0.27429	No
C1q inh. (functional)	All-cause death vs. no death	0.27429	No
C1q inh. (functional)	COVID-related death vs. no death	0.27429	No
C4	WHO score V5 <4 vs. ≥4	0.39284	No
C4	Discharge ≤V5 vs. >V5	0.51669	No
C4	Pa/Fi V4 >380 vs. ≤380	0.89994	Yes
C4	Pa/Fi V5 >380 vs. ≤380	0.42721	No
C4	ROX index V4 >25 vs. ≤25	1.15525	Yes
C4	ROX index V5 >25 vs. ≤25	0.47857	No
C4	Clinical response at V5 vs. no response	0.51669	No

C4	Clinical efficacy at D28 vs. no efficacy	0.21953	No
C4	COVID-related death vs. no death	0.21953	No
C4	All-cause death vs. no death	0.21953	No
Lymphocytes	WHO score V5 <4 vs. ≥4	1.26508	Yes
Lymphocytes	Discharge ≤V5 vs. >V5	1.80352	Yes
Lymphocytes	Pa/Fi V4 >380 vs. ≤380	1.19420	Yes
Lymphocytes	Pa/Fi V5 >380 vs. ≤380	1.10286	Yes
Lymphocytes	ROX index V4 >25 vs. ≤25	1.21538	Yes
Lymphocytes	ROX index V5 >25 vs. ≤25	1.22478	Yes
Lymphocytes	Clinical response at V5 vs. no response	1.80352	Yes
Lymphocytes	Clinical efficacy at D28 vs. no efficacy	0.91379	Yes
Lymphocytes	COVID-related death vs. no death	0.91379	Yes
Lymphocytes	All-cause death vs. no death	0.91379	Yes

Abbreviations: CRP, C reactive protein; C1q inh., C1q inhibitor; C4, complement factor 4; D28, day 28 after initial discharge; LDH, lactate dehydrogenase; IL-6, interleukin 6; Pa/Fi, quotient between partial (arterial) oxygen pressure and inspiratory oxygen fraction; V4, visit 4; V5, visit 5; VIP, variable influence on projection.

Supplementary Figures

Figure S1: Individual raw and adjusted trajectories of serum interleukin 6 levels of five patients taken at random

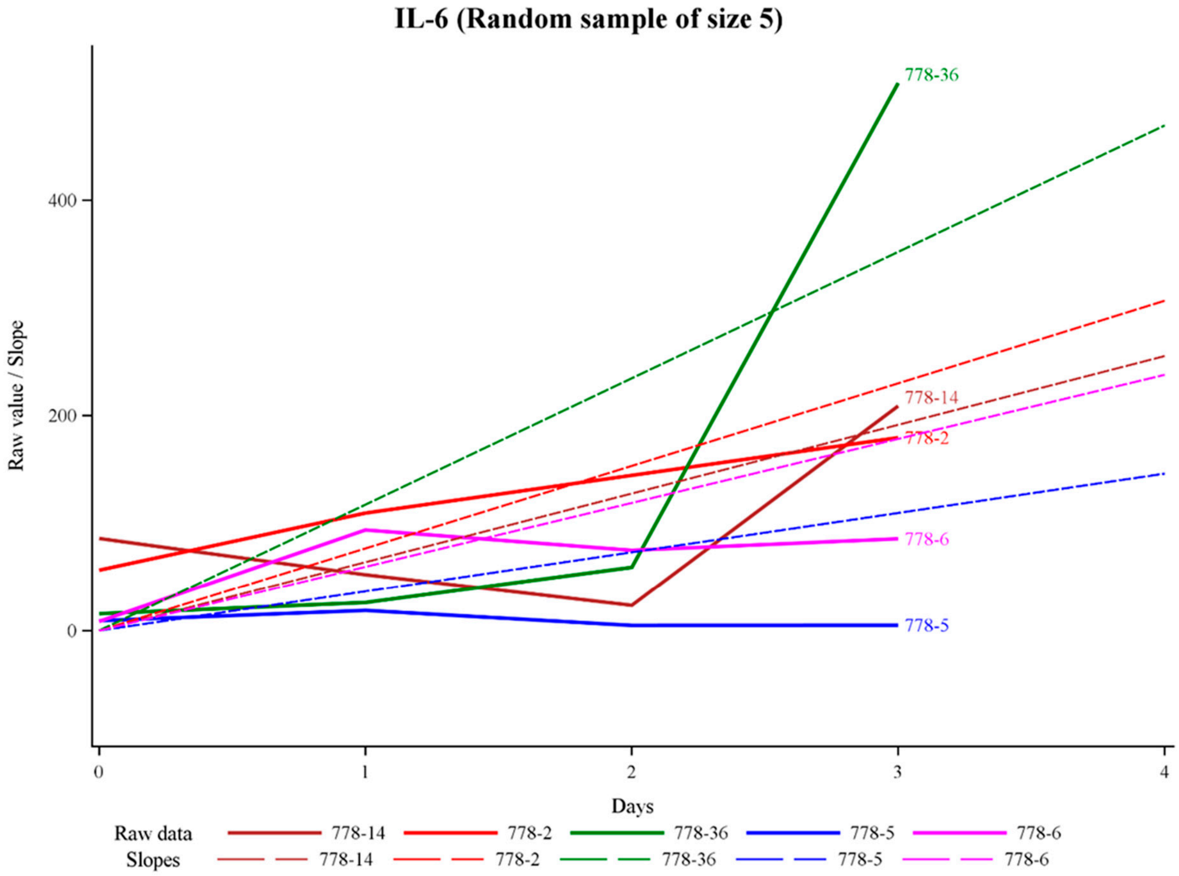

Abbreviations: IL, interleukin. The units in the ordinate axis are picograms per milliliter.

Figure S2: Individual raw and adjusted trajectories of serum ferritin levels of five patients taken at random

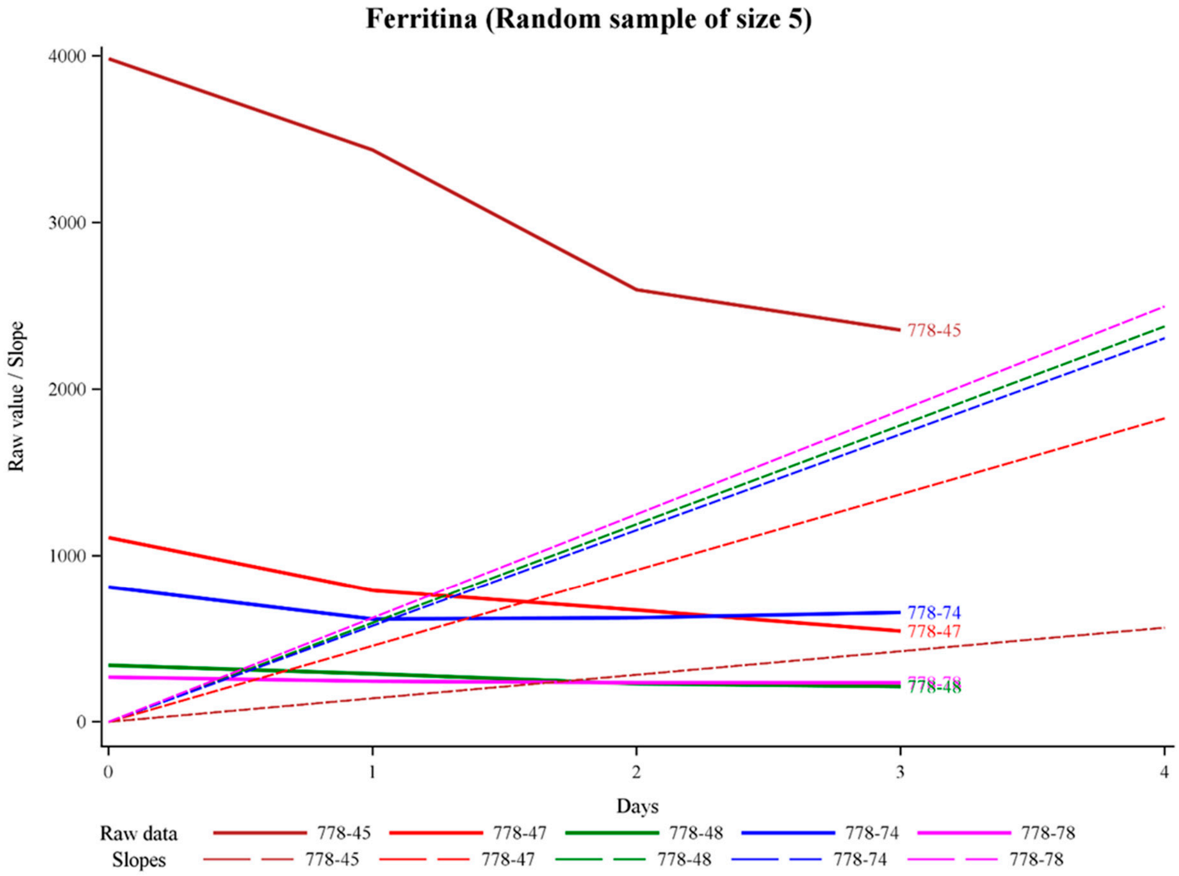

The units in the ordinate axis are nanograms per milliliter.

Figure S3: Individual raw and adjusted trajectories of serum lactate dehydrogenase levels of five patients taken at random

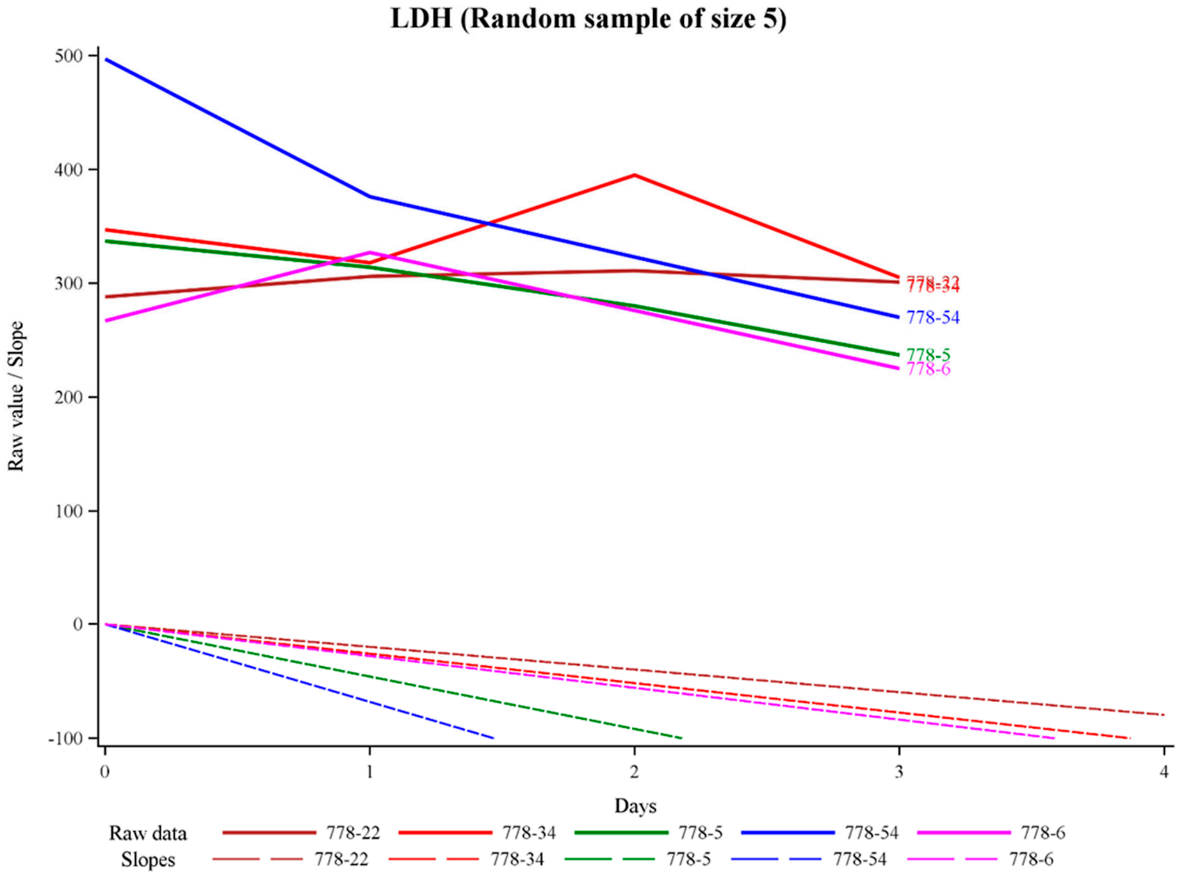

Abbreviations: LDH, lactate dehydrogenase. The units in the ordinate axis are international units per liter.

Figure S4: Individual raw and adjusted trajectories of serum C reactive protein levels of five patients taken at random

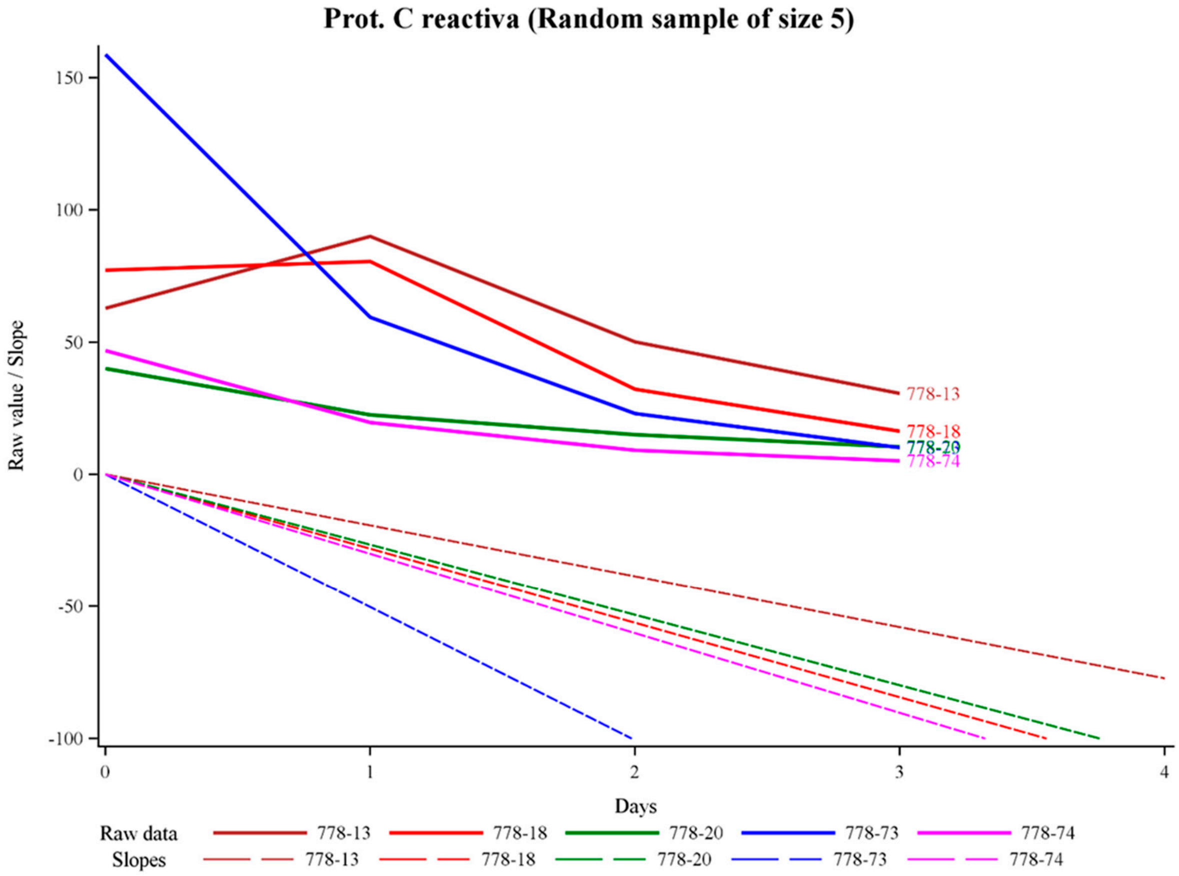

The units in the ordinate axis are milligrams per liter.

Figure S5: Individual raw and adjusted trajectories of serum D dimer levels of five patients taken at random

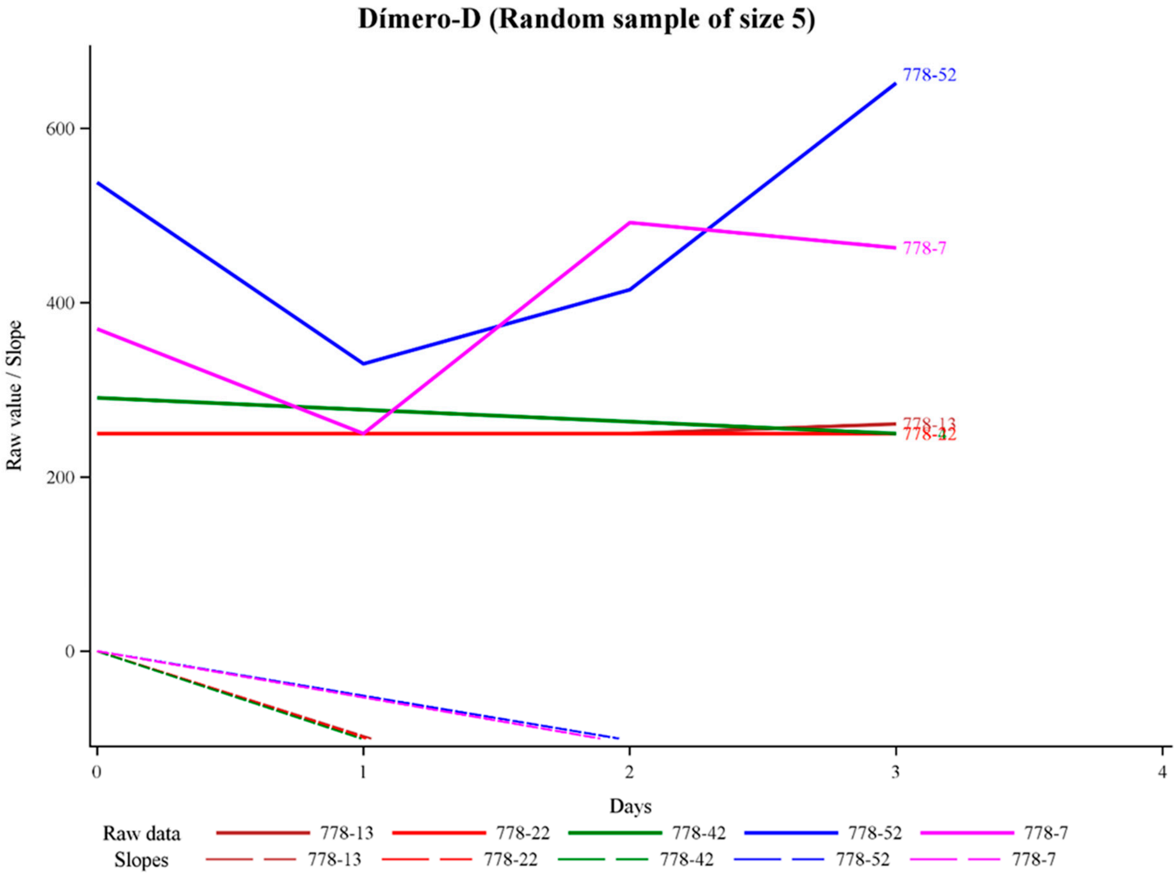

The units in the ordinate axis are micrograms per liter.

Figure S6: Individual raw and adjusted trajectories of serum C1 esterase inhibitor levels (antigenic protein test) of five patients taken at random

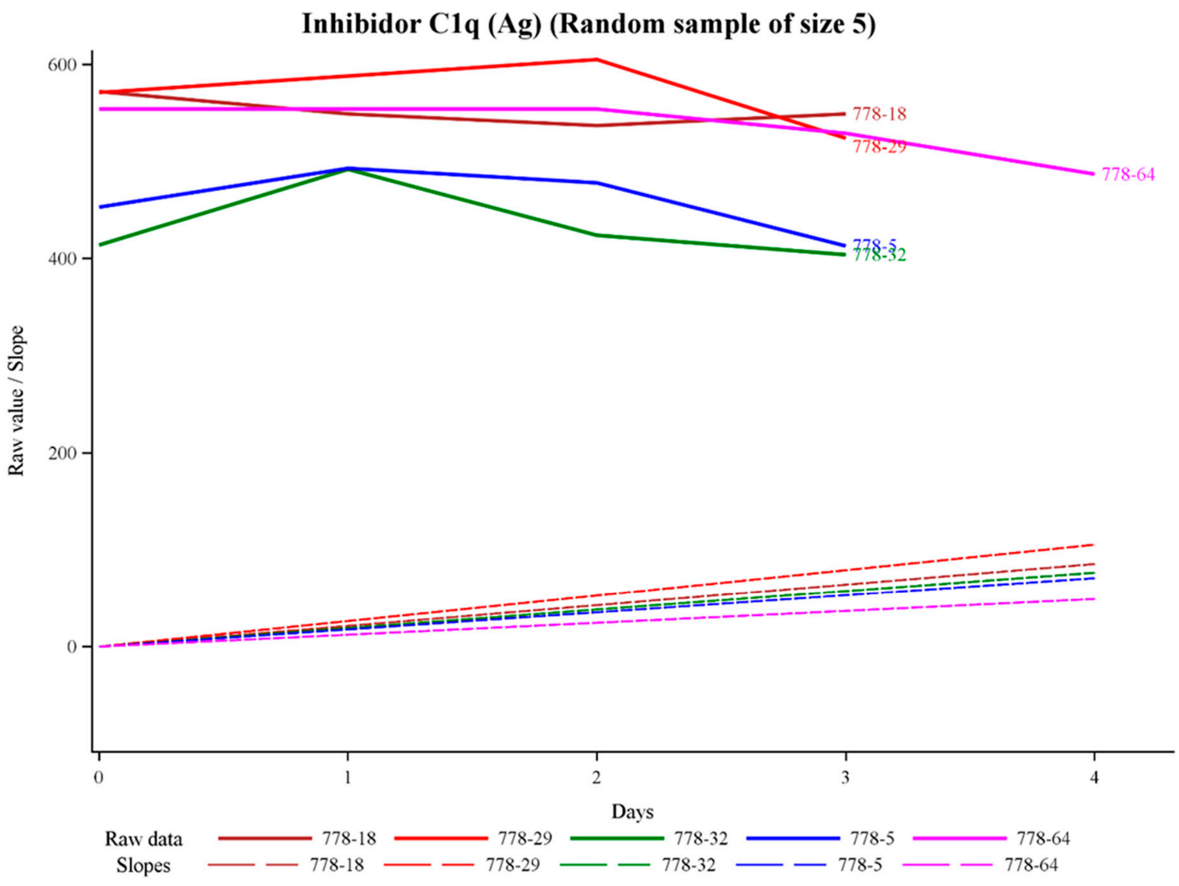

Abbreviations: C, complement. The units in the ordinate axis are milligrams per liter.

Figure S7: Individual raw and adjusted trajectories of serum C1 esterase inhibitor activity (functional test) of five patients taken at random

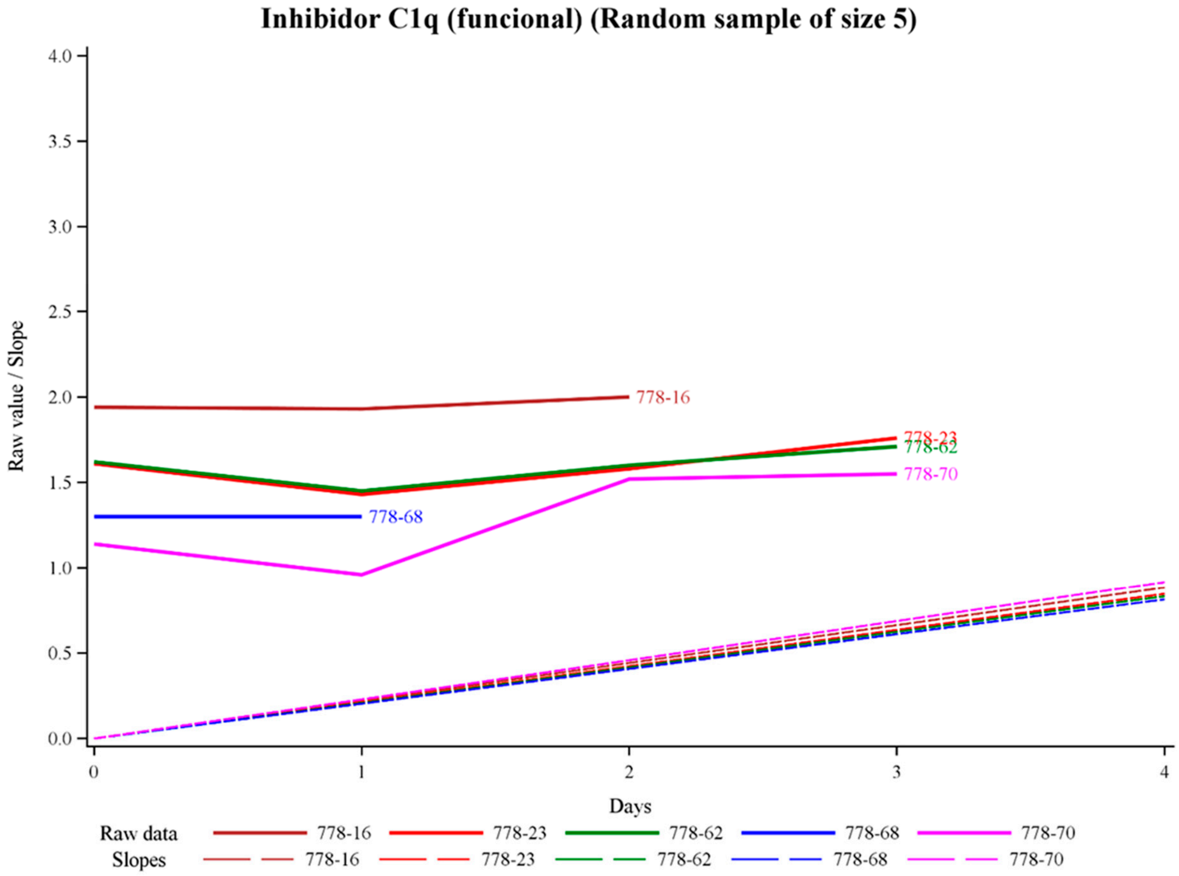

Abbreviations: C, complement. The units in the ordinate axis are unitless ratios.

Figure S8: Individual raw and adjusted trajectories of serum C4 factor levels (functional test) of five patients taken at random

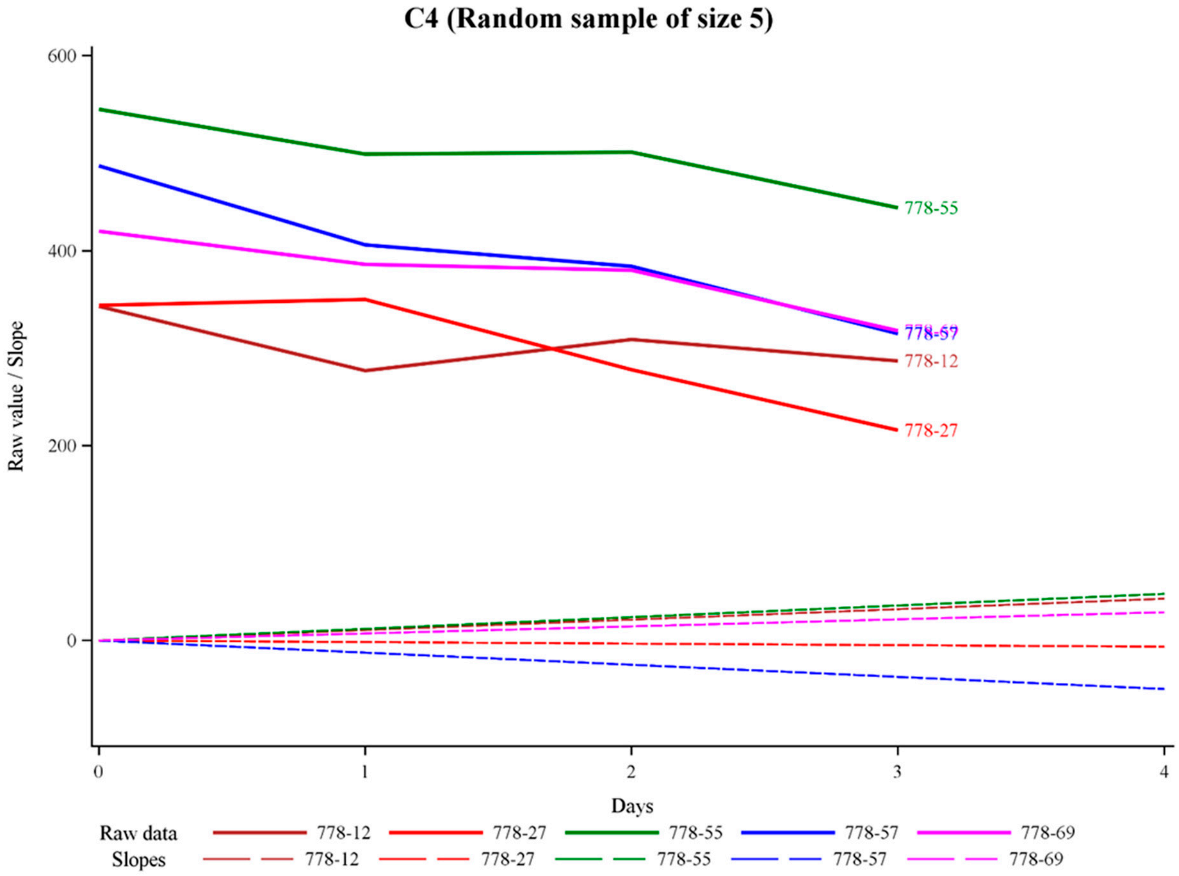

Abbreviations: C, complement. The units in the ordinate axis are milligrams per liter.

Figure S9: Individual raw and adjusted trajectories of blood lymphocyte count of five patients taken at random

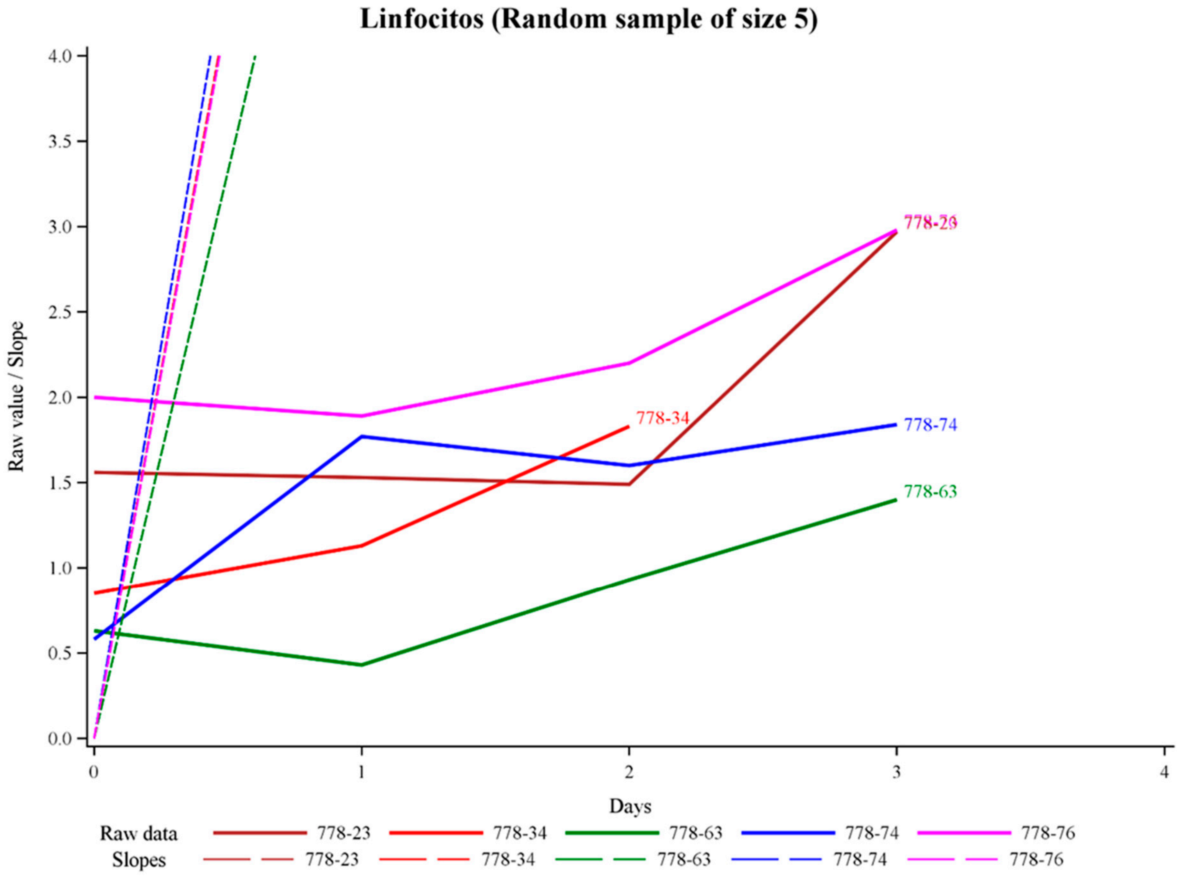

The units in the ordinate axis are counts per nanoliter. The slopes are multiplied by 10 for convenience in subsequent analyses.

Figure S10: Adjusted means and inferences from the generalized linear mixed models of serum interleukin 6 levels over the subgroups defined by dichotomic clinical milestones

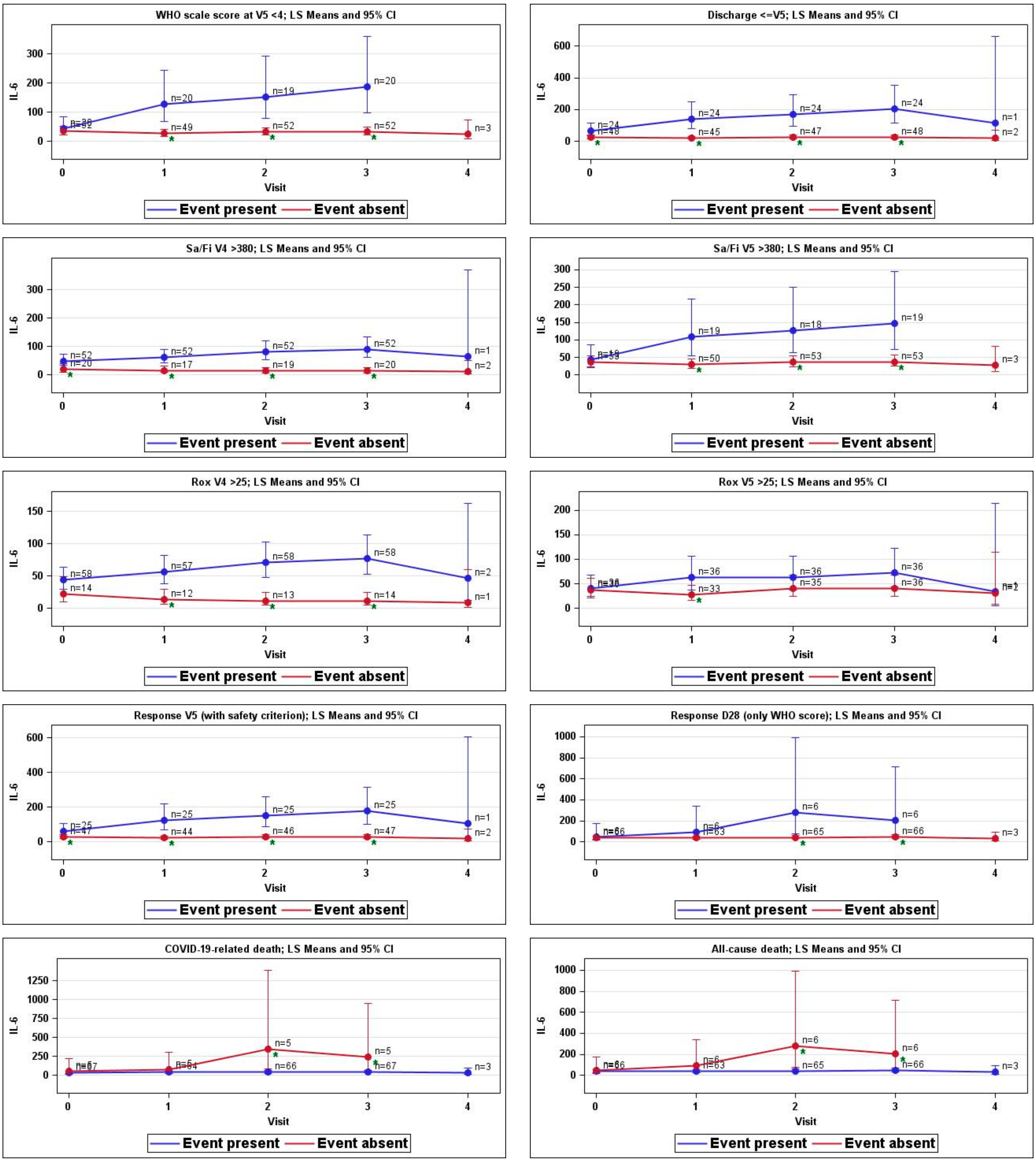

Abbreviations: IL, interleukin. The units in the ordinate axis are picograms per milliliter.

Figure S11: Adjusted means and inferences from the generalized linear mixed models of serum ferritin levels over the subgroups defined by dichotomic clinical milestones

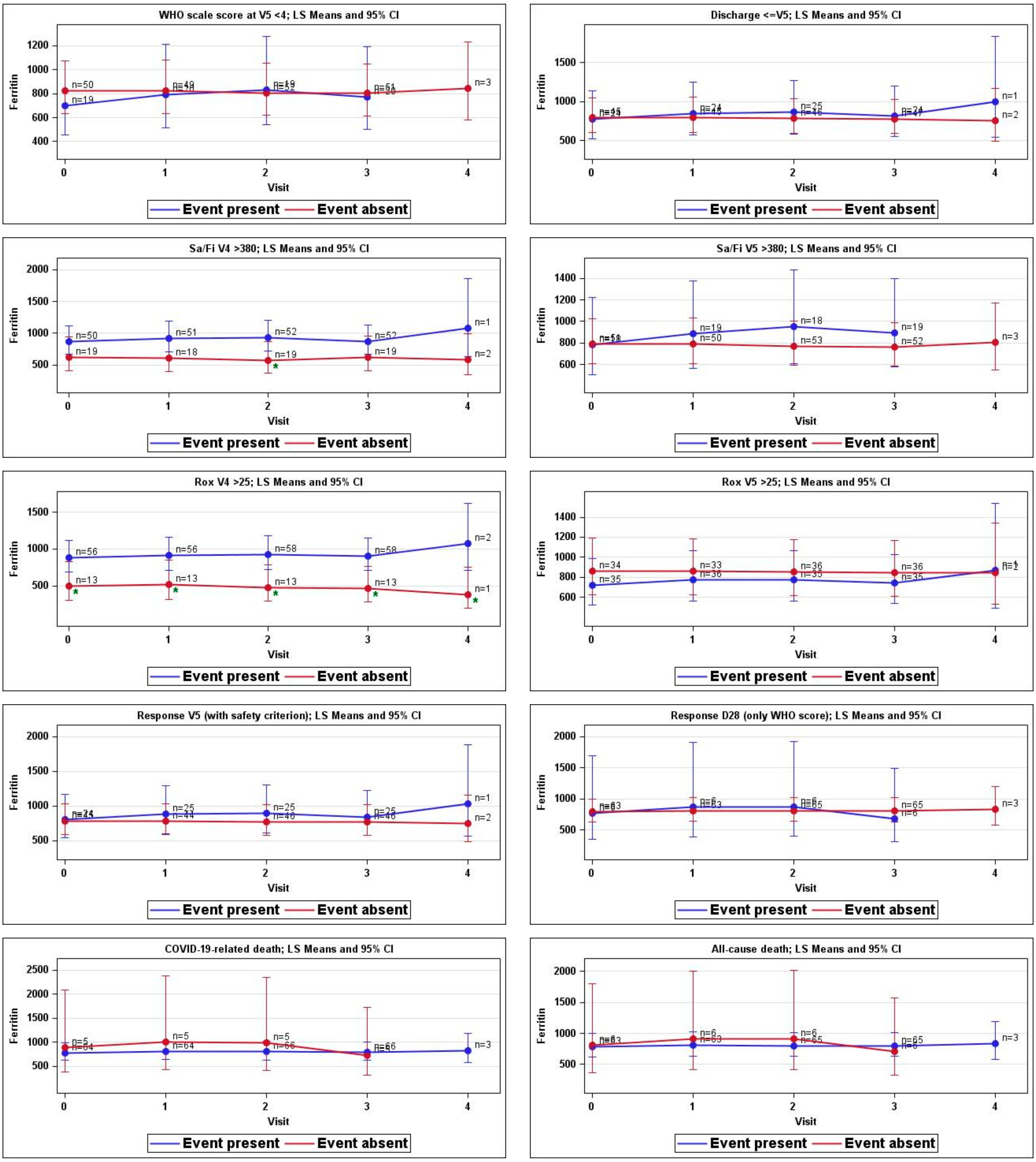

The units in the ordinate axis are nanograms per milliliter.

Figure S12: Adjusted means and inferences from the generalized linear mixed models of serum lactate dehydrogenase levels over the subgroups defined by dichotomic clinical milestones

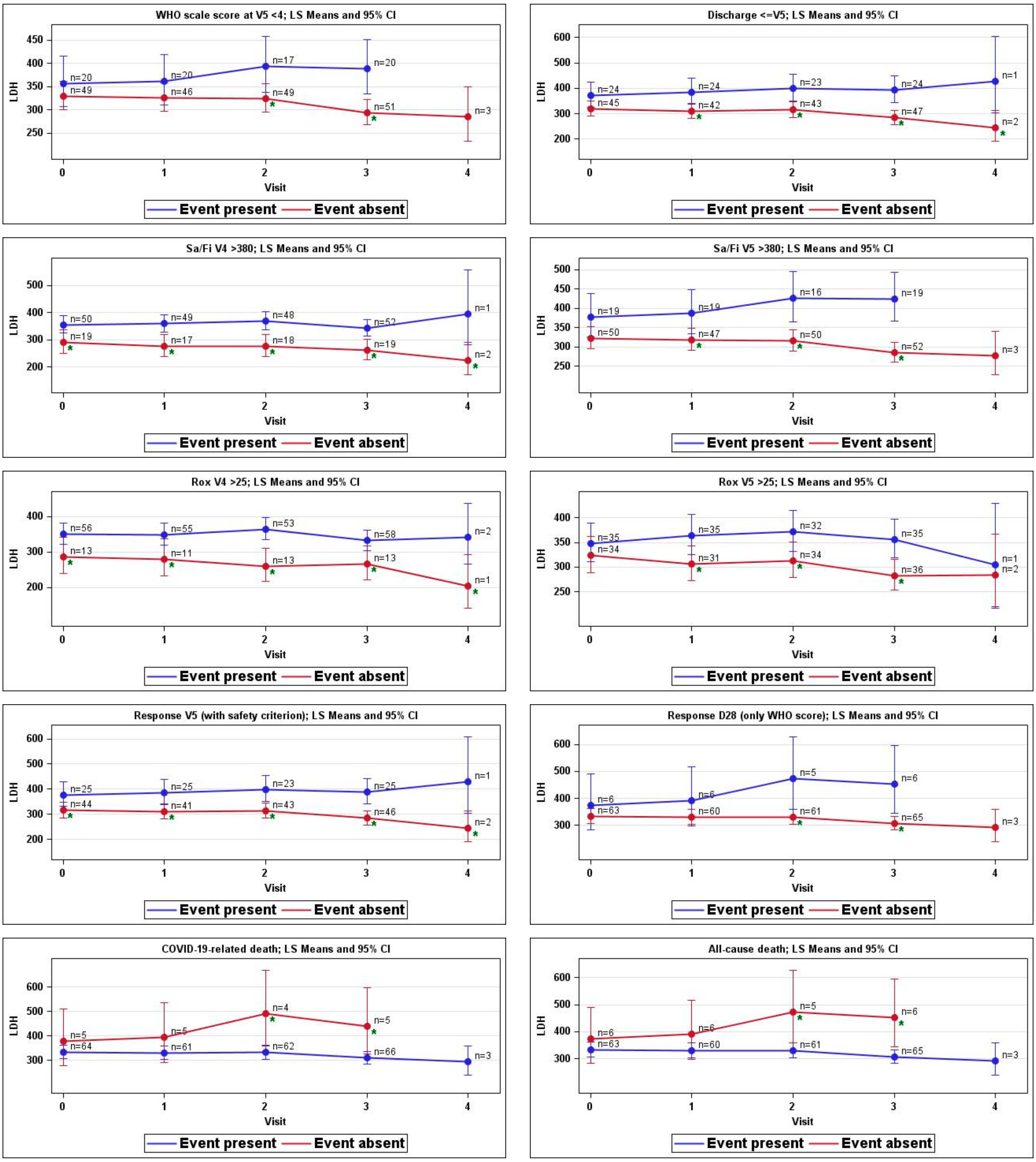

Abbreviations: LDH, lactate dehydrogenase. The units in the ordinate axis are international units per liter.

Figure S13: Adjusted means and inferences from the generalized linear mixed models of serum C reactive protein levels over the subgroups defined by dichotomic clinical milestones

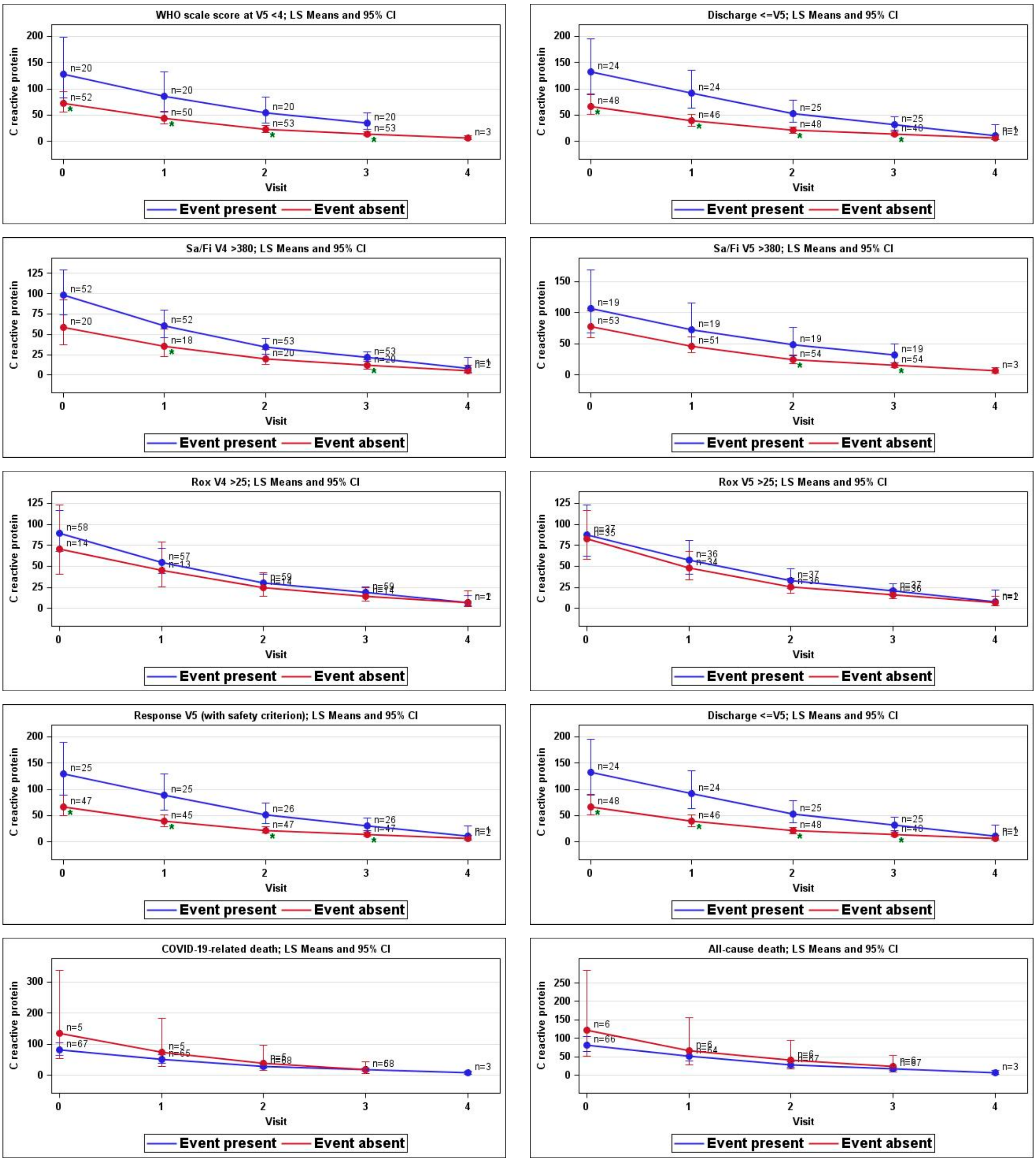

The units in the ordinate axis are milligrams per liter.

Figure S14: Adjusted means and inferences from the generalized linear mixed models of serum D dimer levels over the subgroups defined by dichotomic clinical milestones

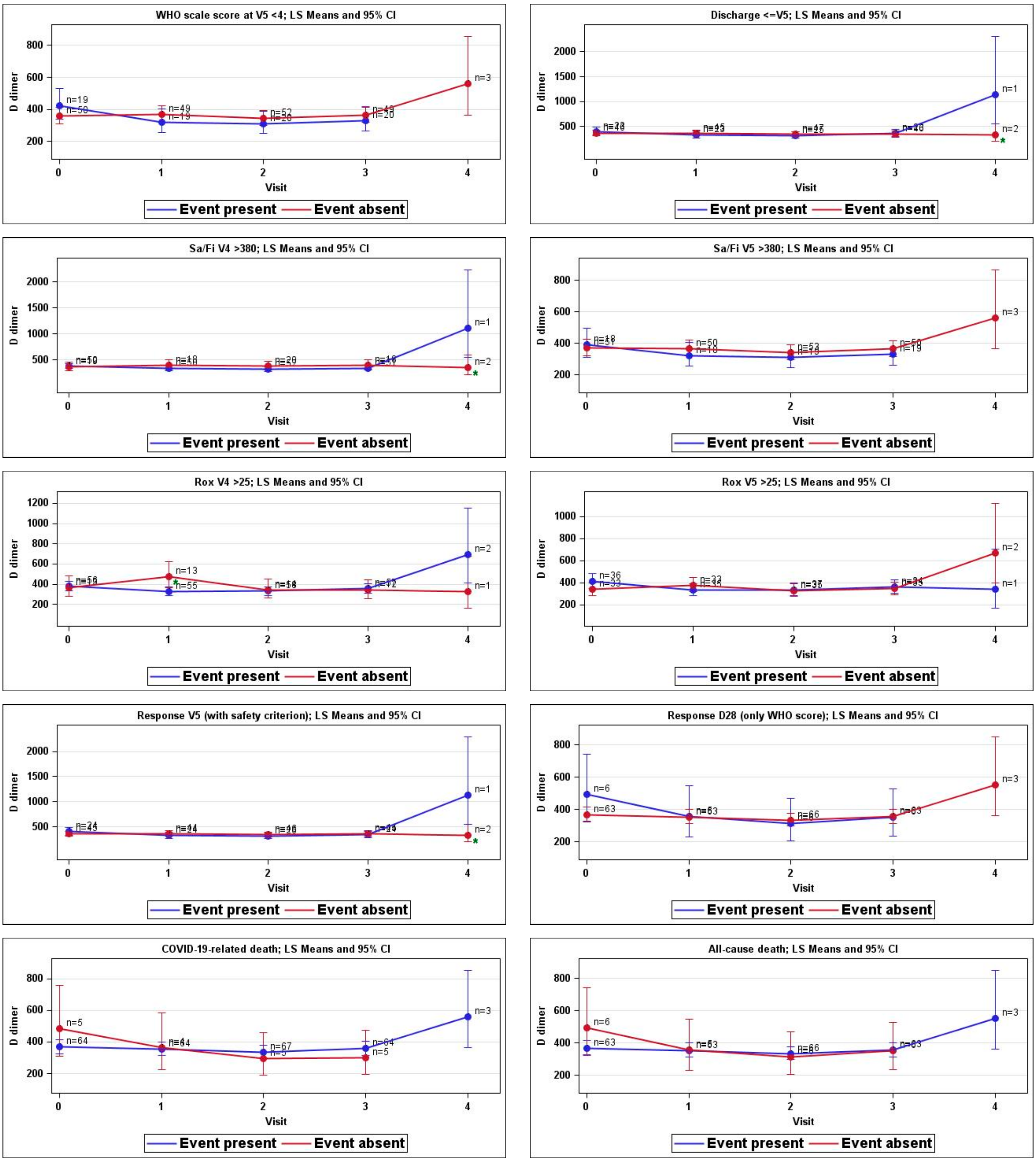

The units in the ordinate axis are micrograms per liter.

Figure S15: Adjusted means and inferences from the generalized linear mixed models of serum C1 esterase inhibitor levels (antigenic protein test) over the subgroups defined by dichotomic clinical milestones

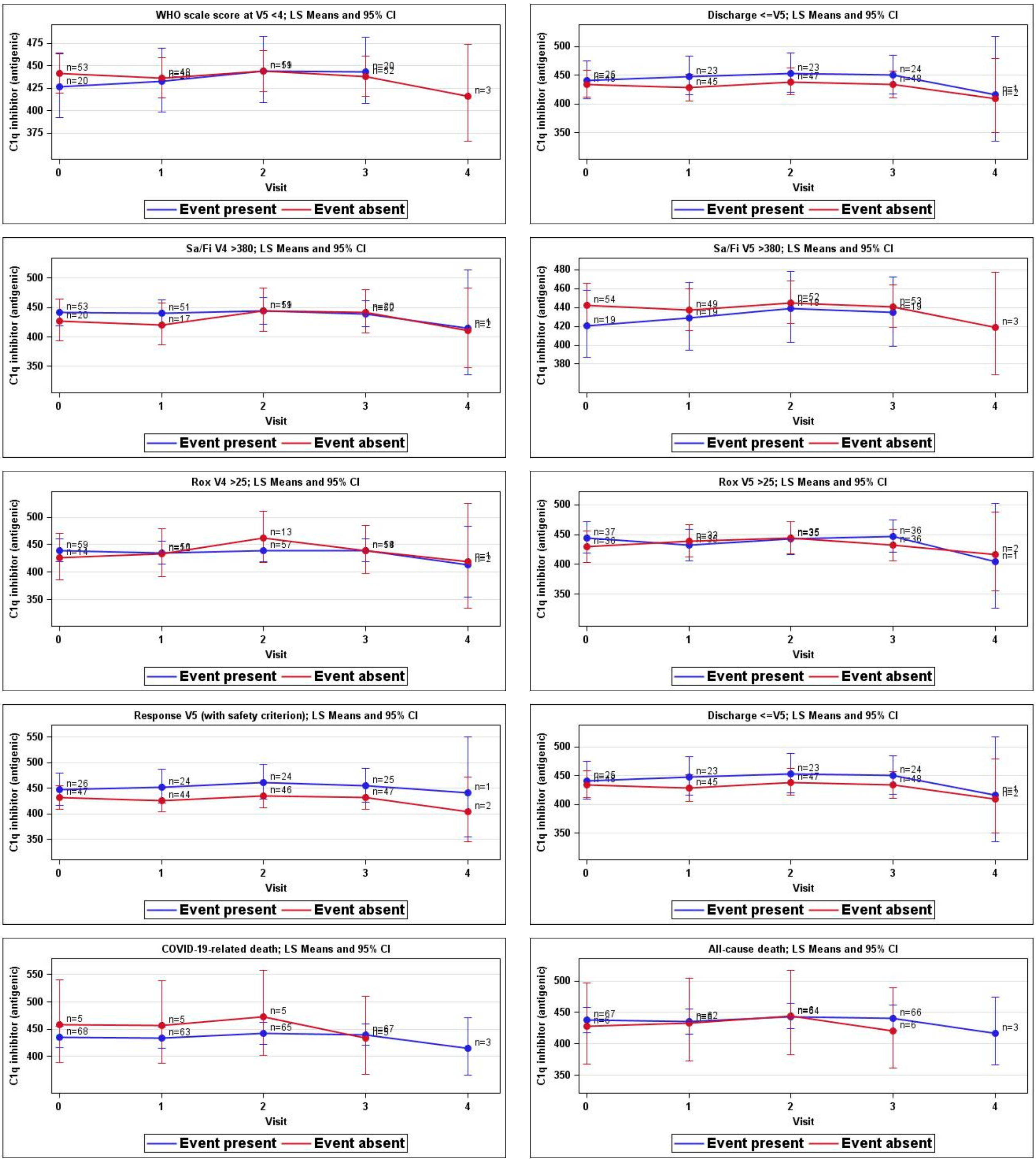

Abbreviations: C, complement. The units in the ordinate axis are milligrams per liter.

Figure S16: Adjusted means and inferences from the generalized linear mixed models of serum C1 esterase inhibitor activity (functional test) over the subgroups defined by dichotomic clinical milestones

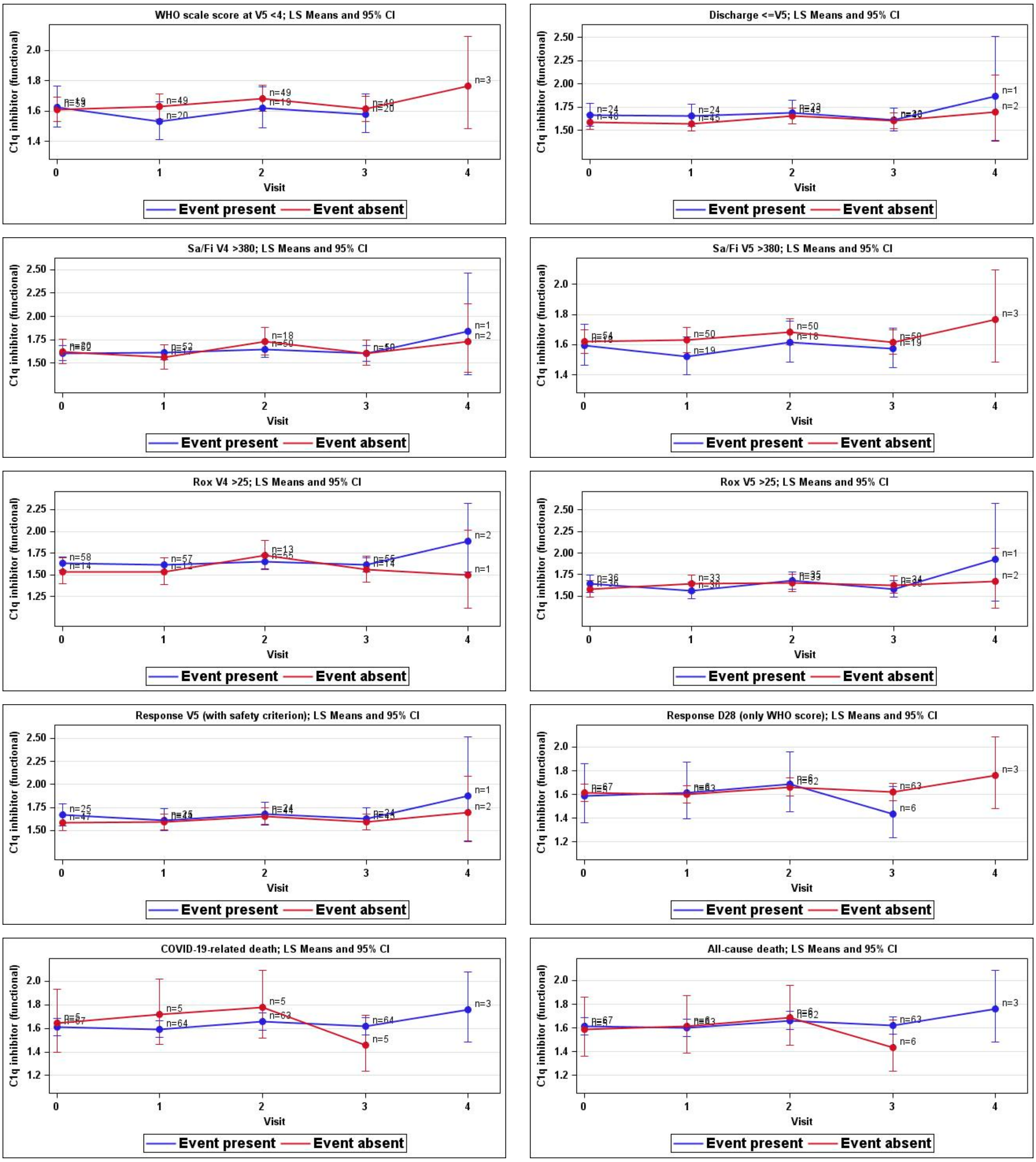

Abbreviations: C, complement. The units in the ordinate axis are unitless ratios.

Figure S17: Adjusted means and inferences from the generalized linear mixed models of serum C4 factor levels over the subgroups defined by dichotomic clinical milestones

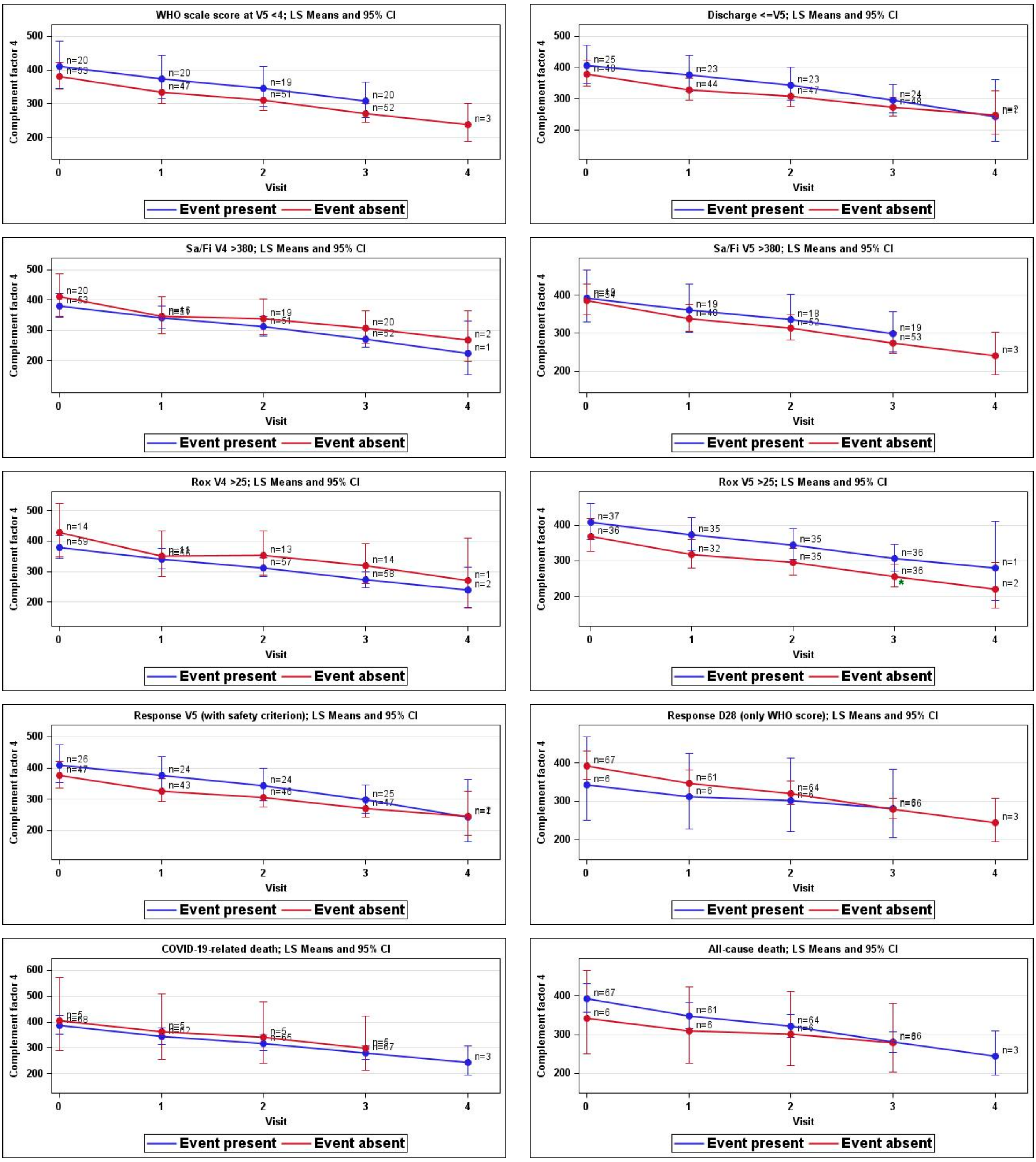

Abbreviations: C, complement. The units in the ordinate axis are milligrams per liter.

Figure S18: Adjusted means and inferences from the generalized linear mixed models of blood lymphocyte counts over the subgroups defined by dichotomic clinical milestones

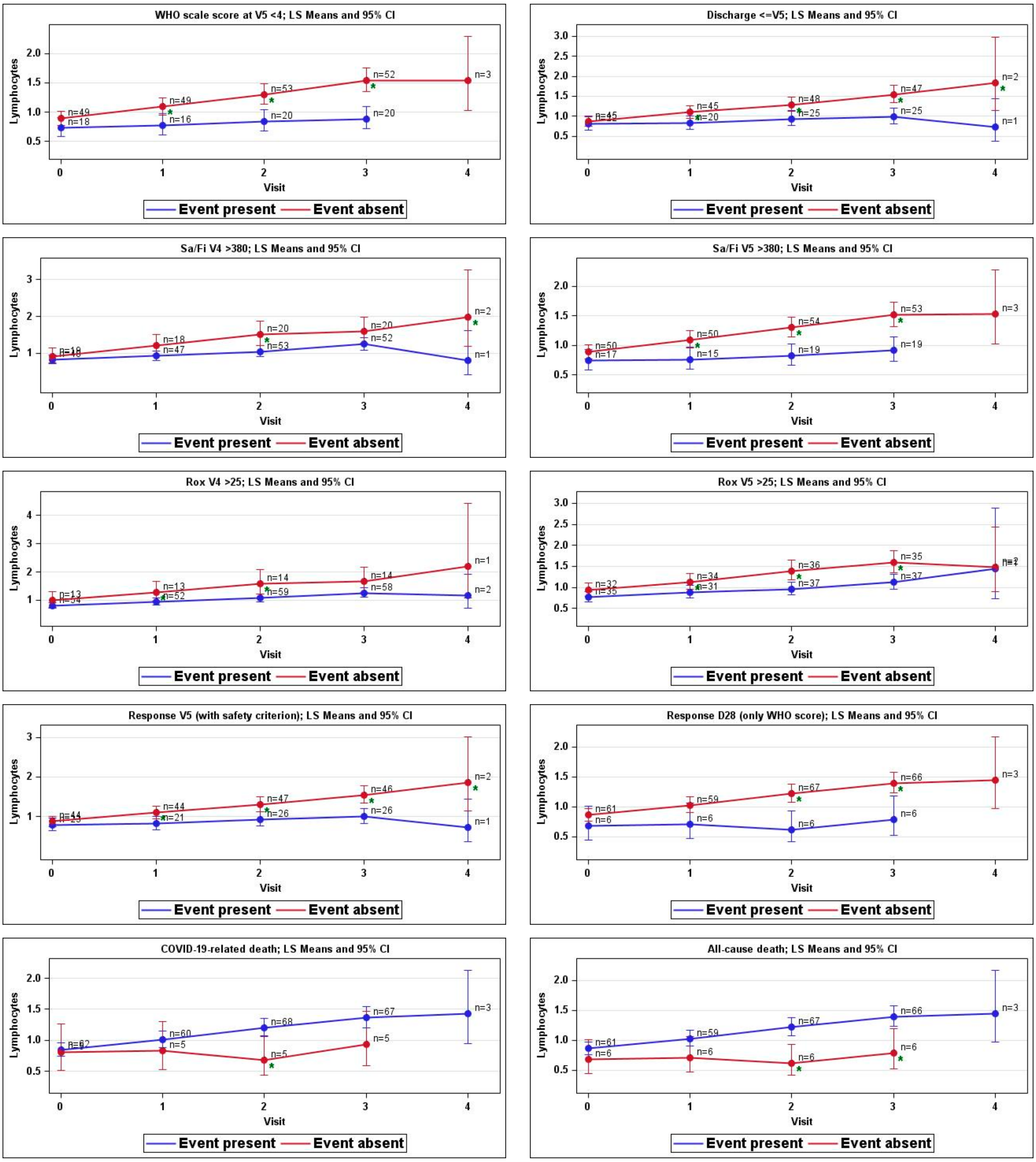

The units in the ordinate axis are counts per nanoliter. The slopes are multiplied by 10 for convenience in subsequent analyses.

Figure S19: Concurrent criterion-type validity of the discriminative ability of interleukin 6 to discern between patients who did and did not achieve a WHO status score scale <4 at Day 10 (Visit 5), both unadjusted (empirical ROC curve) and adjusted by either Icatibant treatment or the time elapsed from symptom onset to hospitalization (binormal induced smooth ROC curves)

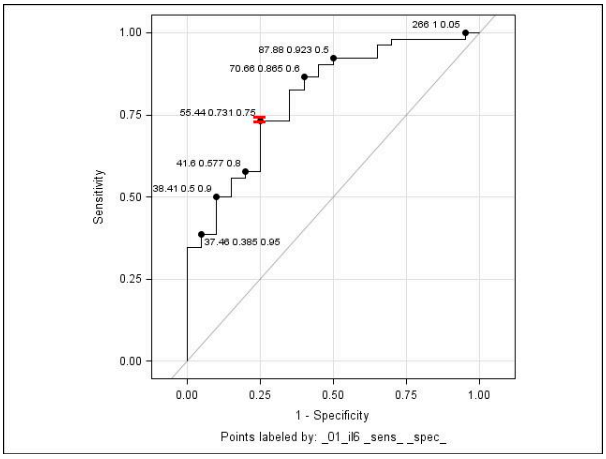

Levels of a representative patient on best cut-off score (baseline, Day 2, Day 3, Day 4):

28.2, 32.9, 55, 104.1 pg/mL

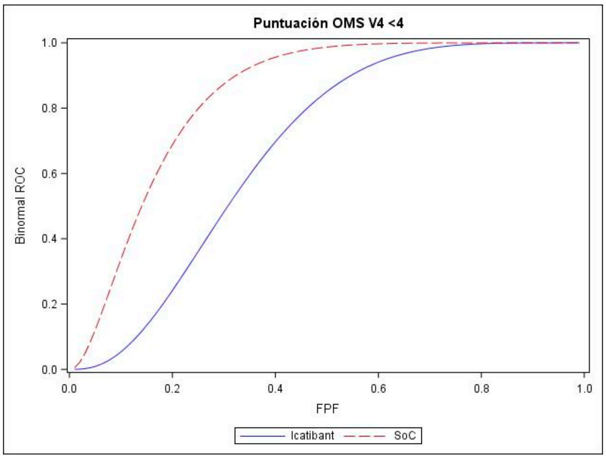

Icatibant treatment associates to a non-significant reduction (p=0.107) of the discriminative ability of IL-6, going from an AUC of 0.834 in the SoC group to AUC of 0.675 with Icatibant

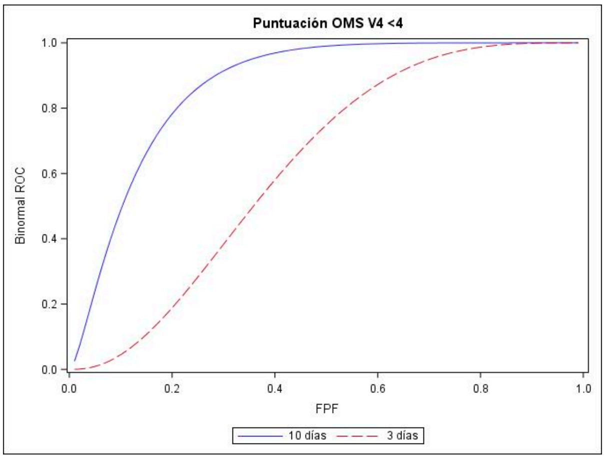

Time from symptom onset to hospitalization associates to an almost significant increase (p=0.067) of the discriminative ability of IL-6, going from an AUC of 0.625 after 3 days to AUC of 0.867 after 10 days

Abbreviations: AUC, area under the curve; IL, interleukin; mL, milliliter; pg, picogram; ROC, receiver operating characteristic; SoC, standard of care; WHO, World Health Organization.

Figure S20: Concurrent criterion-type validity of the discriminative ability of interleukin 6 to discern between patients who had and had not been discharged at Day 10 (Visit 5), both unadjusted (empirical ROC curve) and adjusted by either Icatibant treatment or the time elapsed from symptom onset to hospitalization (binormal induced smooth ROC curves)

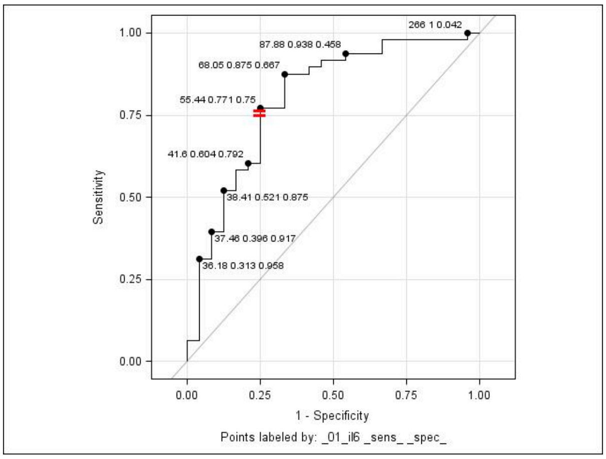

Levels of a representative patient on best cut-off score (baseline, Day 2, Day 3, Day 4):

28.2, 32.9, 55, 104.1 pg/mL

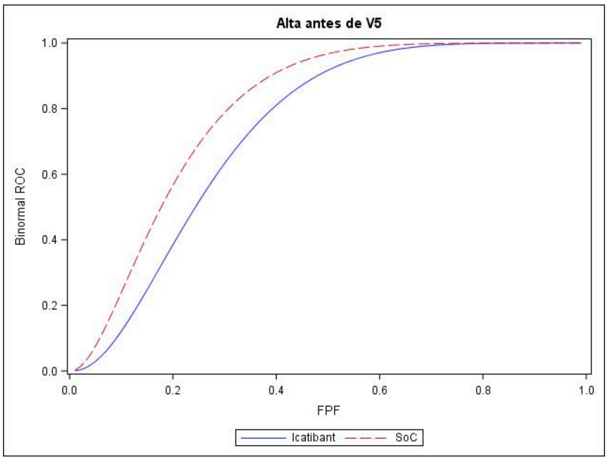

Icatibant treatment associates to a non-significant reduction (p=0.526) of the discriminative ability of IL-6, going from an AUC of 0.796 in the SoC group to AUC of 0.733 with Icatibant

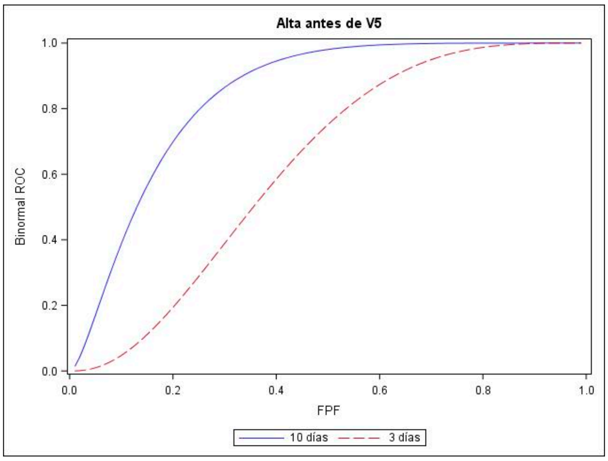

Time from symptom onset to hospitalization associates to non-significant increase (p=0.137) of the discriminative ability of IL-6, going from an AUC of 0.628 after 3 days to AUC of 0.839 after 10 days

Abbreviations: AUC, area under the curve; IL, interleukin; mL, milliliter; pg, picogram; ROC, receiver operating characteristic; SoC, standard of care.

Figure S21: Concurrent criterion-type validity of the discriminative ability of interleukin 6 to discern between patients who did and did not attain a median Sp_{O_2}/Fi_{O_2} ratio >380 at Day 4 (Visit 4), both unadjusted (empirical ROC curve) and adjusted by either Icatibant treatment or the time elapsed from symptom onset to hospitalization (binormal induced smooth ROC curves)

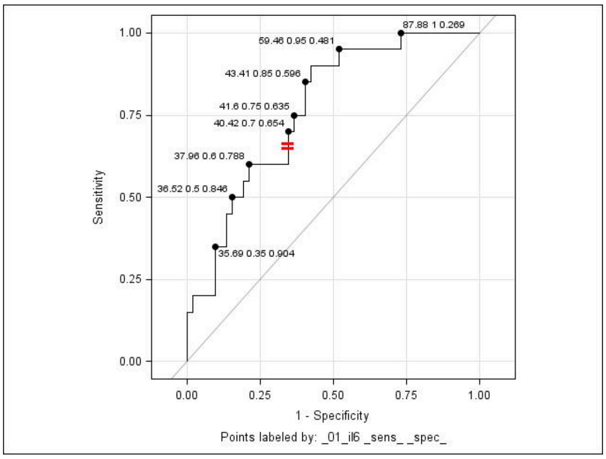

Levels of a representative patient on best cut-off score (baseline, Day 2, Day 3, Day 4):

16.3, 27, 38.2, 10.1 pg/mL

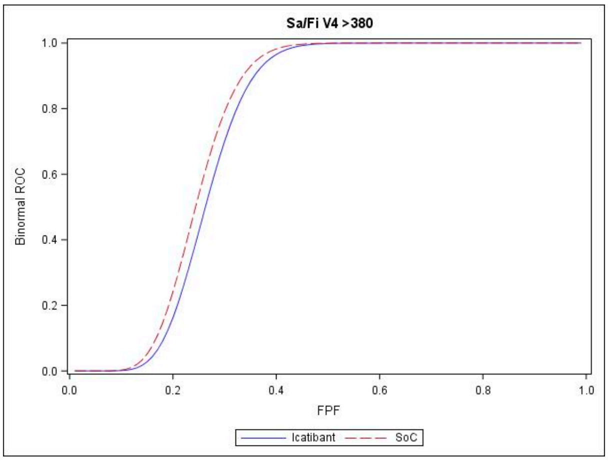

Icatibant treatment is not associated with apparent changes (p=0.896) of the discriminative ability of IL-6, going from an AUC of 0.752 in the SoC group to AUC of 0.733 with Icatibant

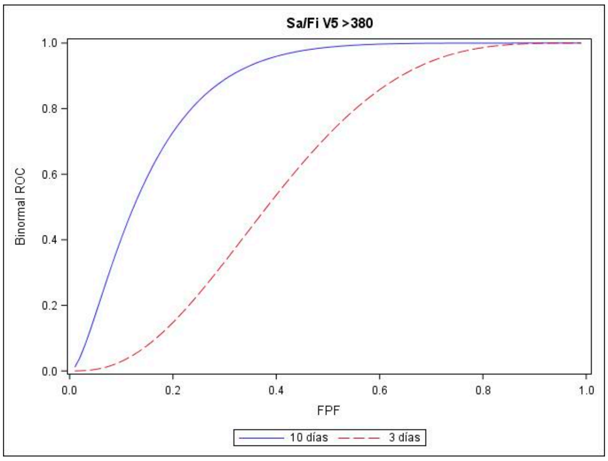

Time from symptom onset to hospitalization associates to non-significant increase (p=0.340) of the discriminative ability of IL-6, going from an AUC of 0.614 after 3 days to AUC of 0.813 after 10 days

Abbreviations: AUC, area under the curve; IL, interleukin; mL, milliliter; pg, picogram; ROC, receiver operating characteristic; SoC, standard of care.

Figure S22: Concurrent criterion-type validity of the discriminative ability of interleukin 6 to discern between patients who did and did not attain a median Sp_{O_2}/Fi_{O_2} ratio >380 at Day 10 (Visit 5), both unadjusted (empirical ROC curve) and adjusted by either Icatibant treatment or the time elapsed from symptom onset to hospitalization (binormal induced smooth ROC curves)

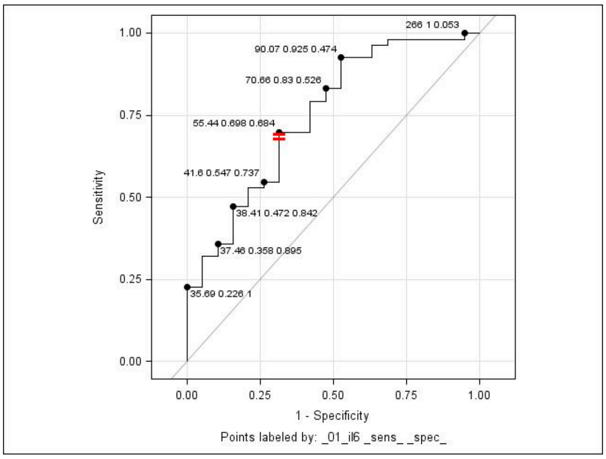

Levels of a representative patient on best cut-off score (baseline, Day 2, Day 3, Day 4):

28.2, 32.9, 55, 104.1 pg/mL

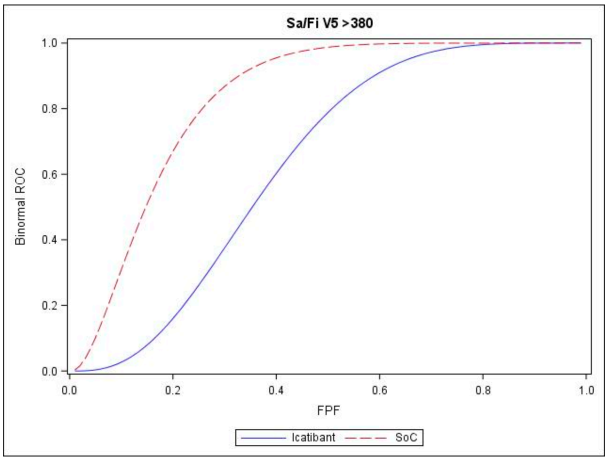

Icatibant treatment associates to an almost significant reduction (p=0.057) of the discriminative ability of IL-6, going from an AUC of 0.829 in the SoC group to AUC of 0.633 with Icatibant

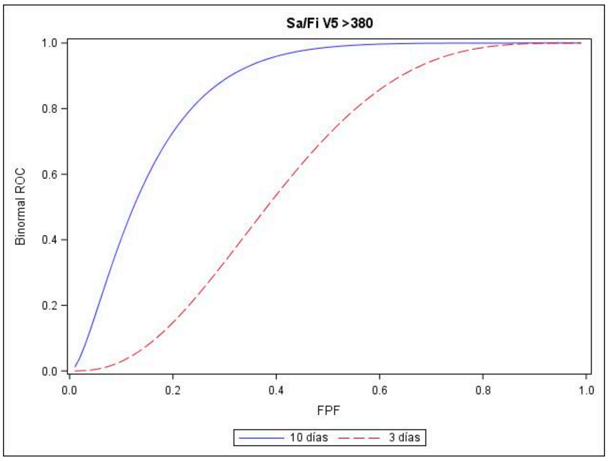

Time from symptom onset to hospitalization associates to an almost significant increase (p=0.074) of the discriminative ability of IL-6, going from an AUC of 0.605 after 3 days to AUC of 0.848 after 10 days

Abbreviations: AUC, area under the curve; IL, interleukin; mL, milliliter; pg, picogram; ROC, receiver operating characteristic; SoC, standard of care.

Figure S23: Concurrent criterion-type validity of the discriminative ability of interleukin 6 to discern between patients who did and did not attain a median Rox index >25 at Day 4 (Visit 4), both unadjusted (empirical ROC curve) and adjusted by either Icatibant treatment or the time elapsed from symptom onset to hospitalization (binormal induced smooth ROC curves)

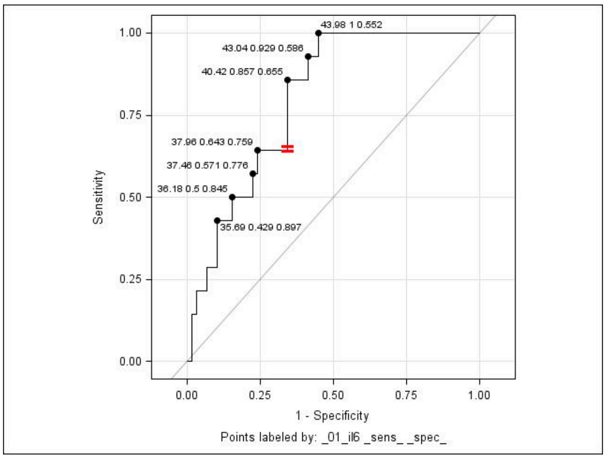

Levels of a representative patient on best cut-off score (baseline, Day 2, Day 3, Day 4):

16.3, 27, 38.2, 10.1 pg/mL

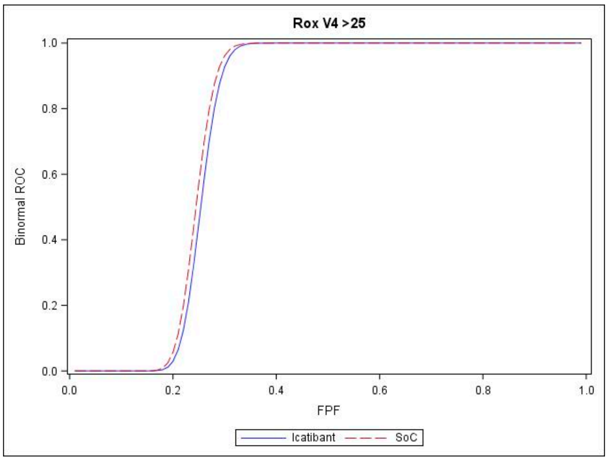

Icatibant treatment is not associated with apparent changes (p=0.957) of the discriminative ability of IL-6, going from an AUC of 0.754 in the SoC group to AUC of 0.745 with Icatibant

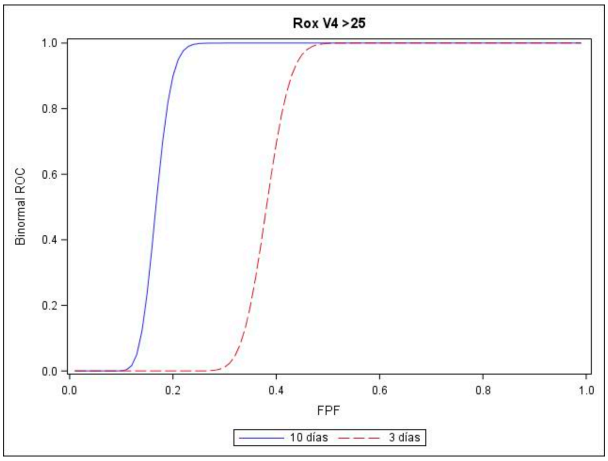

Time from symptom onset to hospitalization associates to a non-significant increase (p=0.400) of the discriminative ability of IL-6, going from an AUC of 0.619 after 3 days to AUC of 0.832 after 10 days

Abbreviations: AUC, area under the curve; IL, interleukin; mL, milliliter; pg, picogram; ROC, receiver operating characteristic; SoC, standard of care.

Figure S24: Concurrent criterion-type validity of the discriminative ability of interleukin 6 to discern between patients who did and did not attain a median Rox index >25 at Day 10 (Visit 5), both unadjusted (empirical ROC curve) and adjusted by either Icatibant treatment or the time elapsed from symptom onset to hospitalization (binormal induced smooth ROC curves)

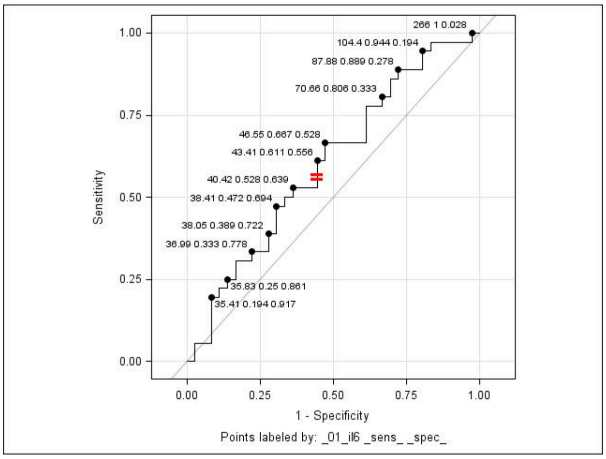

Levels of a representative patient on best cut-off score (baseline, Day 2, Day 3, Day 4):

19, 12.8, 27.9, 43.2 pg/mL

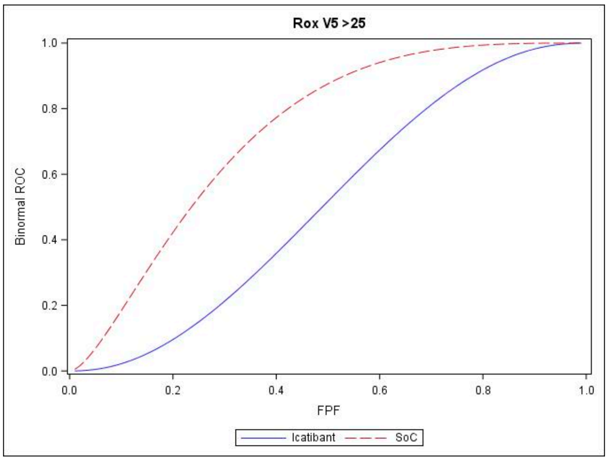

Icatibant treatment associates to an almost significant reduction (p=0.081) of the discriminative ability of IL-6, going from an AUC of 0.729 in the SoC group to AUC of 0.509 with Icatibant

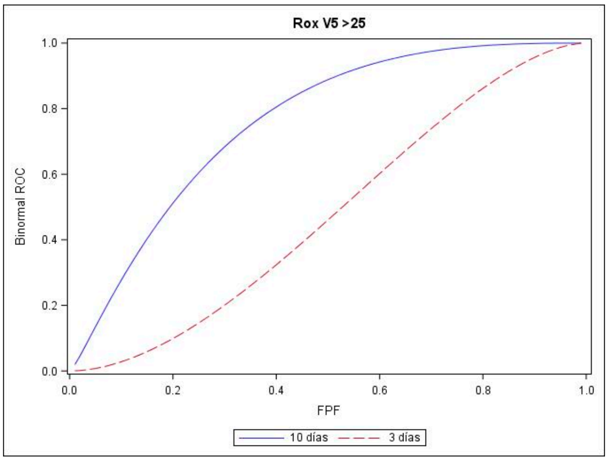

Time from symptom onset to hospitalization associates to a non-significant increase (p=0.111) of the discriminative ability of IL-6, going from an AUC of 0.477 after 3 days to AUC of 0.759 after 10 days

Abbreviations: AUC, area under the curve; IL, interleukin; mL, milliliter; pg, picogram; ROC, receiver operating characteristic; SoC, standard of care.

Figure S25: Concurrent criterion-type validity of the discriminative ability of interleukin 6 to discern between patients who did and did not attain a clinical response at Day 10 (Visit 5), both unadjusted (empirical ROC curve) and adjusted by either Icatibant treatment or the time elapsed from symptom onset to hospitalization (binormal induced smooth ROC curves)

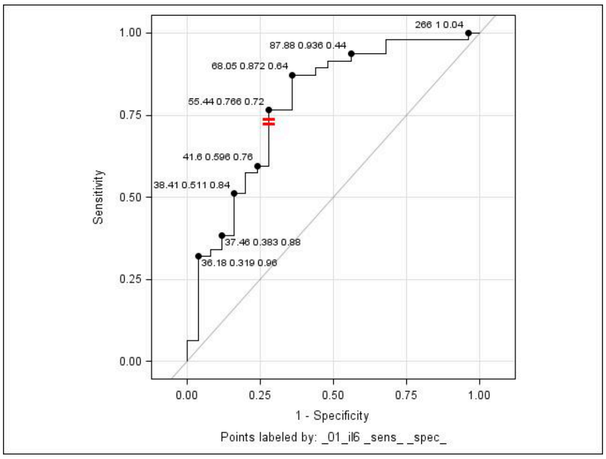

Levels of a representative patient on best cut-off score (baseline, Day 2, Day 3, Day 4):

28.2, 32.9, 55, 104.1 pg/mL

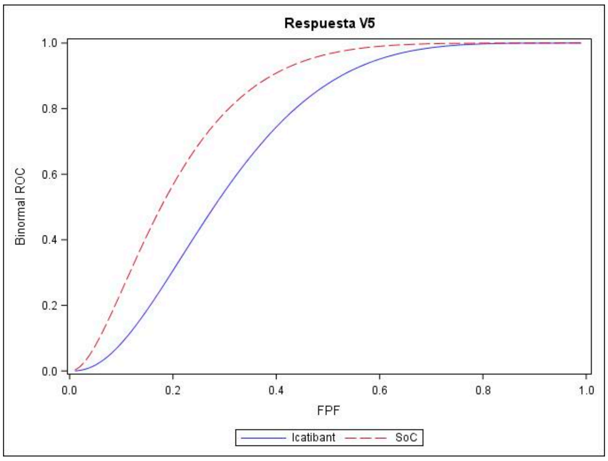

Icatibant treatment associates to a non-significant reduction (p=0.343) of the discriminative ability of IL-6, going from an AUC of 0.796 in the SoC group to AUC of 0.699 with Icatibant

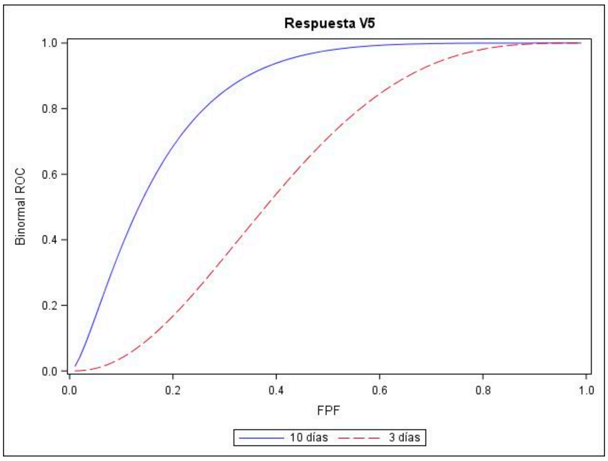

Time from symptom onset to hospitalization associates to a non-significant increase (p=0.120) of the discriminative ability of IL-6, going from an AUC of 0.606 after 3 days to AUC of 0.834 after 10 days

Abbreviations: AUC, area under the curve; IL, interleukin; mL, milliliter; pg, picogram; ROC, receiver operating characteristic; SoC, standard of care.

Figure S26: Concurrent criterion-type validity of the discriminative ability of interleukin 6 to discern between patients who did and did not attain a clinical response 28 days after initial discharge, both unadjusted (empirical ROC curve) and adjusted by either Icatibant treatment or the time elapsed from symptom onset to hospitalization (binormal induced smooth ROC curves)

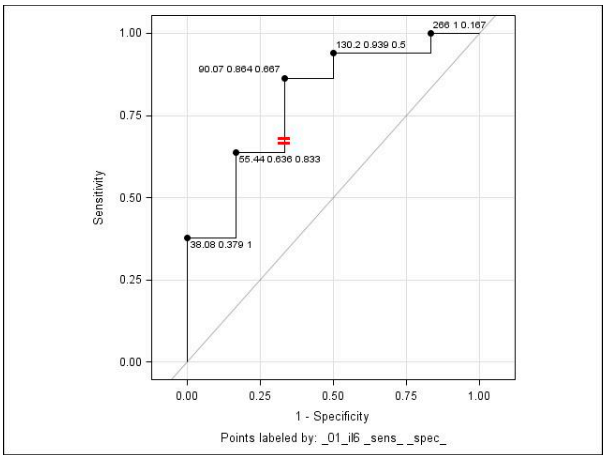

Levels of a representative patient on best cut-off score (baseline, Day 2, Day 3, Day 4):

28.2, 32.9, 55, 104.1 pg/mL

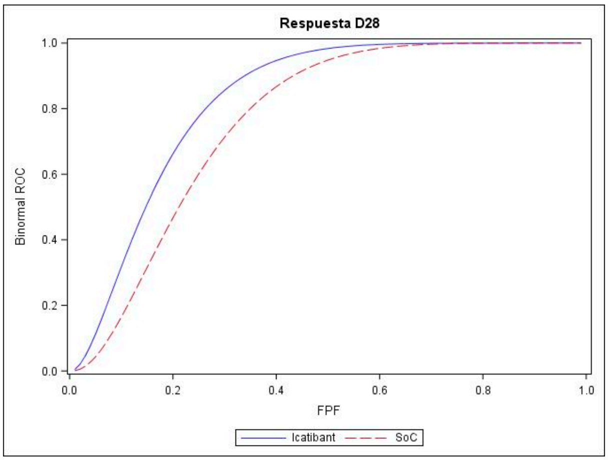

Icatibant treatment associates to a non-significant increase (Bayes) of the discriminative ability of IL-6, going from an AUC of 0.764 in the SoC group to AUC of 0.824 with Icatibant

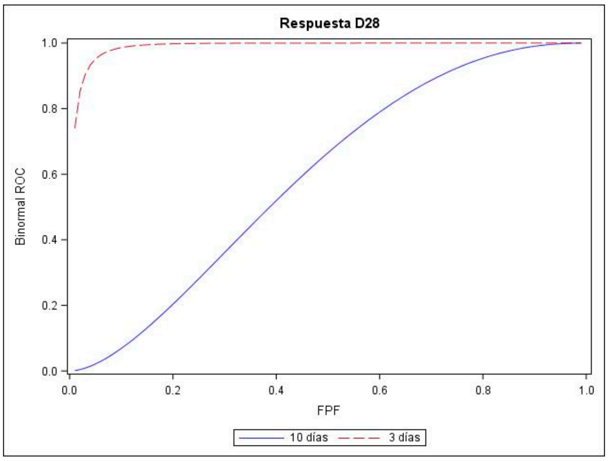

Time from symptom onset to hospitalization associates to a significant reduction (p=0.007) of the discriminative ability of IL-6, going from an AUC of 0.989 after 3 days to AUC of 0.594 after 10 days

Abbreviations: AUC, area under the curve; IL, interleukin; mL, milliliter; pg, picogram; ROC, receiver operating characteristic; SoC, standard of care.

Figure S27: Concurrent criterion-type validity of the discriminative ability of interleukin 6 to discern patients who died from COVID-19, both unadjusted (empirical ROC curve) and adjusted by either Icatibant treatment or the time elapsed from symptom onset to hospitalization (binormal induced smooth ROC curves)

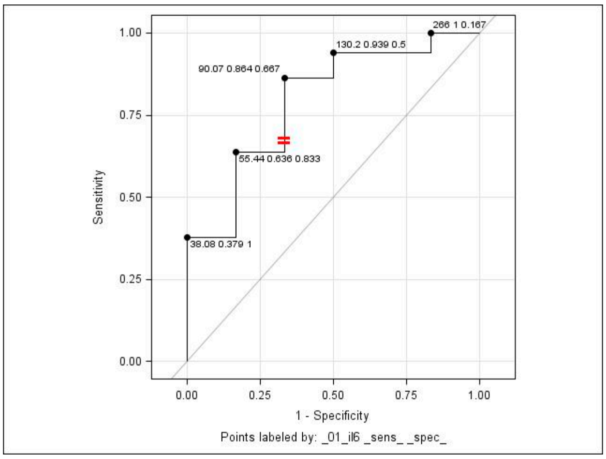

Levels of a representative patient on best cut-off score (baseline, Day 2, Day 3, Day 4):

28.2, 32.9, 55, 104.1 pg/mL

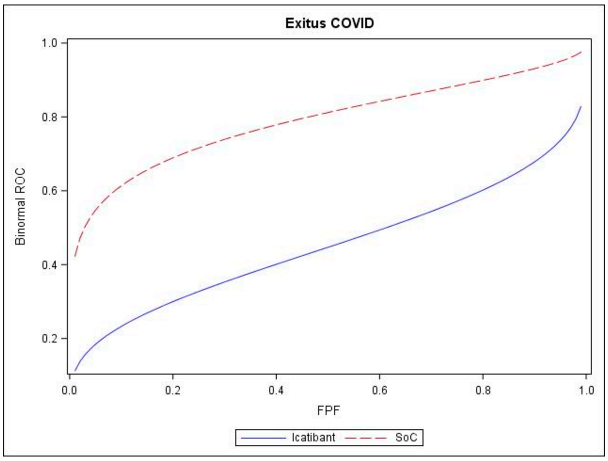

Icatibant treatment associates to a non-significant reduction (Bayes) of the discriminative ability of IL-6, going from an AUC of 0.789 in the SoC group to AUC of 0.452 with Icatibant

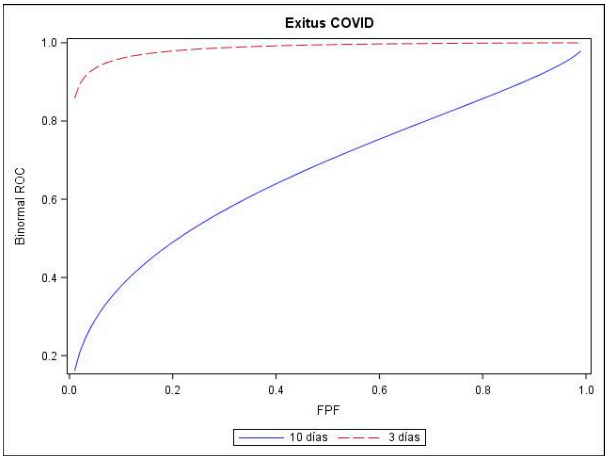

Time from symptom onset to hospitalization associates to a significant reduction (p=0.025) of the discriminative ability of IL-6, going from an AUC of 0.985 after 3 days to AUC of 0.669 after 10 days

Abbreviations: AUC, area under the curve; IL, interleukin; mL, milliliter; pg, picogram; ROC, receiver operating characteristic; SoC, standard of care.

Figure S28: Concurrent criterion-type validity of the discriminative ability of interleukin 6 to discern patients who died from any reason, both unadjusted (empirical ROC curve) and adjusted by either Icatibant treatment or the time elapsed from symptom onset to hospitalization (binormal induced smooth ROC curves)

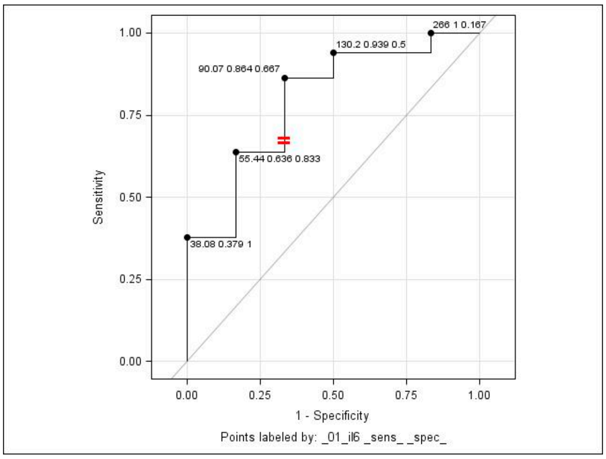

Levels of a representative patient on best cut-off score (baseline, Day 2, Day 3, Day 4):

28.2, 32.9, 55, 104.1 pg/mL

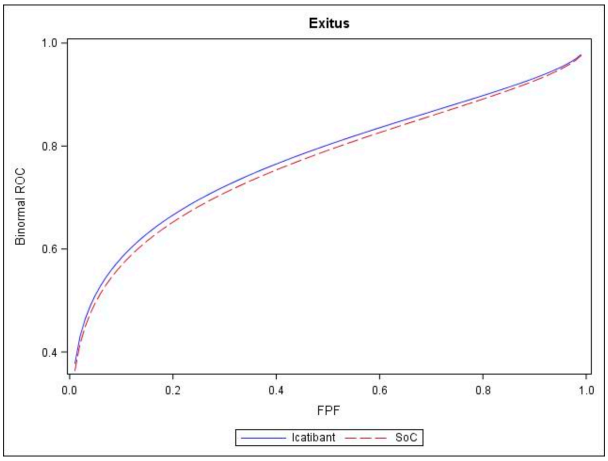

Icatibant treatment is not associated with apparent changes (Bayes) of the discriminative ability of IL-6, going from an AUC of 0.766 in the SoC group to AUC of 0.776 with Icatibant

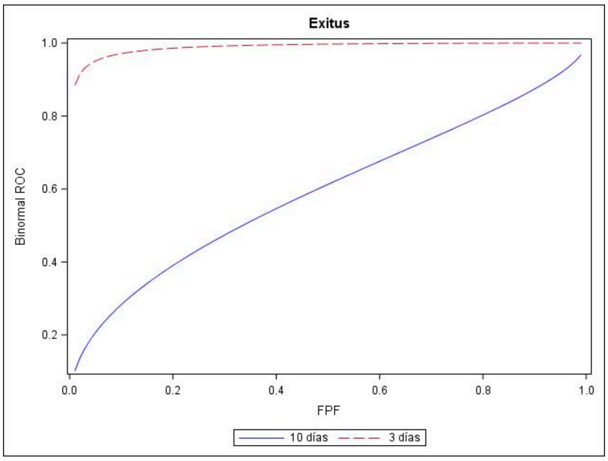

Time from symptom onset to hospitalization associates to a significant reduction (p=0.007) of the discriminative ability of IL-6, going from an AUC of 0.989 after 3 days to AUC of 0.594 after 10 days

Abbreviations: AUC, area under the curve; IL, interleukin; mL, milliliter; pg, picogram; ROC, receiver operating characteristic; SoC, standard of care.

Figure S29: Concurrent criterion-type validity of the discriminative ability of ferritin to discern between patients who did and did not attain a median Sp_{O_2}/Fi_{O_2} ratio >380 at Day 4 (Visit 4), both unadjusted (empirical ROC curve) and adjusted by either Icatibant treatment or the time elapsed from symptom onset to hospitalization (binormal induced smooth ROC curves)

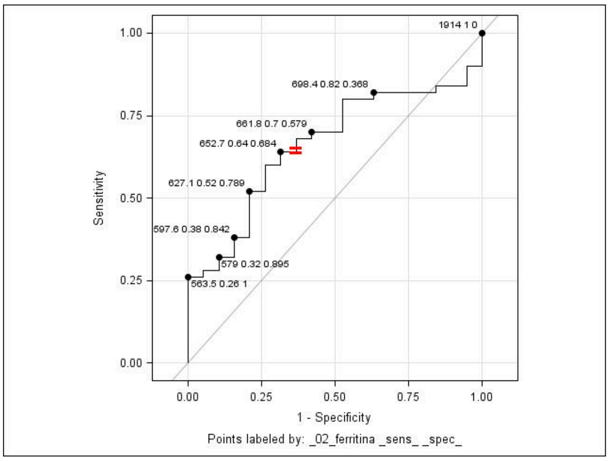

Levels of a representative patient on best cut-off score (baseline, Day 2, Day 3, Day 4):

743, 715.3, 769.8, 790.2 ng/mL

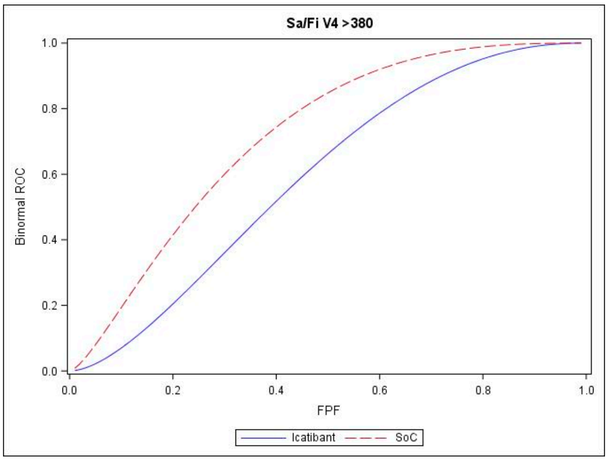

Icatibant treatment associates to non-significant reduction (p=0.420) of the discriminative ability of ferritin, going from an AUC of 0.718 in the SoC group to AUC of 0.593 with Icatibant

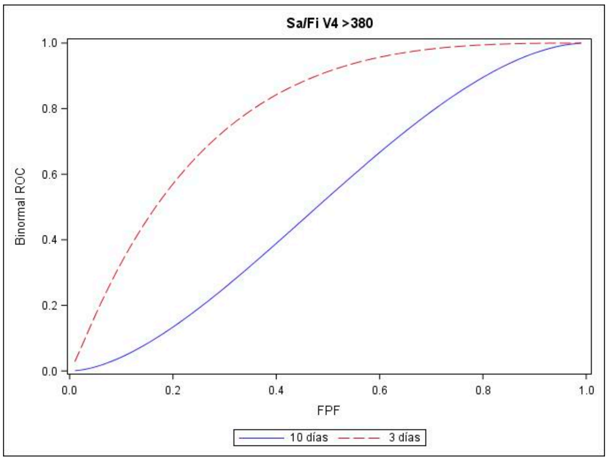

Time from symptom onset to hospitalization associates to non-significant reduction (p=0.194) of the discriminative ability of ferritin, going from an AUC of 0.785 after 3 days to AUC of 0.517 after 10 days

Abbreviations: AUC, area under the curve; mL, milliliter; ng, nanogram; ROC, receiver operating characteristic; SoC, standard of care.

Figure S30: Concurrent criterion-type validity of the discriminative ability of ferritin to discern between patients who did and did not attain a median Rox index >25 at Day 4 (Visit 4), both unadjusted (empirical ROC curve) and adjusted by either Icatibant treatment or the time elapsed from symptom onset to hospitalization (binormal induced smooth ROC curves)

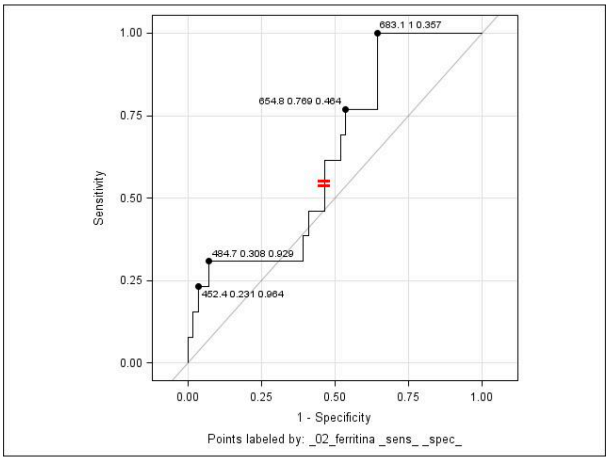

Levels of a representative patient on best cut-off score (baseline, Day 2, Day 3, Day 4):

84.3, 86.2, 79.4, 91.6 ng/mL

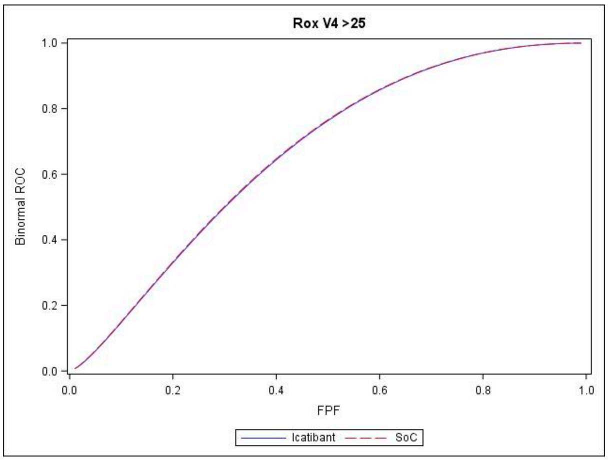

Icatibant treatment is not associated with apparent changes (p=0.992) of the discriminative ability of ferritin, going from an AUC of 0.655 in the SoC group to AUC of 0.664 with Icatibant

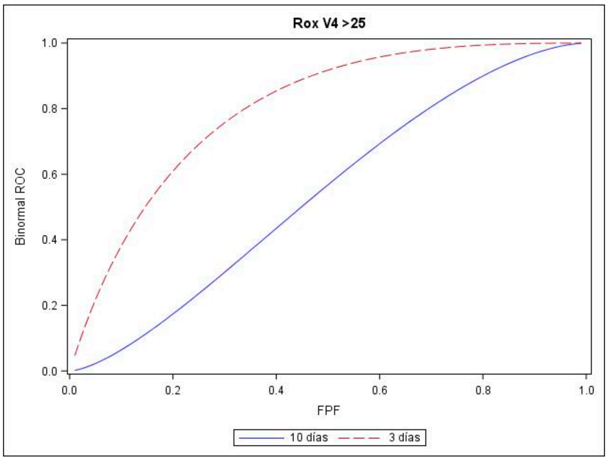

Time from symptom onset to hospitalization associates to non-significant reduction (p=0.286) of the discriminative ability of ferritin, going from an AUC of 0.799 after 3 days to AUC of 0.541 after 10 days

Abbreviations: AUC, area under the curve; mL, milliliter; ng, nanogram; ROC, receiver operating characteristic; SoC, standard of care.

Figure S31: Concurrent criterion-type validity of the discriminative ability of C reactive protein to discern between patients who did and did not attain a median Sp_{O_2}/Fi_{O_2} ratio >380 at Day 4 (Visit 4), both unadjusted (empirical ROC curve) and adjusted by either Icatibant treatment or the time elapsed from symptom onset to hospitalization (binormal induced smooth ROC curves)

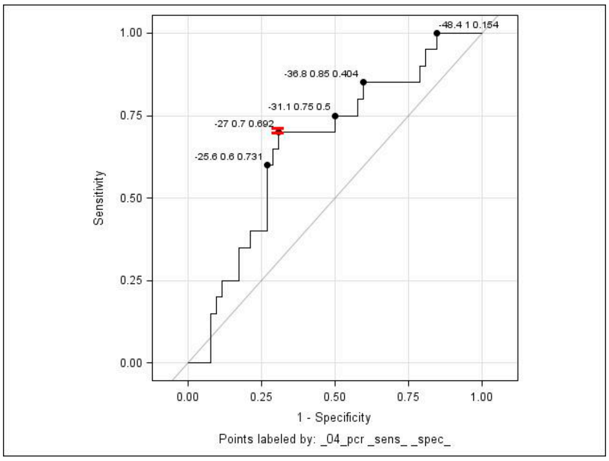

Levels of a representative patient on best cut-off score (baseline, Day 2, Day 3, Day 4):

31.8, 16.7, 7.9, 5 mg/L

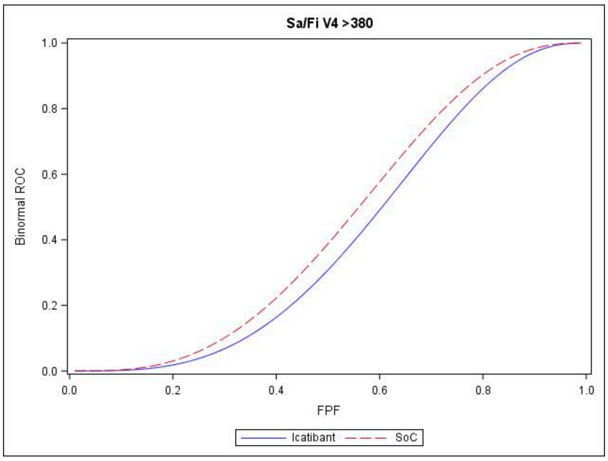

Icatibant treatment is not associated with apparent changes (p=0.810) of the discriminative ability of C reactive protein, going from an AUC of 0.447 in the SoC group to AUC of 0.407 with Icatibant

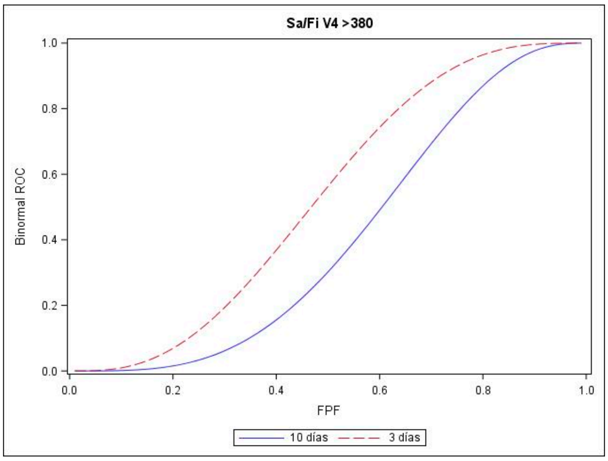

Time from symptom onset to hospitalization associates to non-significant reduction (p=0.605) of the discriminative ability of C reactive protein, going from an AUC of 0.529 after 3 days to AUC of 0.407 after 10 days

Abbreviations: AUC, area under the curve; L, liter; mg, milligram; ROC, receiver operating characteristic; SoC, standard of care.

Figure S32: Concurrent criterion-type validity of the discriminative ability of C reactive protein to discern between patients who did and did not attain a median Rox index >25 at Day 4 (Visit 4), both unadjusted (empirical ROC curve) and adjusted by either Icatibant treatment or the time elapsed from symptom onset to hospitalization (binormal induced smooth ROC curves)

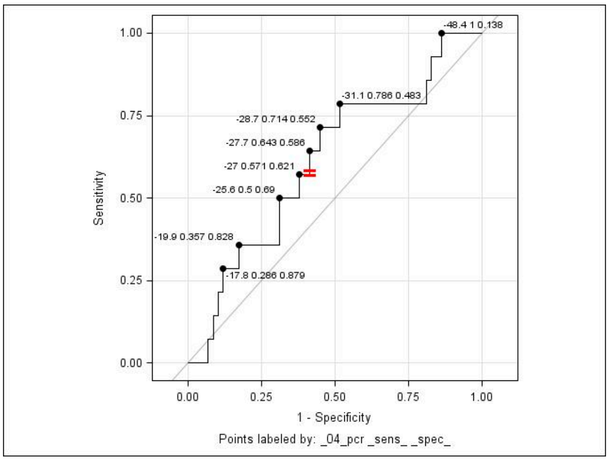

Levels of a representative patient on best cut-off score (baseline, Day 2, Day 3, Day 4):

57.9, 35.9, 32.6, 12 mg/L

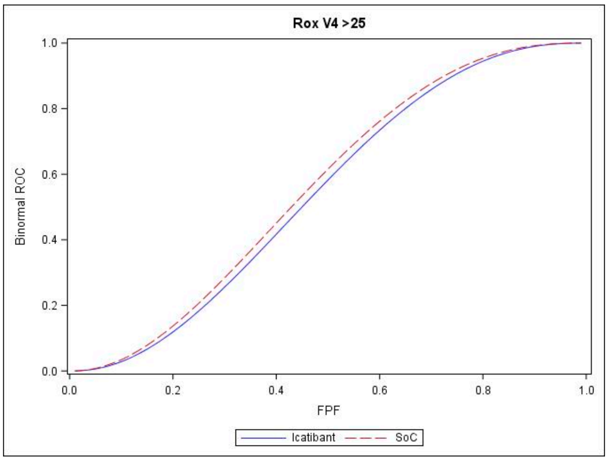

Icatibant treatment is not associated with apparent changes (p=0.926) of the discriminative ability of C reactive protein, going from an AUC of 0.561 in the SoC group to AUC of 0.543 with Icatibant

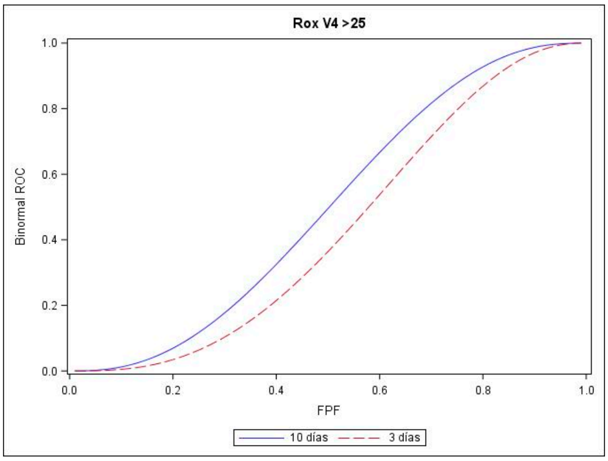

Time from symptom onset to hospitalization is not associated with apparent changes (p=0.810) of the discriminative ability of C reactive protein, going from an AUC of 0.431 after 3 days to AUC of 0.498 after 10 days

Abbreviations: AUC, area under the curve; L, liter; mg, milligram; ROC, receiver operating characteristic; SoC, standard of care.

Figure S33: Concurrent criterion-type validity of the discriminative ability of C reactive protein to discern between patients who did and did not attain a median Rox index >25 at Day 10 (Visit 5), both unadjusted (empirical ROC curve) and adjusted by either Icatibant treatment or the time elapsed from symptom onset to hospitalization (binormal induced smooth ROC curves)

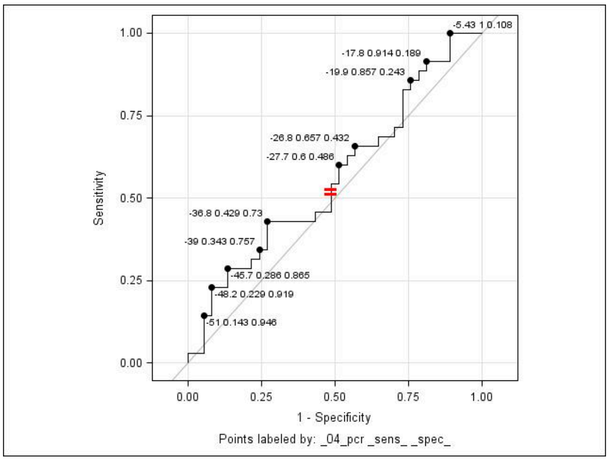

Levels of a representative patient on best cut-off score (baseline, Day 2, Day 3, Day 4):

136.9, 109.7, 92.4, 35.1 mg/L

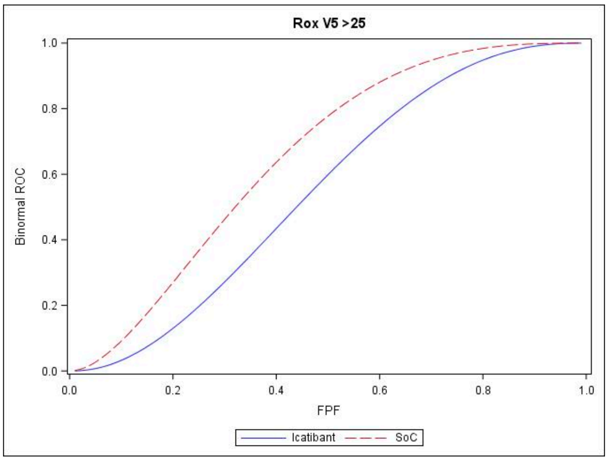

Icatibant treatment associates to a non-significant reduction (p=0.429) of the discriminative ability of C reactive protein, going from an AUC of 0.655 in the SoC group to AUC of 0.552 with Icatibant

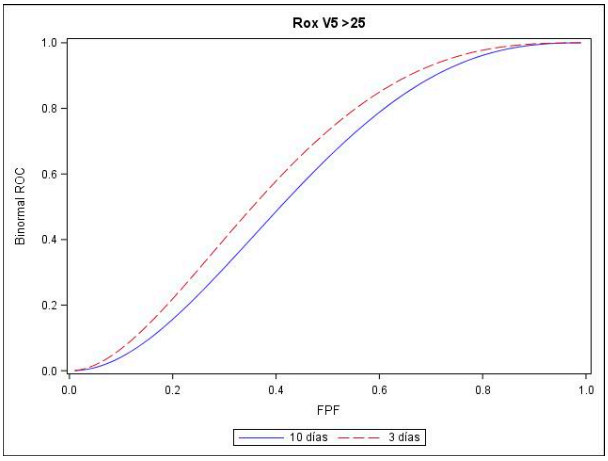

Time from symptom onset to hospitalization associates to a non-significant reduction (p=0.802) of the discriminative ability of C reactive protein, going from an AUC of 0.625 after 3 days to AUC of 0.578 after 10 days

Abbreviations: AUC, area under the curve; L, liter; mg, milligram; ROC, receiver operating characteristic; SoC, standard of care.

Figure S34: Concurrent criterion-type validity of the discriminative ability of D dimer to discern between patients who did and did not attain a median Sp_{O_2}/Fi_{O_2} ratio >380 at Day 4 (Visit 4), both unadjusted (empirical ROC curve) and adjusted by either Icatibant treatment or the time elapsed from symptom onset to hospitalization (binormal induced smooth ROC curves)

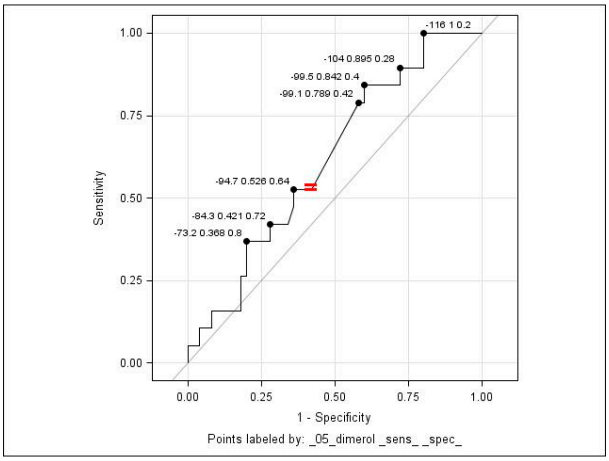

Levels of a representative patient on best cut-off score (baseline, Day 2, Day 3, Day 4):

250, 277, 250, 250 µg/L

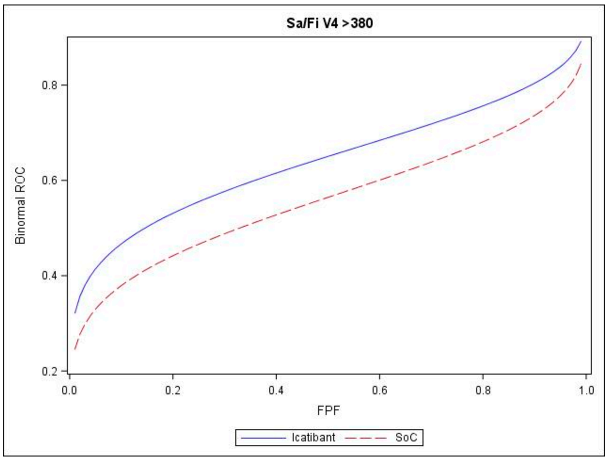

Icatibant treatment associates to a non-significant increase (p=0.500) of the discriminative ability of D dimer, going from an AUC of 0.561 in the SoC group to AUC of 0.642 with Icatibant

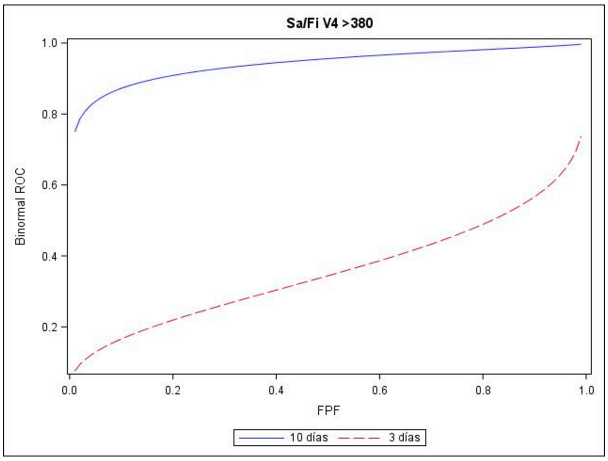

Time from symptom onset to hospitalization associates to a significant increase (p<0.001) of the discriminative ability of D dimer, going from an AUC of 0.357 after 3 days to AUC of 0.941 after 10 days

Abbreviations: AUC, area under the curve; L, liter; µg, microgram; ROC, receiver operating characteristic; SoC, standard of care.

Figure S35: Concurrent criterion-type validity of the discriminative ability of C1 esterase inhibitor (antigenic protein test) between patients who did and did not attain a median Sp_{O_2}/Fi_{O_2} ratio >380 at Day 4 (Visit 4), both unadjusted (empirical ROC curve) and adjusted by either Icatibant treatment or the time elapsed from symptom onset to hospitalization (binormal induced smooth ROC curves)

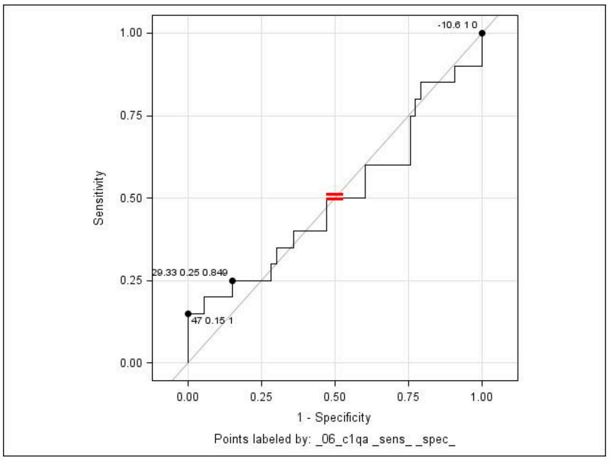

Levels of a representative patient on best cut-off score (baseline, Day 2, Day 3, Day 4):

292, 320, 292, 374 mg/L

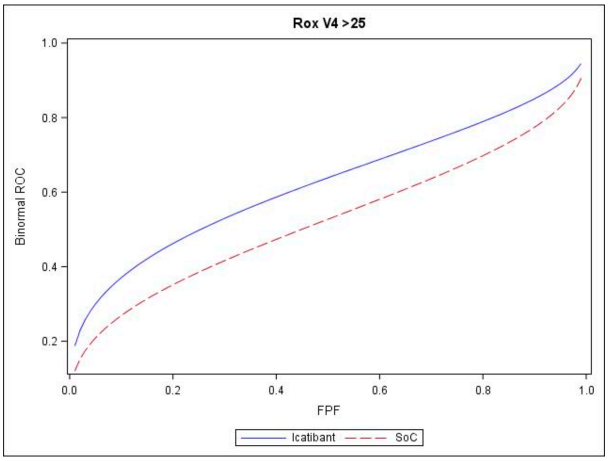

Icatibant treatment associates to a non-significant increase (p=0.459) of the discriminative ability of C1 esterase inhibitor (antigenic), going from an AUC of 0.524 in the SoC group to AUC of 0.623 with Icatibant

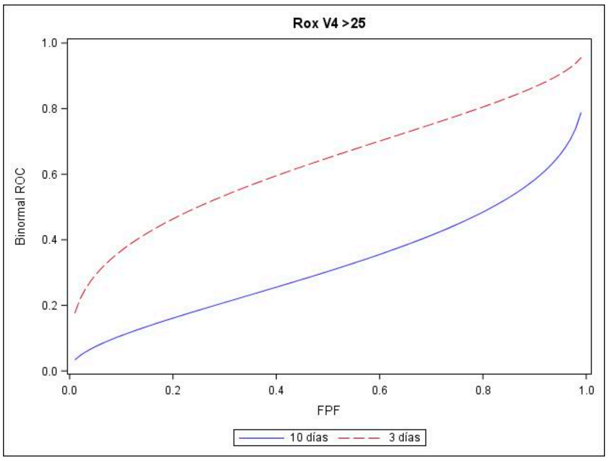

Time from symptom onset to hospitalization associates to a non-significant reduction (p=0.126) of the discriminative ability of C1 esterase inhibitor (antigenic), going from an AUC of 0.631 after 3 days to AUC of 0.327 after 10 days

Abbreviations: AUC, area under the curve; L, liter; mg, milligram; ROC, receiver operating characteristic; SoC, standard of care.

Figure S36: Concurrent criterion-type validity of the discriminative ability of C4 complement factor between patients who did and did not attain a median Sp_{O_2}/Fi_{O_2} ratio >380 at Day 4 (Visit 4), both unadjusted (empirical ROC curve) and adjusted by either Icatibant treatment or the time elapsed from symptom onset to hospitalization (binormal induced smooth ROC curves)

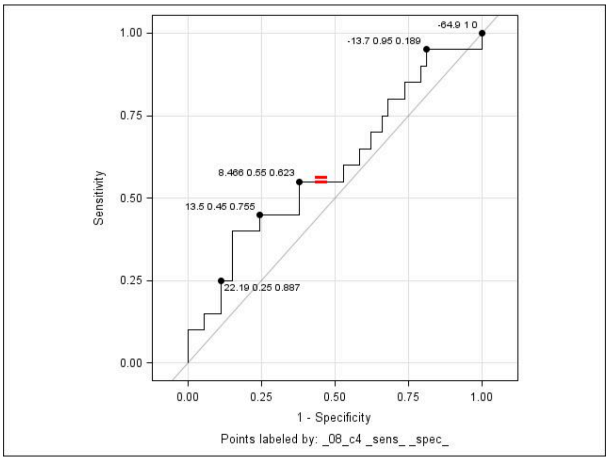

Levels of a representative patient on best cut-off score (baseline, Day 2, Day 3, Day 4):

297, 232, 251, 232 mg/L

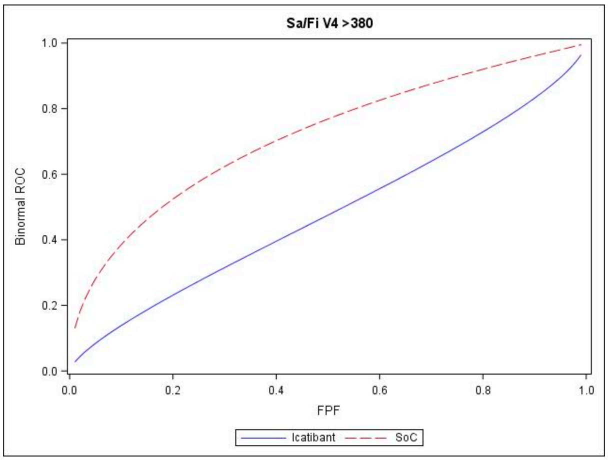

Icatibant treatment associates to an almost significant reduction (p=0.082) of the discriminative ability of C4 complement factor, going from an AUC of 0.717 in the SoC group to AUC of 0.481 with Icatibant

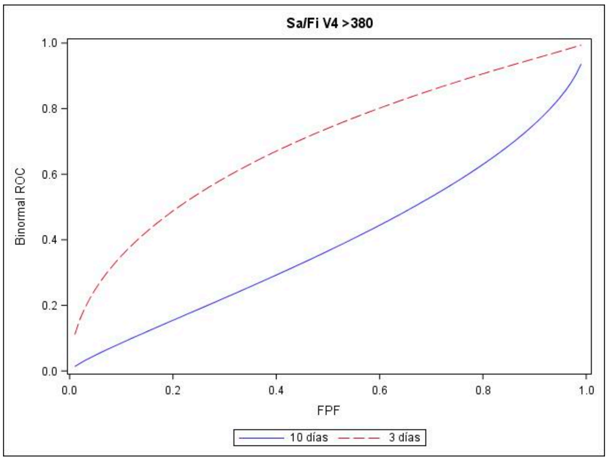

Time from symptom onset to hospitalization associates to a non-significant reduction (p=0.126) of the discriminative ability of C4 complement factor, going from an AUC of 0.692 after 3 days to AUC of 0.395 after 10 days

Abbreviations: AUC, area under the curve; L, liter; mg, milligram; ROC, receiver operating characteristic; SoC, standard of care.

Figure S37: Concurrent criterion-type validity of the discriminative ability of C4 complement factor between patients who did and did not attain a median Rox index >25 at Day 4 (Visit 4), both unadjusted (empirical ROC curve) and adjusted by either Icatibant treatment or the time elapsed from symptom onset to hospitalization (binormal induced smooth ROC curves)

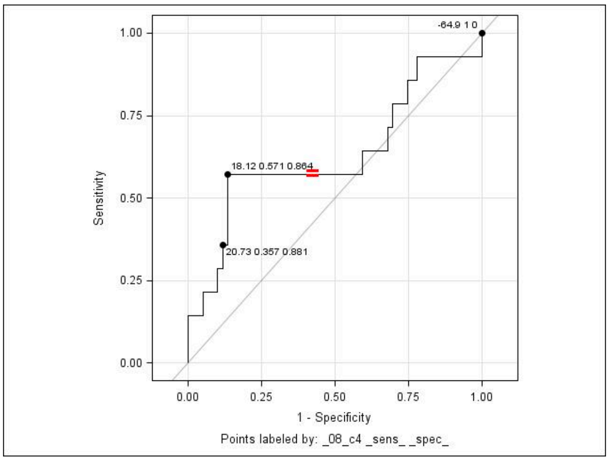

Levels of a representative patient on best cut-off score (baseline, Day 2, Day 3, Day 4):

420, 386, 380, 318 mg/L

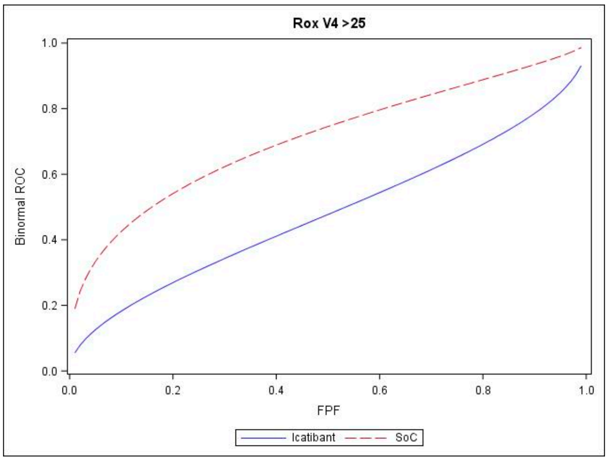

Icatibant treatment associates to a non-significant reduction (p=0.102) of the discriminative ability of C4 complement factor, going from an AUC of 0.709 in the SoC group to AUC of 0.481 with Icatibant

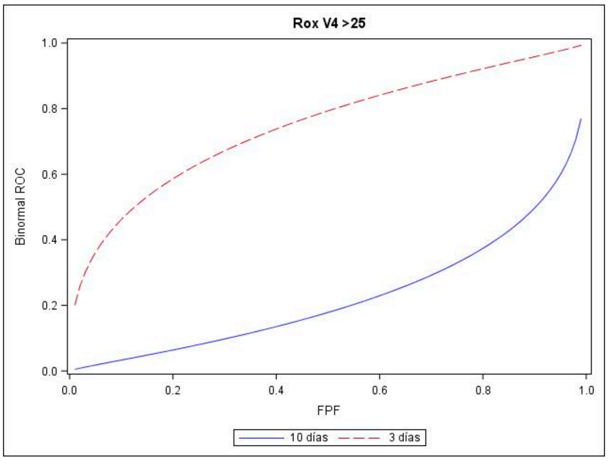

Time from symptom onset to hospitalization associates to a significant reduction (p=0.011) of the discriminative ability of C4 complement factor, going from an AUC of 0.747 after 3 days to AUC of 0.227 after 10 days

Abbreviations: AUC, area under the curve; L, liter; mg, milligram; ROC, receiver operating characteristic; SoC, standard of care.

Figure S38: Concurrent criterion-type validity of the discriminative ability of LDH to discern between patients who did and did not achieve a WHO status score scale <4 at Day 10 (Visit 5), both unadjusted (empirical ROC curve) and adjusted by either Icatibant treatment or the time elapsed from symptom onset to hospitalization (binormal induced smooth ROC curves)

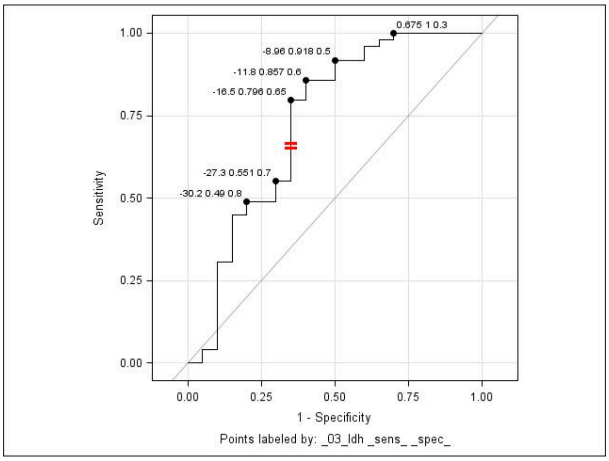

Levels of a representative patient on best cut-off score (baseline, Day 2, Day 3, Day 4):

378, 669, 374, 260 IU/L

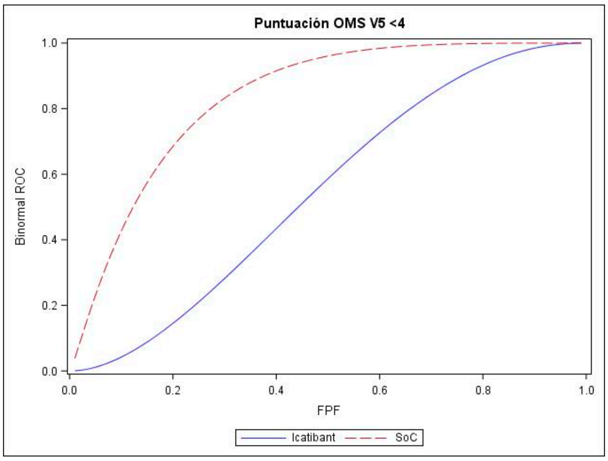

Icatibant treatment associates to a significant reduction (p=0.015) of the discriminative ability of LDH, going from an AUC of 0.834 in the SoC group to AUC of 0.548 with Icatibant

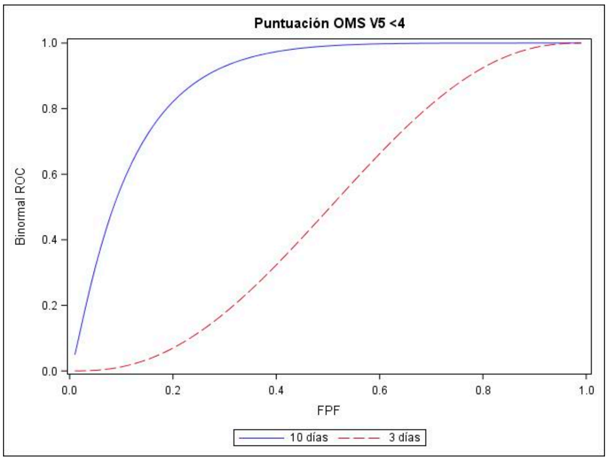

Time from symptom onset to hospitalization associates to a significant increase (p=0.007) of the discriminative ability of LDH, going from an AUC of 0.496 after 3 days to AUC of 0.883 after 10 days

Abbreviations: AUC, area under the curve; IU, international units; LDH, lactate dehydrogenase; L, liter; ROC, receiver operating characteristic; SoC, standard of care; WHO, World Health Organization.

Figure S39: Concurrent criterion-type validity of the discriminative ability of LDH to discern between patients who had and had not been discharged at Day 10 (Visit 5), both unadjusted (empirical ROC curve) and adjusted by either Icatibant treatment or the time elapsed from symptom onset to hospitalization (binormal induced smooth ROC curves)

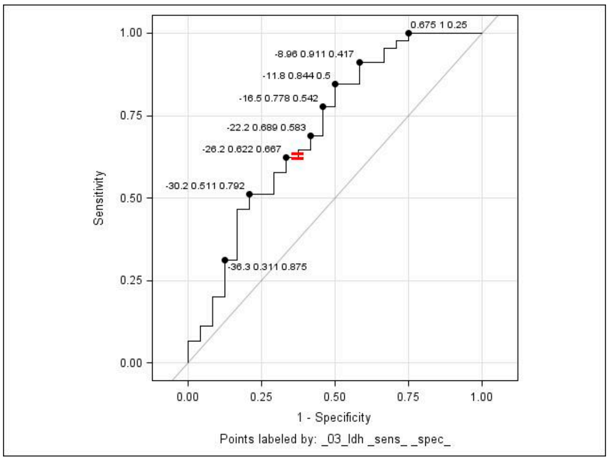

Levels of a representative patient on best cut-off score (baseline, Day 2, Day 3, Day 4):

601, 571, 572, 542 IU/L

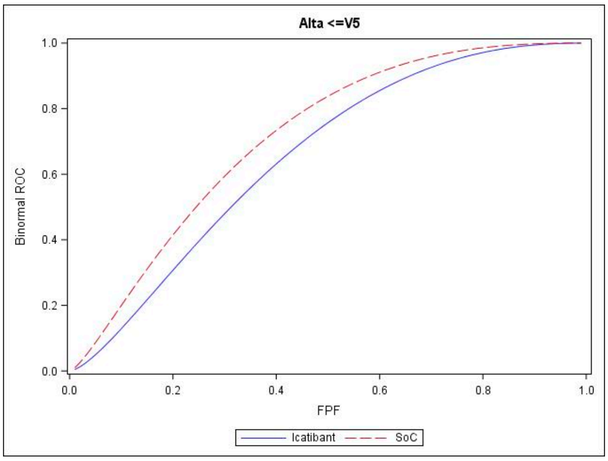

Icatibant treatment associates to a non-significant reduction (p=0.639) of the discriminative ability of LDH, going from an AUC of 0.714 in the SoC group to AUC of 0.656 with Icatibant

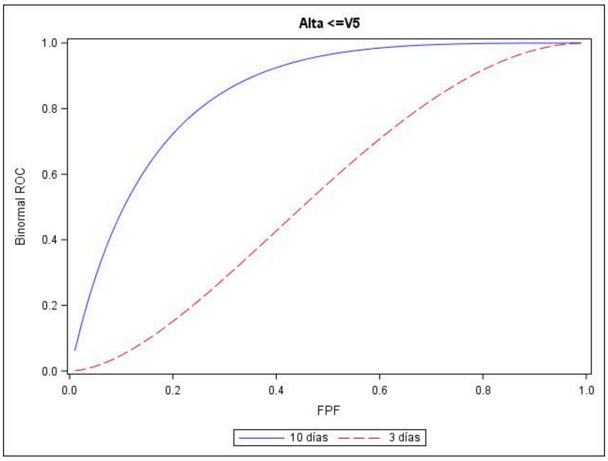

Time from symptom onset to hospitalization associates to a significant increase (p=0.047) of the discriminative ability of LDH, going from an AUC of 0.541 after 3 days to AUC of 0.848 after 10 days

Abbreviations: AUC, area under the curve; IU, international units; LDH, lactate dehydrogenase; L, liter; ROC, receiver operating characteristic; SoC, standard of care.

Figure S40: Concurrent criterion-type validity of the discriminative ability of LDH to discern between patients who did and did not attain a median Sp_{O_2}/Fi_{O_2} ratio >380 at Day 4 (Visit 4), both unadjusted (empirical ROC curve) and adjusted by either Icatibant treatment or the time elapsed from symptom onset to hospitalization (binormal induced smooth ROC curves)

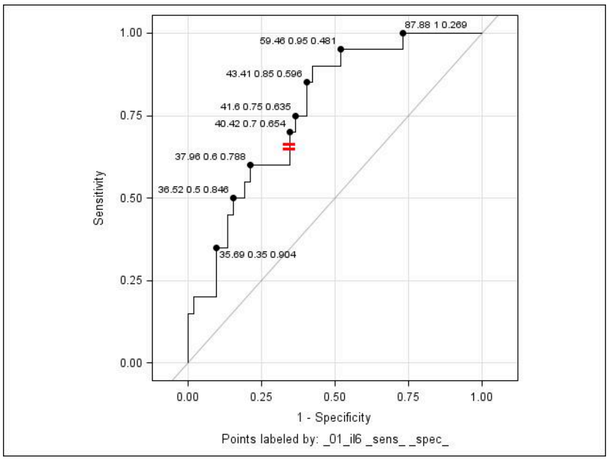

Levels of a representative patient on best cut-off score (baseline, Day 2, Day 3, Day 4):

185, 175, 196, 189 IU/L

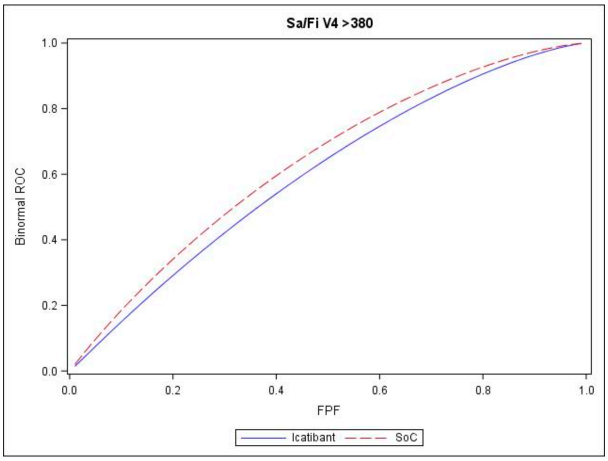

Icatibant treatment is not associated with apparent changes (p=0.815) of the discriminative ability of LDH, going from an AUC of 0.637 in the SoC group to AUC of 0.601 with Icatibant

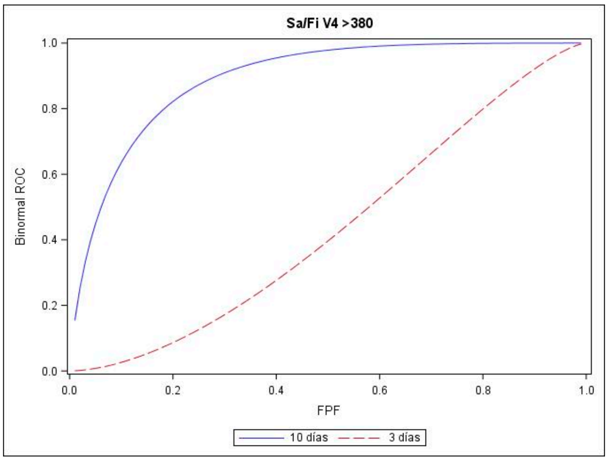

Time from symptom onset to hospitalization associates to a significant increase (p=0.014) of the discriminative ability of LDH, going from an AUC of 0.437 after 3 days to AUC of 0.890 after 10 days

Abbreviations: AUC, area under the curve; IU, international units; LDH, lactate dehydrogenase; L, liter; ROC, receiver operating characteristic; SoC, standard of care.

Figure S41: Concurrent criterion-type validity of the discriminative ability of LDH to discern between patients who did and did not attain a median Sp_{O_2}/Fi_{O_2} ratio >380 at Day 10 (Visit 5), both unadjusted (empirical ROC curve) and adjusted by either Icatibant treatment or the time elapsed from symptom onset to hospitalization (binormal induced smooth ROC curves)

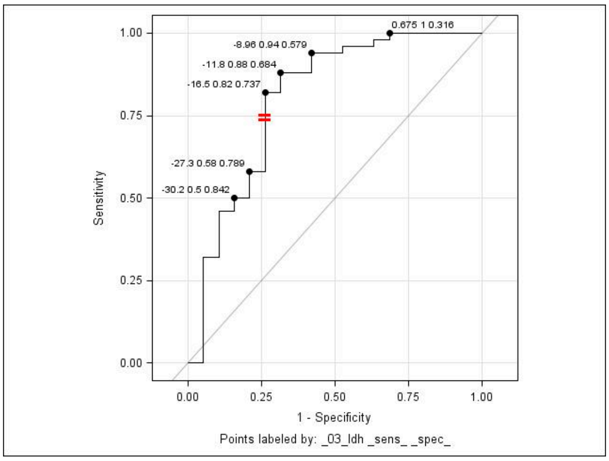

Levels of a representative patient on best cut-off score (baseline, Day 2, Day 3, Day 4):

511, 446, 442, 510 IU/L

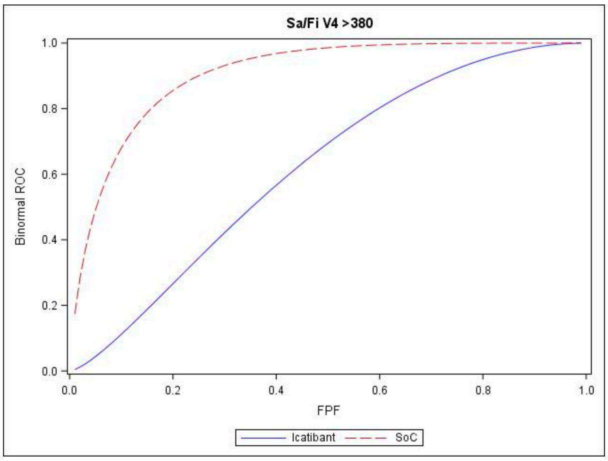

Icatibant treatment associates to a significant reduction (p=0.005) of the discriminative ability of LDH, going from an AUC of 0.904 in the SoC group to AUC of 0.619 with Icatibant

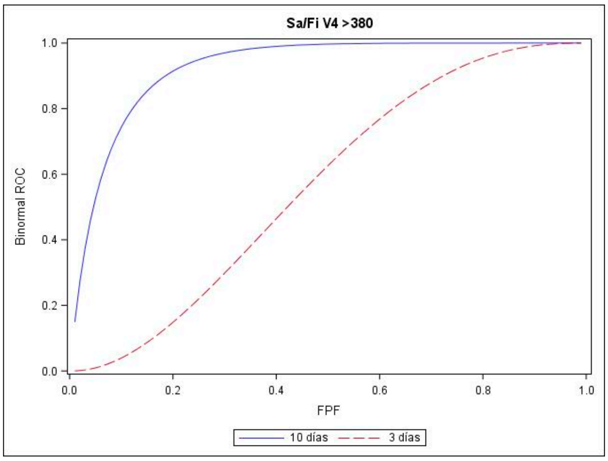

Time from symptom onset to hospitalization associates to a significant increase (p=0.005) of the discriminative ability of LDH, going from an AUC of 0.567 after 3 days to AUC of 0.924 after 10 days

Abbreviations: AUC, area under the curve; IU, international units; LDH, lactate dehydrogenase; L, liter; ROC, receiver operating characteristic; SoC, standard of care.

Figure S42: Concurrent criterion-type validity of the discriminative ability of LDH to discern between patients who did and did not attain a Rox index >25 at Day 10 (Visit 5), both unadjusted (empirical ROC curve) and adjusted by either Icatibant treatment or the time elapsed from symptom onset to hospitalization (binormal induced smooth ROC curves)

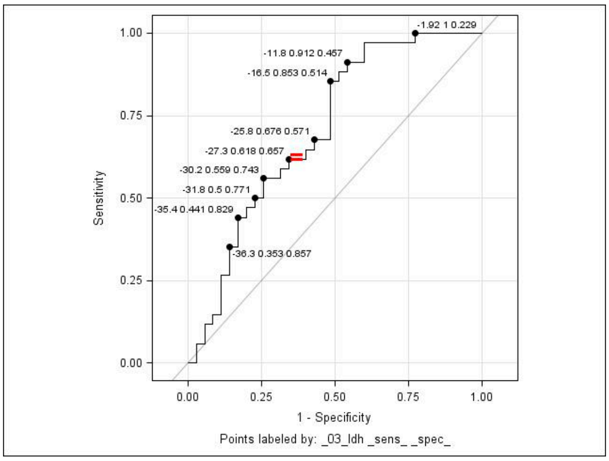

Levels of a representative patient on best cut-off score (baseline, Day 2, Day 3, Day 4):

168, 199, 162, 184 IU/L

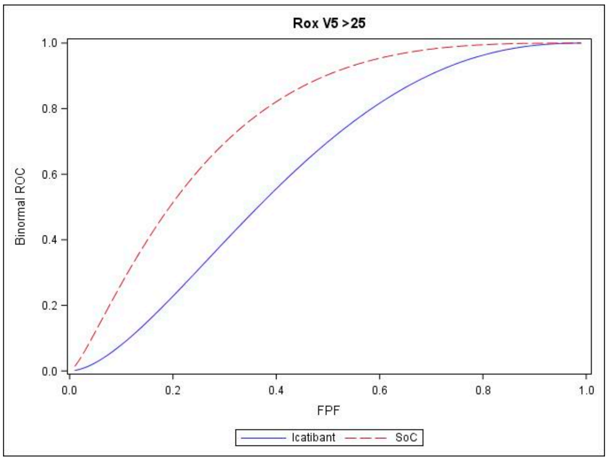

Icatibant treatment associates to a non-significant reduction (p=0.210) of the discriminative ability of LDH, going from an AUC of 0.764 in the SoC group to AUC of 0.614 with Icatibant

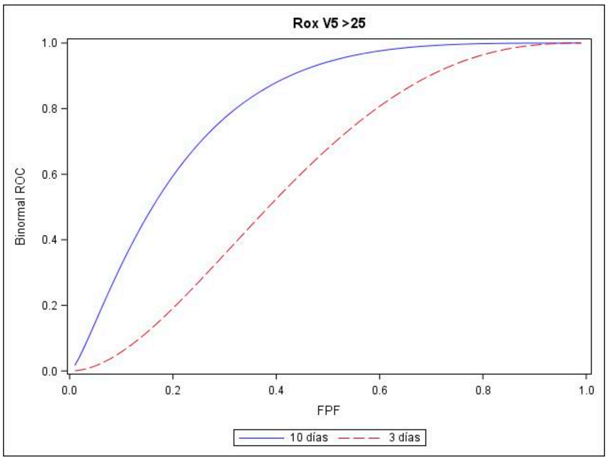

Time from symptom onset to hospitalization associates to a non-significant increase (p=0.220) of the discriminative ability of LDH, going from an AUC of 0.598 after 3 days to AUC of 0.800 after 10 days

Abbreviations: AUC, area under the curve; IU, international units; LDH, lactate dehydrogenase; L, liter; ROC, receiver operating characteristic; SoC, standard of care.

Figure S43: Concurrent criterion-type validity of the discriminative ability of LDH to discern between patients who did and did not attain a clinical response at Day 10 (Visit 5), both unadjusted (empirical ROC curve) and adjusted by either Icatibant treatment or the time elapsed from symptom onset to hospitalization (binormal induced smooth ROC curves)

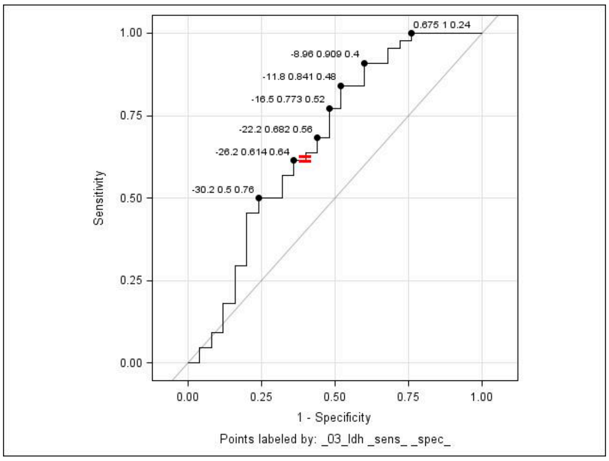

Levels of a representative patient on best cut-off score (baseline, Day 2, Day 3, Day 4): 601, 571, 572, 542 IU/L

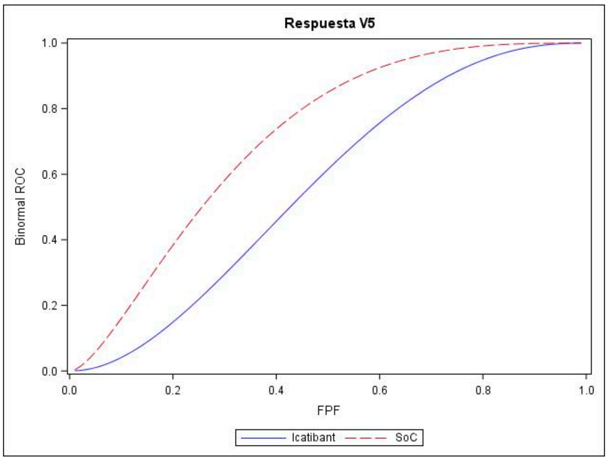

Icatibant treatment associates to a non-significant reduction (p=0.246) of the discriminative ability of LDH, going from an AUC of 0.710 in the SoC group to AUC of 0.562 with Icatibant

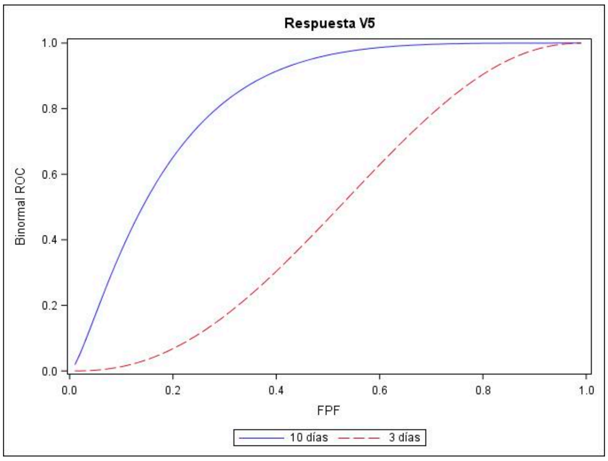

Time from symptom onset to hospitalization associates to a significant increase (p=0.039) of the discriminative ability of LDH, going from an AUC of 0.481 after 3 days to AUC of 0.822 after 10 days

Abbreviations: AUC, area under the curve; IU, international units; LDH, lactate dehydrogenase; L, liter; ROC, receiver operating characteristic; SoC, standard of care.

Figure S44: Concurrent criterion-type validity of the discriminative ability of LDH to discern between patients who did and did not attain a clinical response 28 days after initial discharge, both unadjusted (empirical ROC curve) and adjusted by either Icatibant treatment or the time elapsed from symptom onset to hospitalization (binormal induced smooth ROC curves)

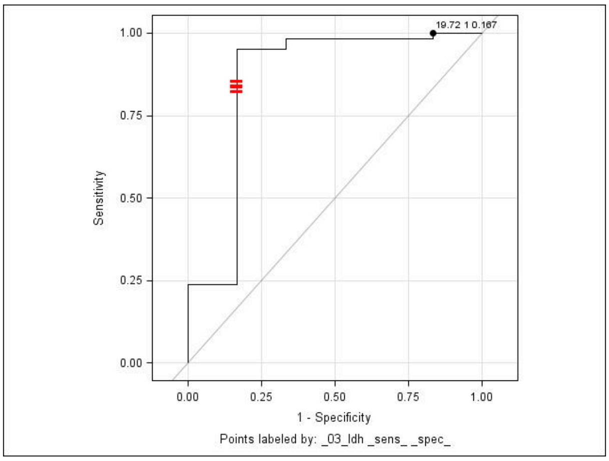

Levels of a representative patient on best cut-off score (baseline, Day 2, Day 3, Day 4):

295, 360, 323, 353 IU/L

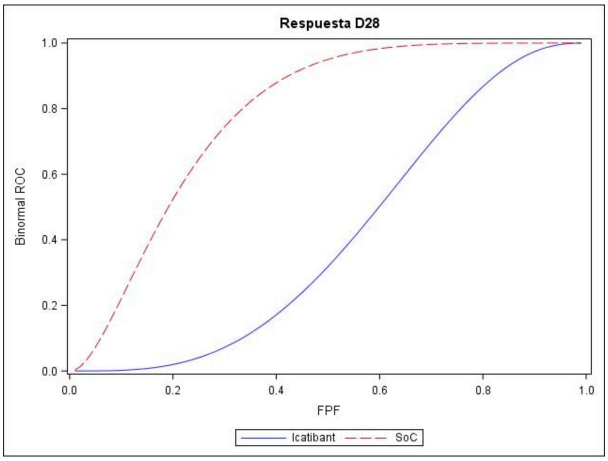

Icatibant treatment associates to a non-significant reduction (Bayes, doubtful convergence) of the discriminative ability of LDH, going from an AUC of 0.780 in the SoC group to AUC of 0.412 with Icatibant

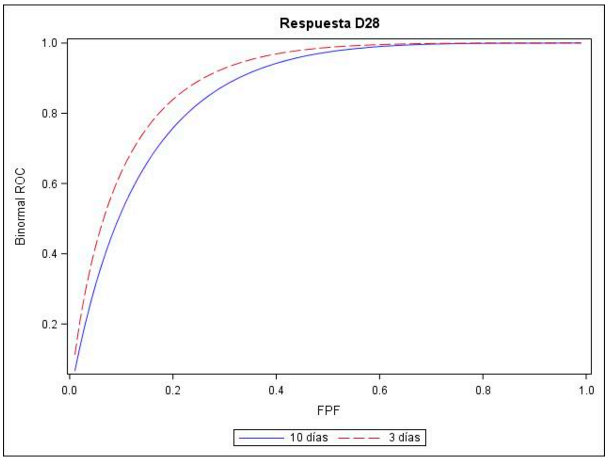

Time from symptom onset to hospitalization is not associated with apparent changes (p=0.831) of the discriminative ability of LDH, going from an AUC of 0.895 after 3 days to AUC of 0.862 after 10 days

Abbreviations: AUC, area under the curve; IU, international units; LDH, lactate dehydrogenase; L, liter; ROC, receiver operating characteristic; SoC, standard of care.

Figure S45: Concurrent criterion-type validity of the discriminative ability of LDH to discern patients who died from COVID-19, both unadjusted (empirical ROC curve) and adjusted by either Icatibant treatment or the time elapsed from symptom onset to hospitalization (binormal induced smooth ROC curves)

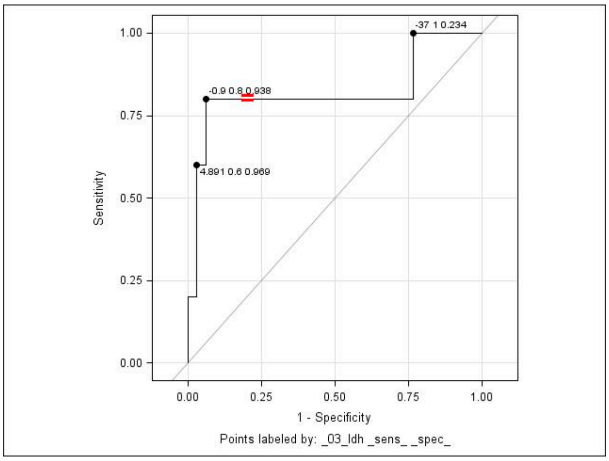

Levels of a representative patient on best cut-off score (baseline, Day 2, Day 3, Day 4):

267, 325, 309, 318 IU/L

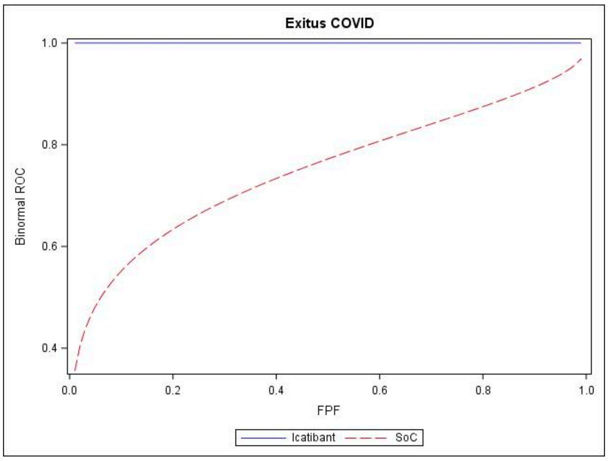

Icatibant treatment associates to an almost significant increase (Bayes, doubtful convergence) of the discriminative ability of LDH, going from an AUC of 0.749 in the SoC group to AUC of 1.000 with Icatibant

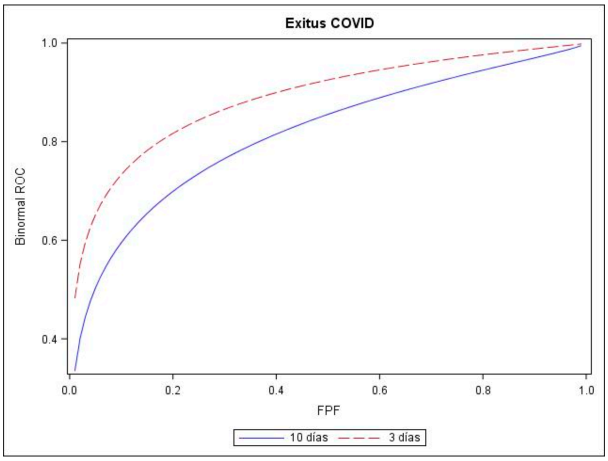

Time from symptom onset to hospitalization associates to a non-significant reduction (p=0.674) of the discriminative ability of LDH, going from an AUC of 0.888 after 3 days to AUC of 0.814 after 10 days

Abbreviations: AUC, area under the curve; IU, international units; LDH, lactate dehydrogenase; L, liter; ROC, receiver operating characteristic; SoC, standard of care.

Figure S46: Concurrent criterion-type validity of the discriminative ability of LDH to discern patients who died from any reason, both unadjusted (empirical ROC curve) and adjusted by either Icatibant treatment or the time elapsed from symptom onset to hospitalization (binormal induced smooth ROC curves)

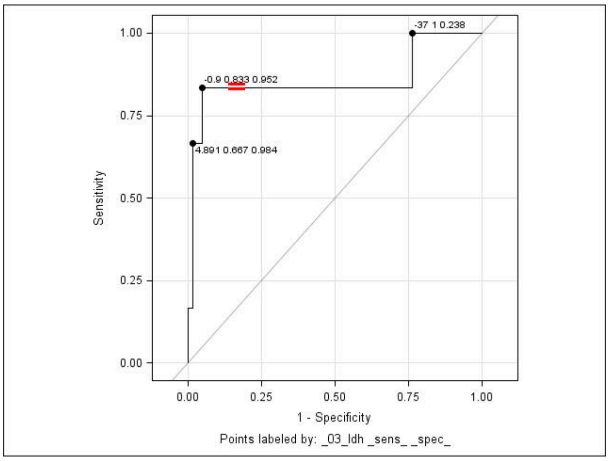

Levels of a representative patient on best cut-off score (baseline, Day 2, Day 3, Day 4):

295, 360, 323, 353 IU/L

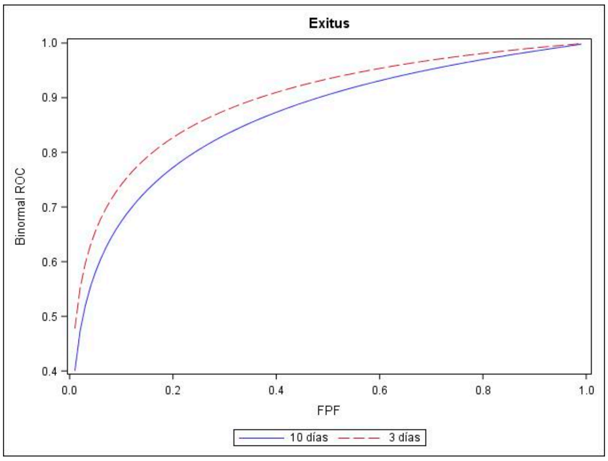

Model did not converge after having checked multiple settings and alternatives for approximation

Time from symptom onset to hospitalization is not associated with apparent changes (p=0.831) of the discriminative ability of LDH, going from an AUC of 0.895 after 3 days to AUC of 0.861 after 10 days

Abbreviations: AUC, area under the curve; IU, international units; LDH, lactate dehydrogenase; L, liter; ROC, receiver operating characteristic; SoC, standard of care.

Figure S47: Concurrent criterion-type validity of the discriminative ability of lymphocytes to discern between patients who did and did not achieve a WHO status score scale <4 at Day 10 (Visit 5), both unadjusted (empirical ROC curve) and adjusted by either Icatibant treatment or the time elapsed from symptom onset to hospitalization (binormal induced smooth ROC curves)

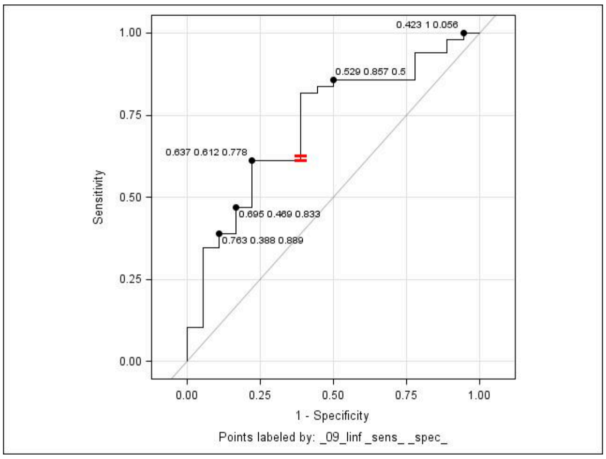

Levels of a representative patient on best cut-off score (baseline, Day 2, Day 3, Day 4):

0.93, 0.86, 0.85, 0.98 cells/nL

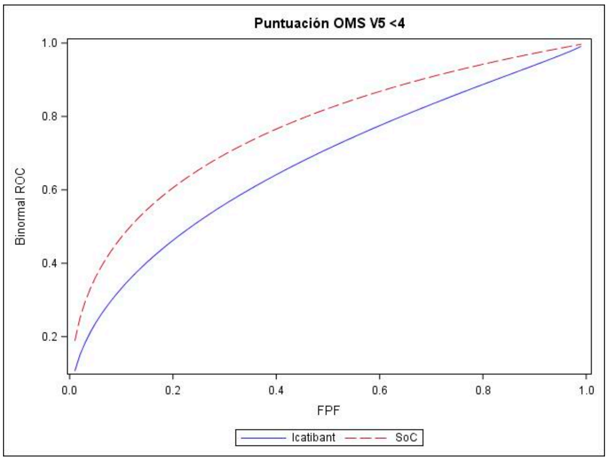

Icatibant treatment associates to a non-significant reduction (p=0.495) of the discriminative ability of lymphocytes, going from an AUC of 0.767 in the SoC group to AUC of 0.671 with Icatibant

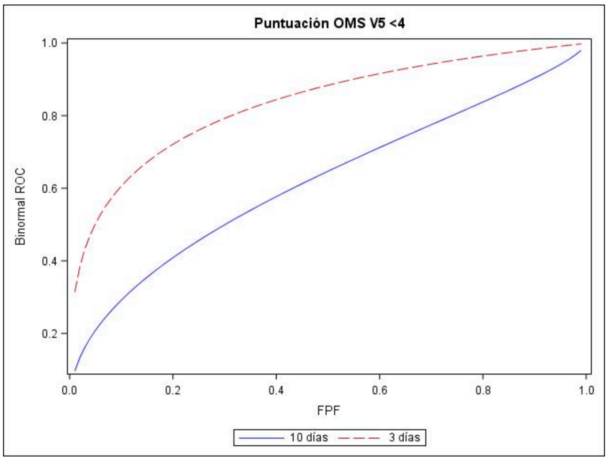

Time from symptom onset to hospitalization associates to a non-significant reduction (p=0.222) of the discriminative ability of lymphocytes, going from an AUC of 0.833 after 3 days to AUC of 0.620 after 10 days

Abbreviations: AUC, area under the curve; nL, nanoliter; ROC, receiver operating characteristic; SoC, standard of care; WHO, World Health Organization.

Figure S48: Concurrent criterion-type validity of the discriminative ability of lymphocytes to discern between patients who had and had not been discharged at Day 10 (Visit 5), both unadjusted (empirical ROC curve) and adjusted by either Icatibant treatment or the time elapsed from symptom onset to hospitalization (binormal induced smooth ROC curves)

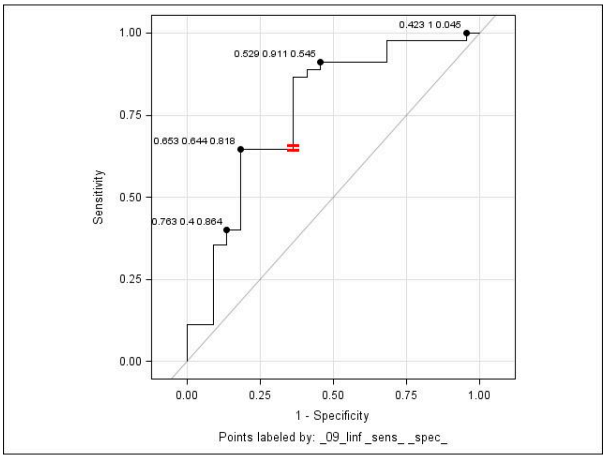

Levels of a representative patient on best cut-off score (baseline, Day 2, Day 3, Day 4):

0.93, 0.86, 0.85, 0.98 cells/nL

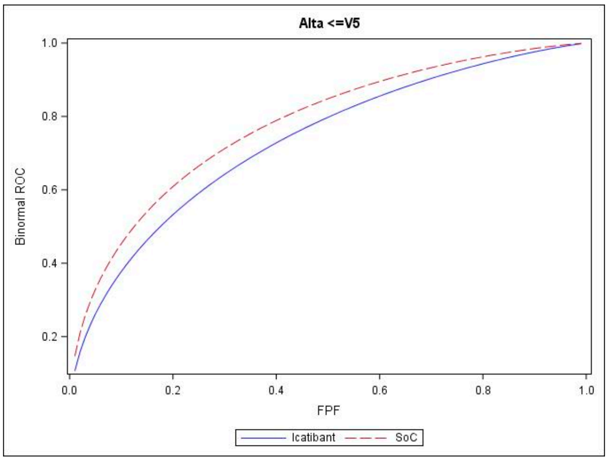

Icatibant treatment is not associated with apparent changes (p=0.713) of the discriminative ability of lymphocytes, going from an AUC of 0.779 in the SoC group to AUC of 0.733 with Icatibant

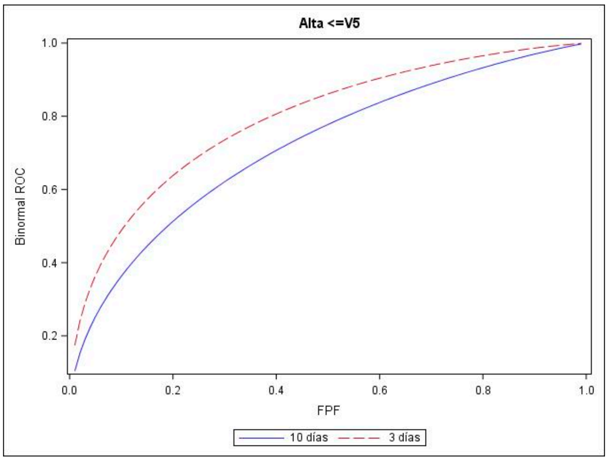

Time from symptom onset to hospitalization associates to a non-significant reduction (p=0.637) of the discriminative ability of lymphocytes, going from an AUC of 0.794 after 3 days to AUC of 0.718 after 10 days

Abbreviations: AUC, area under the curve; nL, nanoliter; ROC, receiver operating characteristic; SoC, standard of care.

Figure S49: Concurrent criterion-type validity of the discriminative ability of lymphocytes to discern between patients who did and did not attain a median Sp_{O_2}/Fi_{O_2} ratio >380 at Day 4 (Visit 4), both unadjusted (empirical ROC curve) and adjusted by either Icatibant treatment or the time elapsed from symptom onset to hospitalization (binormal induced smooth ROC curves)

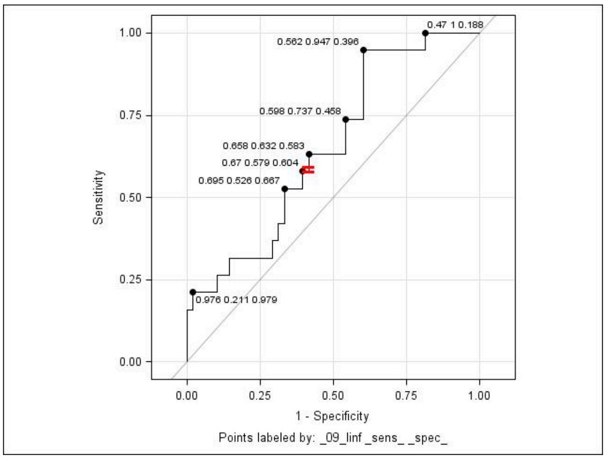

Levels of a representative patient on best cut-off score (baseline, Day 2, Day 3, Day 4):

1.47, 1.72, 1.81, 1.5 cells/nL

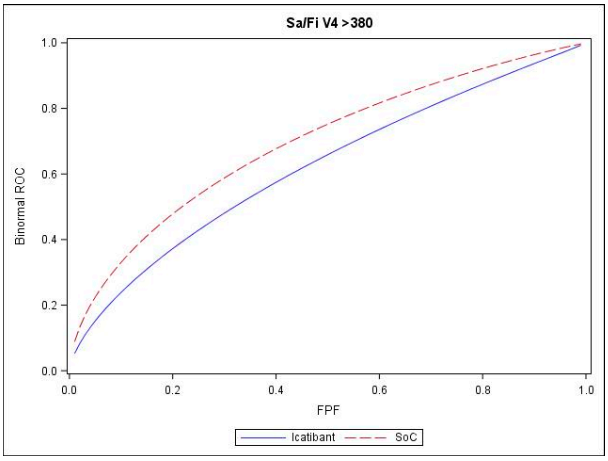

Icatibant treatment associates with a non-significant reduction (p=0.587) of the discriminative ability of lymphocytes, going from an AUC of 0.696 in the SoC group to AUC of 0.621 with Icatibant

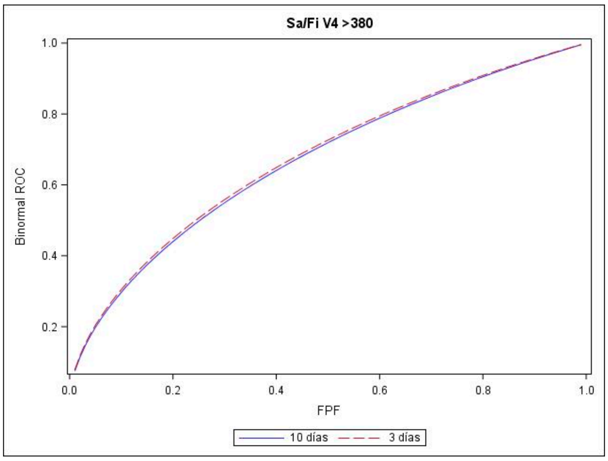

Time from symptom onset to hospitalization is not associated with apparent changes (p=0.975) of the discriminative ability of lymphocytes, going from an AUC of 0.675 after 3 days to AUC of 0.670 after 10 days

Abbreviations: AUC, area under the curve; nL, nanoliter; ROC, receiver operating characteristic; SoC, standard of care.

Figure S50: Concurrent criterion-type validity of the discriminative ability of lymphocytes to discern between patients who did and did not attain a median Sp_{O_2}/Fi_{O_2} ratio >380 at Day 10 (Visit 5), both unadjusted (empirical ROC curve) and adjusted by either Icatibant treatment or the time elapsed from symptom onset to hospitalization (binormal induced smooth ROC curves)

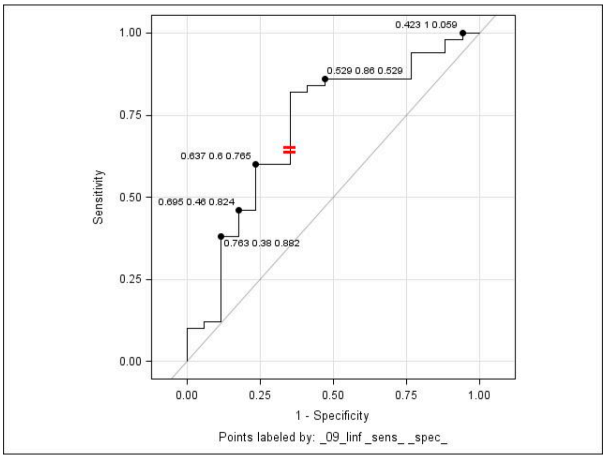

Levels of a representative patient on best cut-off score (baseline, Day 2, Day 3, Day 4):

0.93, 0.86, 0.85, 0.98 cells/nL

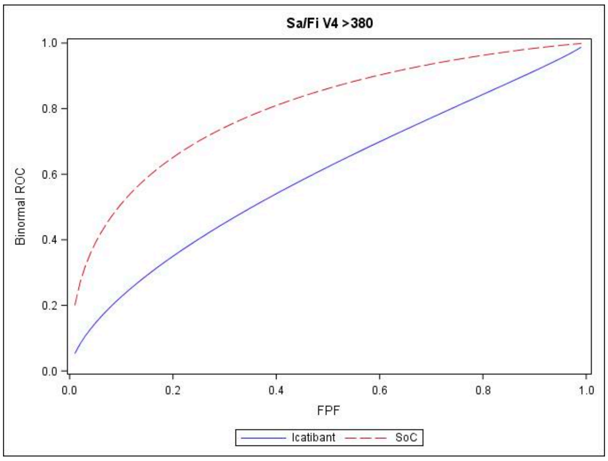

Icatibant treatment associates with a non-significant reduction (p=0.156) of the discriminative ability of lymphocytes, going from an AUC of 0.799 in the SoC group to AUC of 0.595 with Icatibant

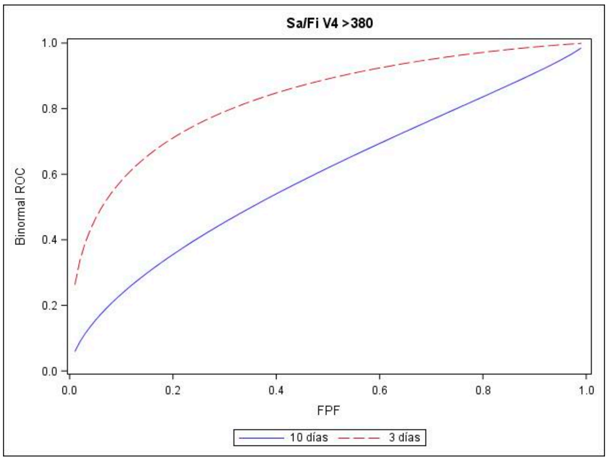

Time from symptom onset to hospitalization associates to a non-significant reduction (p=0.177) of the discriminative ability of lymphocytes, going from an AUC of 0.831 after 3 days to AUC of 0.594 after 10 days

Abbreviations: AUC, area under the curve; nL, nanoliter; ROC, receiver operating characteristic; SoC, standard of care.

Figure S51: Concurrent criterion-type validity of the discriminative ability of lymphocytes to discern between patients who did and did not attain a median Rox index >25 at Day 4 (Visit 4), both unadjusted (empirical ROC curve) and adjusted by either Icatibant treatment or the time elapsed from symptom onset to hospitalization (binormal induced smooth ROC curves)

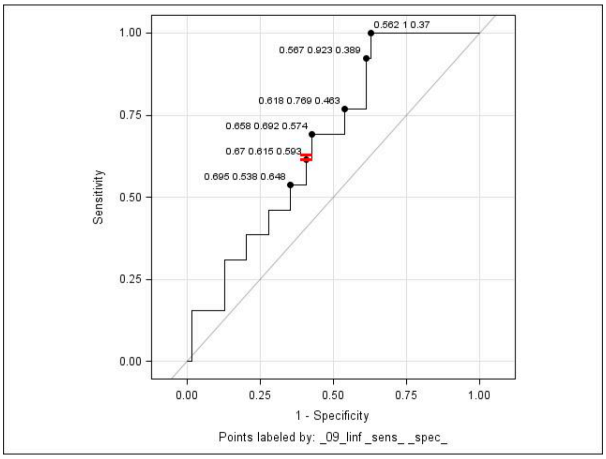

Levels of a representative patient on best cut-off score (baseline, Day 2, Day 3, Day 4):

1.1, 1.04, 1.28, 1.64 cells/nL

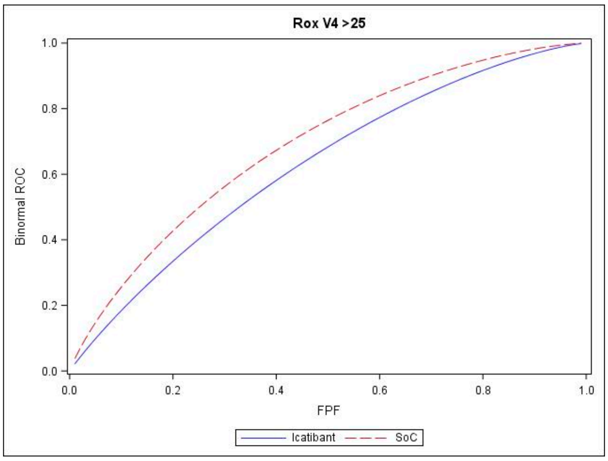

Icatibant treatment is not associated with apparent changes (p=0.711) of the discriminative ability of lymphocytes, going from an AUC of 0.688 in the SoC group to AUC of 0.627 with Icatibant

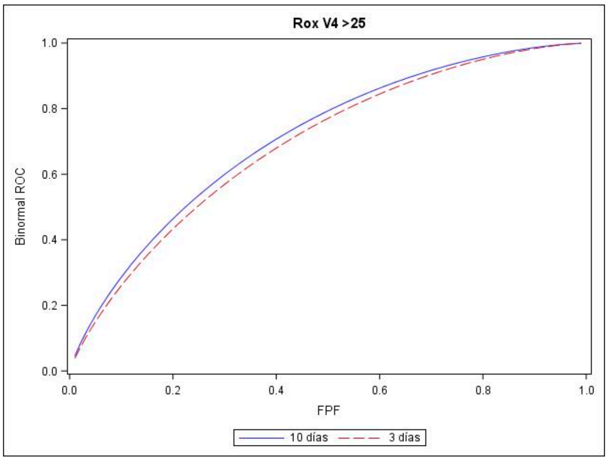

Time from symptom onset to hospitalization is not associated with apparent changes (p=0.938) of the discriminative ability of lymphocytes, going from an AUC of 0.693 after 3 days to AUC of 0.711 after 10 days

Abbreviations: AUC, area under the curve; nL, nanoliter; ROC, receiver operating characteristic; SoC, standard of care.

Figure S52: Concurrent criterion-type validity of the discriminative ability of lymphocytes to discern between patients who did and did not attain a median Rox index >25 at Day 10 (Visit 5), both unadjusted (empirical ROC curve) and adjusted by either Icatibant treatment or the time elapsed from symptom onset to hospitalization (binormal induced smooth ROC curves)

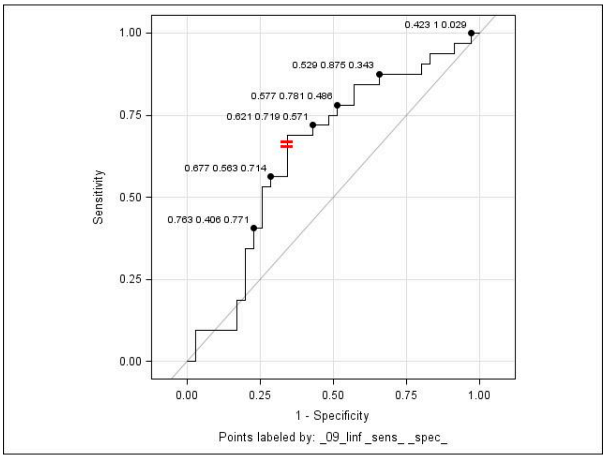

Levels of a representative patient on best cut-off score (baseline, Day 2, Day 3, Day 4):

0.12, 0.33, 0.47, 0.8 cells/nL

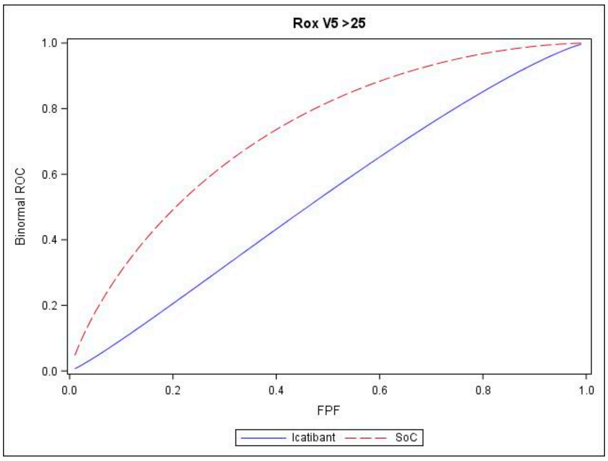

Icatibant treatment associates to a non-significant reduction (p=0.127) of the discriminative ability of lymphocytes, going from an AUC of 0.729 in the SoC group to AUC of 0.530 with Icatibant

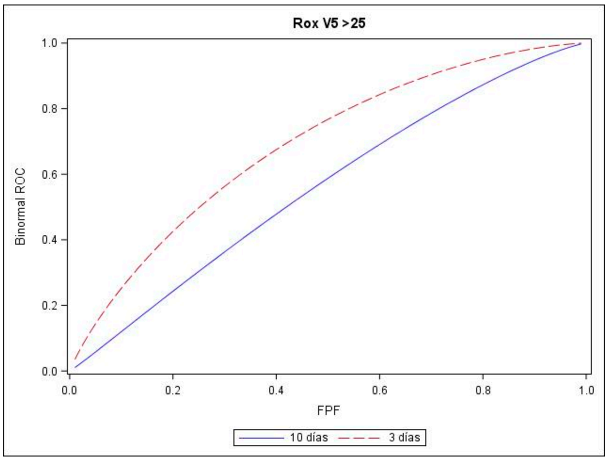

Time from symptom onset to hospitalization associates to a non-significant reduction (p=0.488) of the discriminative ability of lymphocytes, going from an AUC of 0.689 after 3 days to AUC of 0.560 after 10 days

Abbreviations: AUC, area under the curve; nL, nanoliter; ROC, receiver operating characteristic; SoC, standard of care.

Figure S53: Concurrent criterion-type validity of the discriminative ability of lymphocytes to discern between patients who did and did not attain a clinical response at Day 10 (Visit 5), both unadjusted (empirical ROC curve) and adjusted by either Icatibant treatment or the time elapsed from symptom onset to hospitalization (binormal induced smooth ROC curves)

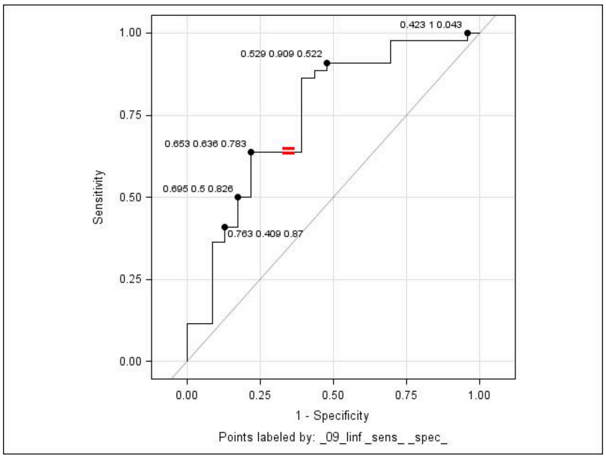

Levels of a representative patient on best cut-off score (baseline, Day 2, Day 3, Day 4):

0.93, 0.86, 0.85, 0.98 cells/nL

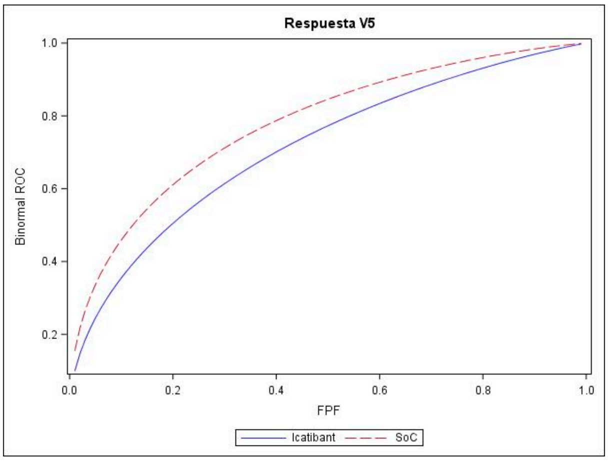

Icatibant treatment associates to a non-significant reduction (p=0.599) of the discriminative ability of lymphocytes, going from an AUC of 0.778 in the SoC group to AUC of 0.713 with Icatibant

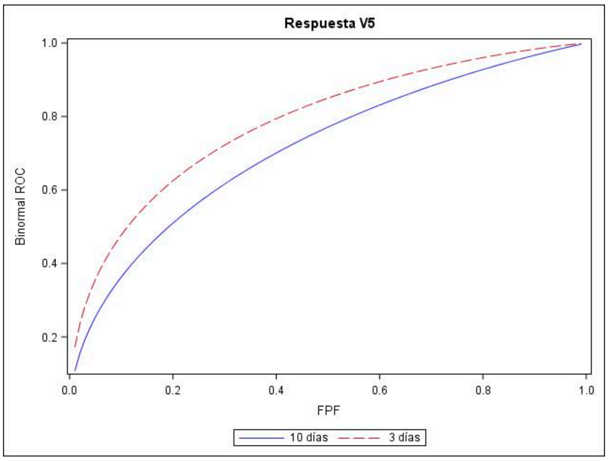

Time from symptom onset to hospitalization associates to a non-significant reduction (p=0.662) of the discriminative ability of lymphocytes, going from an AUC of 0.785 after 3 days to AUC of 0.714 after 10 days

Abbreviations: AUC, area under the curve; nL, nanoliter; ROC, receiver operating characteristic; SoC, standard of care.

Figure S54: Concurrent criterion-type validity of the discriminative ability of lymphocytes to discern between patients who did and did not attain a clinical response 28 days after initial discharge, both unadjusted (empirical ROC curve) and adjusted by either Icatibant treatment or the time elapsed from symptom onset to hospitalization (binormal induced smooth ROC curves)

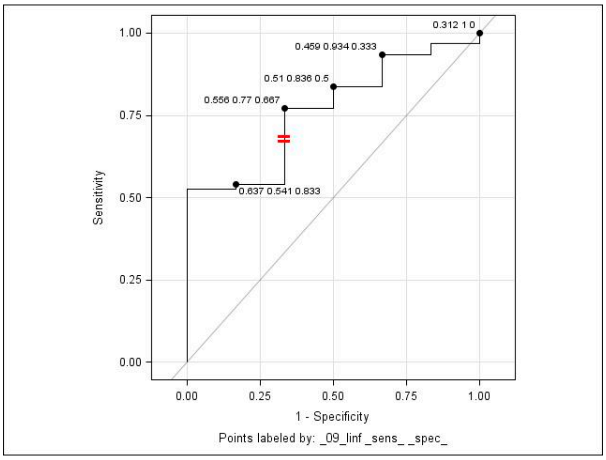

Levels of a representative patient on best cut-off score (baseline, Day 2, Day 3, Day 4):

1.32, 1.33, 0.93, 1.45 cells/nL

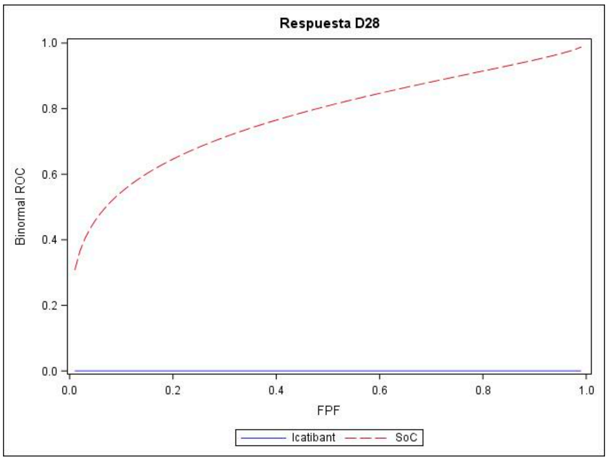

Icatibant treatment associates to a non-significant reduction (Bayes, doubtful convergence) of the discriminative ability of lymphocytes, going from an AUC of 0.774 in the SoC group to AUC of 0.000 with Icatibant

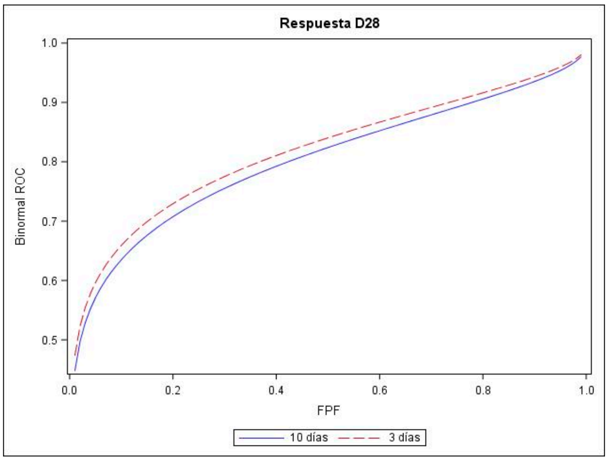

Time from symptom onset to hospitalization is not associated with apparent changes (p=0.959) of the discriminative ability of lymphocytes, going from an AUC of 0.817 after 3 days to AUC of 0.801 after 10 days

Abbreviations: AUC, area under the curve; nL, nanoliter; ROC, receiver operating characteristic; SoC, standard of care.

Figure S55: Concurrent criterion-type validity of the discriminative ability of lymphocytes to discern between patients who died from COVID-19, both unadjusted (empirical ROC curve) and adjusted by either Icatibant treatment or the time elapsed from symptom onset to hospitalization (binormal induced smooth ROC curves)

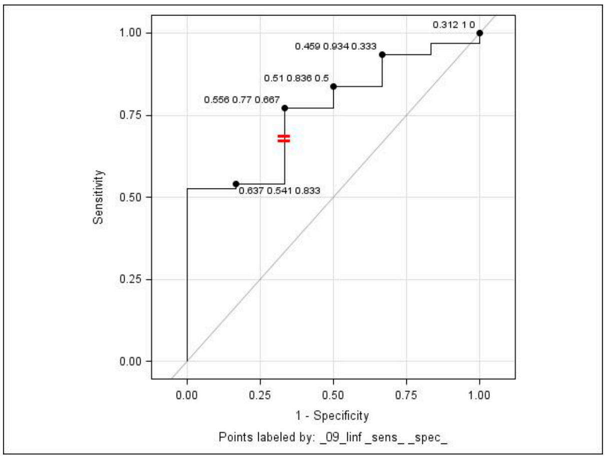

Levels of a representative patient on best cut-off score (baseline, Day 2, Day 3, Day 4):

1.32, 1.33, 0.93, 1.45 cells/nL

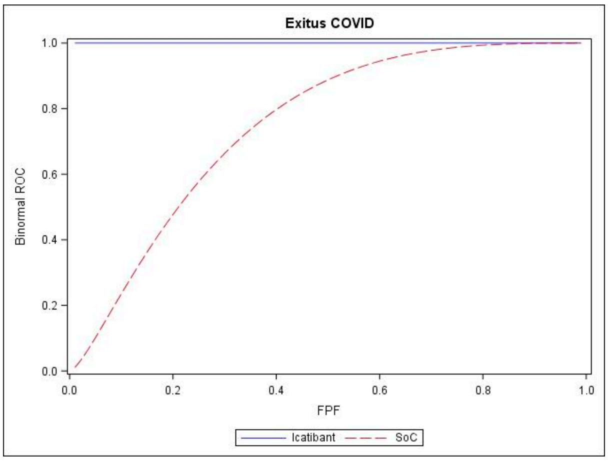

Icatibant treatment associates to a non-significant increase (Bayes, doubtful convergence) of the discriminative ability of lymphocytes, going from an AUC of 0.747 in the SoC group to AUC of 1.000 with Icatibant

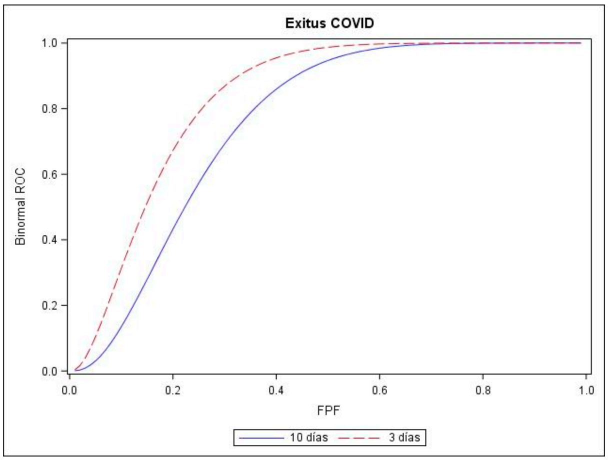

Time from symptom onset to hospitalization is not associated with apparent changes (p=0.825) of the discriminative ability of lymphocytes, going from an AUC of 0.829 after 3 days to AUC of 0.755 after 10 days

Abbreviations: AUC, area under the curve; nL, nanoliter; ROC, receiver operating characteristic; SoC, standard of care.

Figure S56: Concurrent criterion-type validity of the discriminative ability of lymphocytes to discern between patients who died from any reason, both unadjusted (empirical ROC curve) and adjusted by either Icatibant treatment or the time elapsed from symptom onset to hospitalization (binormal induced smooth ROC curves)

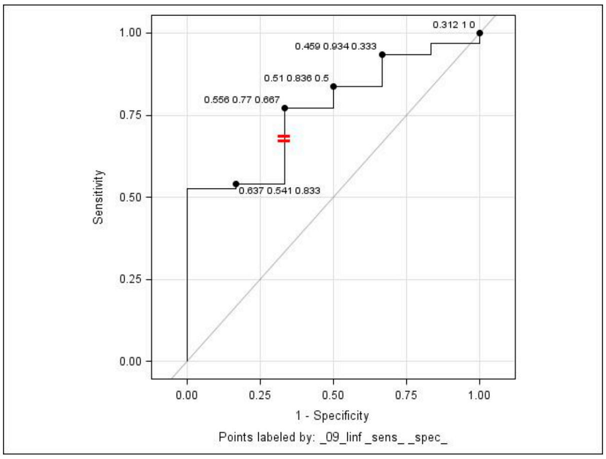

Levels of a representative patient on best cut-off score (baseline, Day 2, Day 3, Day 4):

1.32, 1.33, 0.93, 1.45 cells/nL

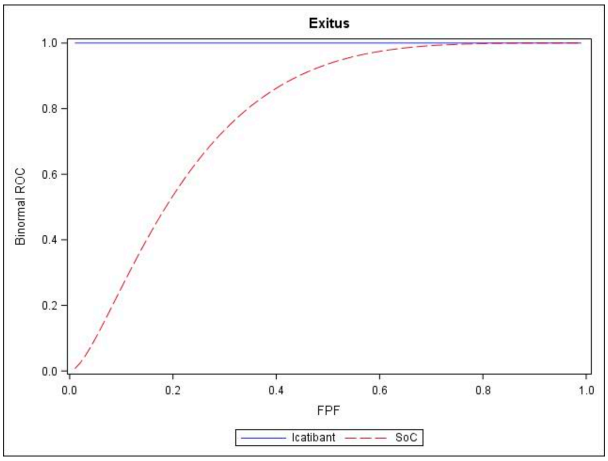

Icatibant treatment associates to an almost significant increase (Bayes, doubtful convergence) of the discriminative ability of lymphocytes, going from an AUC of 0.779 in the SoC group to AUC of 1.000 with Icatibant

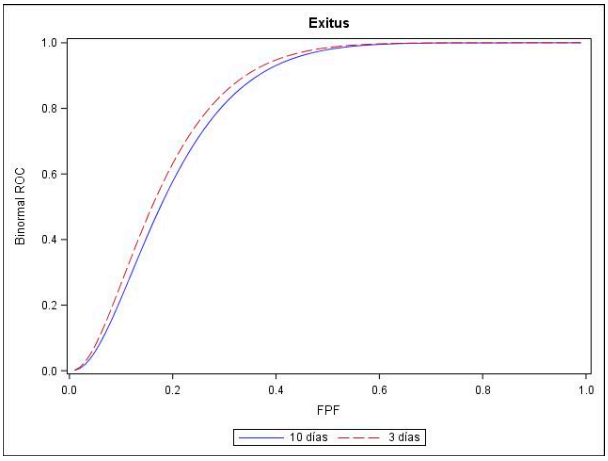

Time from symptom onset to hospitalization is not associated with apparent changes (p=0.959) of the discriminative ability of lymphocytes, going from an AUC of 0.817 after 3 days to AUC of 0.801 after 10 days

Abbreviations: AUC, area under the curve; nL, nanoliter; ROC, receiver operating characteristic; SoC, standard of care.

References

1. Pepe, M.S. Covariate effects on continuous and ordinal tests. In *The Statistical Evaluation of Medical Tests for Classification and Prediction*; Publisher: Oxford University Press, New York, NY, 2003; pp. 130-167.

2. Pepe, M.S. The binormal ROC curve. In *The Statistical Evaluation of Medical Tests for Classification and Prediction*; Publisher: Oxford University Press, New York, NY, 2003; pp. 81-84.

3. Malchair, P., Giol, J., Garcia, V., Rodriguez, O., Ruibal, J.C., Zarauza, A., Llopis, F., Matellan, L., Bernal, T., Solis, B., Otero, A., Carnaval, T., Jofre, H., Jacob, J., Solanich, X., Antoli, A., Rocamora, G., Videla, S. Three-Day Icatibant on Top of Standard Care in Patients With Coronavirus Disease 2019 Pneumonia: A Randomized, Open-Label, Phase 2, Proof-of-Concept Trial. *Clin Infect Dis* **2023**, *76*, 1784-1792.
